# Genome-wide human brain eQTLs: In-depth analysis and insights using the UKBEC dataset

**DOI:** 10.1038/s41598-019-55590-0

**Published:** 2019-12-16

**Authors:** Letitia M. F. Sng, Peter C. Thomson, Daniah Trabzuni

**Affiliations:** 1 The University of Sydney, School of Life and Environmental Sciences, New South Wales, 2006 Australia; 2 Department of Neurodegenerative Diseases, UCL Queen Square Institute of Neurology, Queen Square, London, WC1N 3BG UK; 3 Department of Genetics, King Faisal Specialist Hospital and Research Centre, 11211 Riyadh, Saudi Arabia

**Keywords:** Gene expression, Genome-wide association studies, Neuroscience

## Abstract

Understanding the complexity of the human brain transcriptome architecture is one of the most important human genetics study areas. Previous studies have applied expression quantitative trait loci (eQTL) analysis at the genome-wide level of the brain to understand the underlying mechanisms relating to neurodegenerative diseases, primarily at the transcript level. To increase the resolution of our understanding, the current study investigates multi/single-region, transcript/exon-level and *cis* versus *trans*-acting eQTL, across 10 regions of the human brain. Some of the key findings of this study are: (i) only a relatively small proportion of eQTLs will be detected, where the sensitivity is under 5%; (ii) when an eQTL is acting in multiple regions (MR-eQTL), it tends to have very similar effects on gene expression in each of these regions, as well as being *cis*-acting; (iii) *trans*-acting eQTLs tend to have larger effects on expression compared to *cis*-acting eQTLs and tend to be specific to a single region (SR-eQTL) of the brain; (iv) the cerebellum has a very large number of eQTLs that function exclusively in this region, compared with other regions of the brain; (v) importantly, an interactive visualisation tool (Shiny app) was developed to visualise the MR/SR-eQTL at transcript and exon levels.

## Introduction

The difficulty and complexity of studying the brain transcriptome architecture arises from the nature of the human brain as a heterogeneous structure containing different cell types at different ratios in different anatomical regions^1^. It has been reported that the variability in the transcription profiles of the human CNS can lead to different functional features^2^. Furthermore, different mRNA isoforms have different structures and opposing functions which can promote the progression of human diseases^3^. Importantly, the vulnerability of different brain anatomical regions and severity of pathology from different diseases can add to these complexities. Therefore, it is important to have a comprehensive profiling of the expression and splicing patterns that provide more information for different human CNS regions with different cell types in relation to neurological diseases.

Previous studies have added to the understanding of the transcriptomic architecture and patterns of the different regions and cell types of the CNS^4–7^. Region-specific changes were also apparent in a study which also revealed differences at the cellular level^4^. Although these studies added important insights into understanding connectivity and functional regulation in the human CNS, they are limited by the alternative splicing detection technology that was employed and limited sample size.

Further understanding of the human brain can be obtained by integrating genomic and transcriptomic data in the form of expression quantitative trait loci (eQTL) analyses. Past eQTL studies have been successful in exploring the effect of genetic control on transcriptional and splicing regulations in the human CNS and thereby gaining more insights into the underlying molecular mechanisms in the brain for different diseases pathways^6,8–11^. Tissue-specific eQTL signals at the transcript and exons levels were also reported and tissue-unique expression and splicing patterns were revealed^8,11,12^.

One known challenge eQTL studies face is insufficient sample size which is known to inaccurately estimate association strength^13^ leading to statistically invalid or nonsignificant results. Large sample sizes (*n* > 1000) have been used to decrease the likelihood of false positives^14–16^, however such large sample sizes are not feasible for studies using precious tissues such as human brains. Furthermore, large sample sizes can be very costly and computationally time-consuming^17^. Secondly, there are no comprehensive investigations of *cis*-acting eQTLs and *trans*-acting eQTLs patterns across different brain regions, and chromosomes at both the exon and transcript levels from the same individual.

In this study we used the United Kingdom Brain Expression Consortium (UKBEC) dataset, adding to the findings published previously by Trabzuni, *et al*.^18^ and Ramasamy, *et al*.^11^. In particular, we first evaluated the sufficiency of our sample size followed by an in-depth exploration of eQTL patterns. Specifically, we investigated characteristics of eQTLs found in single regions compared to multiple regions and how regions and chromosomes impact the number and effect sizes of *cis*-eQTL compared to *trans*-eQTL, at both transcript- and exon-levels.

The UKBEC dataset consists of 134 individuals in ten CNS regions including the cerebellum (CRBL), *n* = 130; frontal cortex (FCTX), *n* = 127; hippocampus (HIPP), *n* = 122; medulla (MEDU), *n* = 119; occipital cortex (OCTX), *n* = 129; putamen (PUTM), *n* = 129; substantia nigra (SNIG), *n* = 101; temporal cortex (TCTX), *n* = 119; thalamus (THAL), *n* = 124; and white matter (WHMT), *n* = 131; at both transcript and exon levels.

By using a set of simulations, the impact of sample size on the sensitivity and specificity to detect eQTL effect sizes (β) at a false discovery rate (FDR) threshold of 0.01 was evaluated for the following scenarios: single nucleotide polymorphisms (SNPs) in linkage equilibrium (LE), and in linkage disequilibrium (LD), genotyping errors (GE), lower expression level variance compared with residual variance (LV) and dominance (Dom). These five scenarios were chosen to represent situations likely to be encountered with real eQTL data. The results were used to ensure that the sample size of the UKBEC was sufficient.

Following this, genome-wide eQTL mapping was completed at transcript and exon levels and further classified as transcript-only, exon-only and “both”. We also investigated the patterns and effect sizes of eQTLs specific to one region (SR-eQTL) and those affect multiple regions (MR-eQTLs). A Shiny App was created to visualise these patterns at transcript and exon levels. We also add detailed information to our current understanding of *trans*-acting eQTLs, specifically how their effect sizes and numbers compare to those of *cis*-acting eQTLs in different chromosomes and brain regions.

## Results

### Sample size evaluation

#### Sample size evaluation using real UKBEC cerebellum (CRBL) data

**Figure 1:**
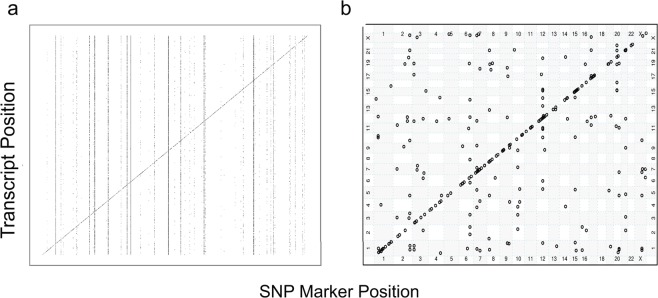
Genome-wide distribution of transcript-level eQTLs in CRBL. (**a**) Simulated eQTL data in linkage equilibrium for a single simulation and (**b**) observed eQTL data. Each point in both plots represents an eQTL between (the SNP on different chromosomes on the *x*-axis) and (the transcript on different chromosomes on the *y*-axis). The clear diagonal band represents *cis*-acting eQTLs while the off-diagonal points represent *trans*-acting eQTLs (defined as eQTL with a difference between the SNP and transcript site of at least 3.16 Mb). There are many more points in the simulated data plot (**a**) as these are the simulated “true” eQTLs, many of which are not detected in the subsequent eQTL mapping analysis. As an example, 2.19% of “true” eQTLs were detected across all simulations.

The total number of significant eQTLs detected with 130 CRBL samples was 1,956 before redundant eQTLs were removed because of SNPs in linkage disequilibrium (LD). The number of eQTLs detected when the sample size was 100 was comparable when the sample size was 130 (Table 1). However, when the sample size was reduced to 50 and lower, the number of detected eQTLs became extremely variable. For example, when *n* = 25, one run detected 229,813 eQTLs. These results indicated that sample sizes lower than 100 can produce many false positives associations. Following the results outlined in Table 1, a more detailed evaluation was undertaken with simulated data. Figure 1a shows a plot of the simulated eQTLs in CRBL with a clear diagonal band representing the presence of *cis*-acting eQTLs and with some SNPs having a large number of *trans*-acting eQTLs compared to other SNPs indicated by the vertical bands. This pattern of detected eQTLs has been seen in previous empirical studies^19^ as well as in the genome-wide distribution of detected eQTLs in the real CRBL data (see Fig. 1b).

**Table 1:** Number of eQTLs detected from 10 replications (Rep.) of 100, 50, 25 and 13 brains in cerebellum (CRBL) region.

No. of Brains	All	Rep. 1	Rep. 2	Rep. 3	Rep. 4	Rep. 5	Rep. 6	Rep. 7	Rep. 8	Rep. 9	Rep. 10	Median
**130**	1956											
**100**		1058	1075	1082	1088	1103	1116	1174	1178	1279	1341	1110
**50**		858	1561	2088	2470	2621	3661	4510	4773	4937	6574	3141
**25**		1535	2319	5594	6991	7418	28381	36834	89053	199909	229813	17900
**13**		0	0	0	0	0	3618	3791	3822	3852	7383	1809

Ten replications of *n* = 100, 50, 25, 13 were randomly selected from the 130 available CRBL samples and used to detect eQTLs at the transcript-level. The number of eQTLs detected are sorted from smallest to largest from replications 1 to 10. When *n* = 100, the number of eQTLs detected per sample was comparable to the 1,956 eQTLs detected with the total number of available CRBL samples (*n* = 130) given the decrease in sample size. However, when *n* ≤ 50, the number of eQTLs detected became extremely variable across samples with implausibly high numbers of eQTLs (e.g. sample 10, *n* = 25, the number of eQTLs detected = 229,813).

**Figure 2:**
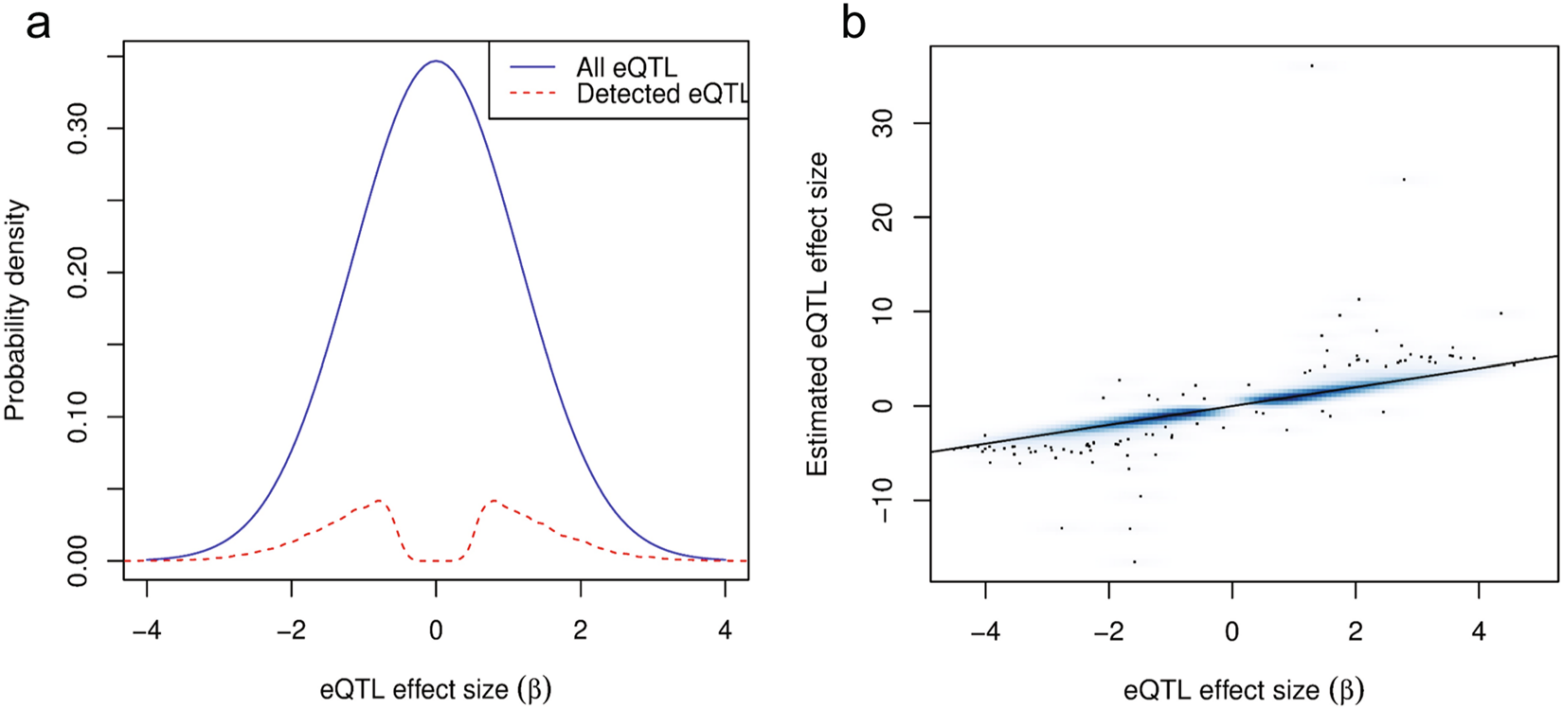
Distribution of simulated eQTL effect sizes. (**a**) The distribution of effect sizes of all eQTLs (in blue) compared to the distribution of effect sizes of detected eQTLs (in red). These effect sizes are based on the simulations of the scenario with SNPs in linkage equilibrium (LE) with a false discovery rate (FDR) threshold of 0.01 and *n*_sample_ = 150. At larger eQTL effect sizes (|β| ≥ 1), eQTLs are more likely to be detected but it is clear that many eQTLs are not being detected (97.8% in across all simulations). There is a dip in the distribution of detected eQTL effect sizes around β = 0 due to the stringency of the FDR threshold (≤0.01). (**b**) A smoothed scatter plot of estimated versus actual eQTL effect sizes, for those that were detected. Like (**a**), these effect sizes are from the simulations of the LE scenario with a FDR threshold of 0.01 and *n* = 150. Overall, the estimated effect sizes of eQTLs when they are detected are accurate, though there are a few outliers.

Moreover, in Fig. 2a, we compared the distribution of the effect sizes of the eQTLs that were detected by MatrixEQTL to the distribution of the effect sizes of all simulated eQTLs. It is clear from this figure that many eQTLs are not being detected, for example, across all simulations, 97.8% of eQTLs were not detected. This is particularly true for eQTLs with smaller effect sizes, |β| ≤ 1. However, when they are detected, their estimates are generally accurate (see Fig. 2b). Although these plots are from a single scenario (*n* = 150, FDR = 0.01, SNPs under linkage equilibrium (LE), this pattern was seen across all scenarios.

#### Investigating sensitivity and specificity using simulated data for various genetic models

**Figure 3:**
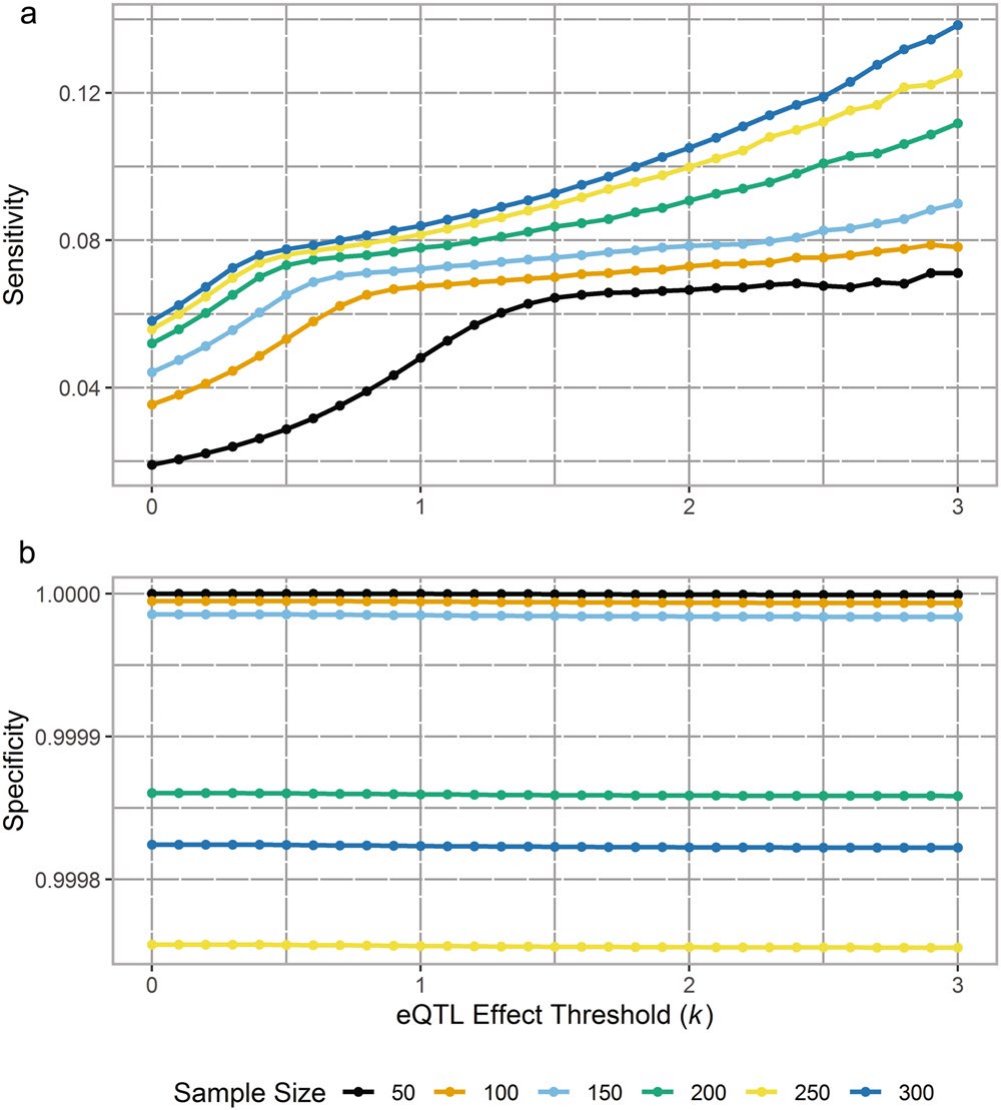
The sensitivity (**a**) and specificity (**b**) to detect eQTLs at a range of effect size thresholds for the scenario with linkage equilibrium (LE). (**a**) The plot shows the average sensitivity from 100 simulations in each sample size (*n* = 50, 100, 150, 200, 250, 300) to detect a range of eQTL effect size thresholds, *k* (0–3 in 0.1 increments). As both sample size and effect size threshold increase, the sensitivity to detect eQTLs increases. However, sensitivity is very low across all sample sizes and effect size thresholds, with the greatest level of sensitivity (i.e. when *n* = 300, *k* = 3) being less than 0.14. At lower effect size thresholds, sensitivity to detect eQTLs across sample sizes (except *n* = 50) is comparable. (**b**) The plot shows the average specificity from 100 simulations in each sample size (*n* = 50, 100, 150, 200, 250, 300) to detect a range of eQTL effect size thresholds*, k* (0–3 in 0.1 increments). As sample size increases, the specificity to detect eQTLs decreases across *k* with largest decrease of 2.5 × 10^−4^ at *k* = 3 when *n* increases from 50 to 250. However, specificity is very high across all sample sizes and effect size thresholds with the lowest level of specificity (i.e. when *n* = 250, *k* = 3) being greater than 0.9997. For each sample size, there is virtually no discernible decrease in specificity as the effect size threshold increase

To investigate the adequacy of the UKBEC dataset sample size, the sensitivity and specificity to detect eQTLs of a specified effect size threshold were evaluated. More specifically, simulated eQTLs were explored where the absolute value of the true effect sizes of the eQTLs were greater than or equal to a threshold (i.e. if |β| ≥ *k*, where *k* is the threshold value). As expected, an increase in sample size led to an increase in sensitivity to detect eQTLs across a range of thresholds, 0 ≤ *k* ≤ 3 (see Fig. 3a). However, the sensitivity to detect eQTLs was generally low with only 2–14% of eQTLs being detected across the range of eQTL effect sizes and sample sizes. Figure 3a also shows that when sample size increased from 50 to 100, there was a large increase in sensitivity with an additional 1.1%–2.7% of eQTLs detected for smaller thresholds (0 ≤ *k* ≤ 1.3), but less of an improvement for larger effect size thresholds (*k* ≥ 1.4). However, there was a considerable improvement in sensitivity of 1% (from 0.012 when *k* = 2 to 0.022 when *k* = 3) when *n* increased from 150 to 200. These observations were drawn from the scenario with FDR = 0.01 and LE, however, similar patterns were seen in the other scenarios (Supplementary Figs. S1–4).

In general, specificity was extremely high with relatively few false positives. For example, for the LE scenario at *n* ranging from 50 to 150, specificity was very close to 1, indicating that nearly 100% of true non-eQTLs (i.e. SNP-transcript pairs with no real association) were correctly classified. There was a marginal decrease in specificity with increasing sample size, with the maximum difference being 2.47 × 10^−4^ between *n* = 250 and *n* = 50 (see Fig. 3b). Unexpectedly, the largest sample size evaluated, *n* = 300 showed slightly larger specificity than for *n* = 250 across all *k* (see Fig. 3b). This non-monotonic change in specificity with increasing sample size was not due to an insufficient number of simulations (100), as the maximum standard errors of specificity were 1.3 × 10^−6^ for *n* = 250 and 9.9 × 10^−7^ for *n* = 300. Interestingly, this pattern was found in all scenarios except for LD where specificity was higher when *n* = 250 than when *n* = 200 or *n* = 300 (Supplementary Figs. S5–8). Both the mean sensitivity and specificity curve was relatively smooth over the range of *k*, indicating that 100 simulations were enough (Supplementary Fig. S9).

**Figure 4:**
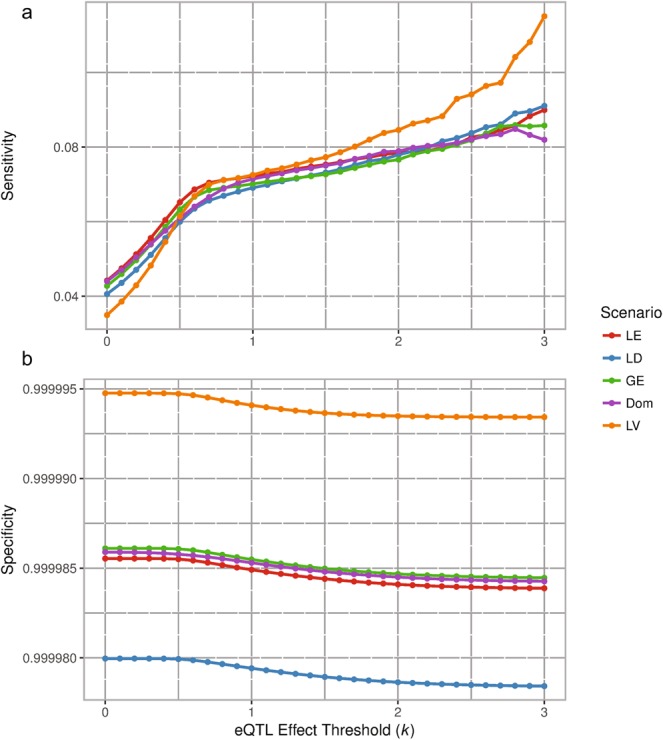
Average sensitivity (**a**) and specificity (**b**) of 100 simulations to detect eQTL at a range of effect size thresholds (*k*) for five scenarios. The five scenarios were: SNPs in linkage equilibrium (LE) and disequilibrium (LD), genotyping errors (GE), lower expression level variance compared with residual variance (LV) and dominance (Dom) when *n* = 150. The sensitivity of each scenario increased with increasing effect size thresholds, however remained low overall with the highest level of sensitivity (LV scenario, *k* = 3) being lower than 0.12. Four scenarios (LE, LD, GE, and Dom) show comparable sensitivity levels across the effect size thresholds while LV showed a deviation from a threshold of 1.5 ≤ *k* ≤ 3. Specificity slightly decreases with increasing effect size thresholds for all scenarios, with LV showing the highest specificity and LD the lowest. However, specificity is high for all scenarios in the range of 0.9999875 to 0.999995.

The sensitivity and specificity from the simplest, “base” scenario (LE) was then used to draw comparisons between the other scenarios (LD, GE, LV, Dom as defined earlier) at *n* = 150 (see Fig. 4). The sample size of 150 was chosen for these comparisons as it is the closest to the sample size of the UKBEC.

For the GE and Dom scenarios, the sensitivity to detect eQTLs was only slightly lower than LE at high threshold values. For example, at *k* = 3, the sensitivity for GE was 2.74 × 10^−3^ lower. The LD scenario had lower sensitivity than LE at the lower threshold until *k* ≥ 2.1, where it increased and stayed stable. With the LV scenario, sensitivity at the lower thresholds (0 ≤ *k* ≤ 1) was noticeably smaller than LE but continued to increase and was discernibly greater when *k* ≥ 1.5 with the largest difference of 2.51% greater when *k* = 3.0.

The specificity of the LD scenario had a discernibly lower specificity than the LE scenario by 5.5 × 10^−6^, whereas the LV scenario was noticeably higher by 9.3 × 10^−6^. Scenarios GE and Dom had comparable specificity to the LE scenario. However, it needs to be noted that the specificity for all scenarios was close to 1 for all *k*.

#### FDR thresholds evaluation

To illustrate the effect of different FDR thresholds on the sensitivity and specificity; the sample size of *n* = 150 when *k* = 2 for the scenario with SNPs in LE was evaluated. The average number of false negative (FN) and false positive (FP) associations for 100 simulations were calculated along with the sensitivity and specificity for three thresholds of FDR = 0.01, 0.05 and 0.10 (Table 2). Results indicated that as the FDR threshold was made less stringent (0.01 to 0.10), sensitivity increased by 0.5% while specificity decreased by 0.002%. More importantly, the numbers of FP eQTLs showed a marked increase as FDR became less stringent (from 6314 to 13734). More importantly, a less stringent FDR threshold (0.01 to 0.10) resulted in a modest increase in the number of eQTL detected (+6) but an overwhelmingly large increase in the number of the FP eQTLs (+7420).

**Table 2:** Sensitivity and specificity of eQTL detection with different false discovery rates (FDR) threshold.

FDR	Sensitivity	Specificity	FN	FP
0.01	0.0808	0.9999842	1275	6314
0.05	0.0841	0.9999737	1271	10530
0.10	0.0859	0.9999657	1269	13734

This table shows the number of False Negative (FN) and False Positive (FP) eQTL associations at different FDR thresholds when *n* = 150 and *k* = 2. There is a huge increase of the FP eQTLs as the FDR cut-off is made less stringent, but only a modest decrease for the FN associations (+6).

### Genome-wide eQTLs

An eQTL is defined as an association between transcript or exon expression level and a SNP. However, due to LD, redundant SNPs were removed and haplotype blocks were used to represent SNPs in LD, *R*^2^ > 0.5. We have systematically defined several classifications of eQTLs. We defined an eQTL as being a multi-region eQTL (MR-eQTL) if the eQTL was present in more than one region, and a single-region eQTL (SR-eQTL) when an eQTL was found in one region only. For example, if an eQTL was found in CRBL and FCTX, it represents two eQTLs but only one MR-eQTL. In addition, we investigated transcript-level and exon-level eQTLs, i.e. associations between an exon and a haplotype, or a transcript and a haplotype, respectively. An exon-level eQTL was classified as exon-only eQTL when one or more of the exons within a transcript cluster were significantly associated with a haplotype, but without a corresponding association at the transcript-level (to that same haplotype). While, the transcript-level eQTL was classified as transcript-only when none of the exons within the same transcript cluster occurred as eQTLs with the same haplotype at the exon-level. For eQTLs found at the transcript-level and at the exon-level (for exons within the transcript cluster) to the same haplotype, these eQTLs were classified as “both”. For example, an MR-eQTL can be classified as “both” but can be a transcript-only SR-eQTL. This is important to consider when interpreting results, as the ambiguity of classifying eQTLs as “both” (which includes transcript-level and exon-level eQTLs) limits our understanding of whether genomic regulations happen through transcript or exon level mechanisms.

#### Region-by-region eQTLs

Genome-wide eQTL mapping across the ten regions resulted in a total of 1,096 transcript-level eQTLs and 7,009 exon-level eQTLs (only the most significant eQTL association in each haplotype was retained). Table 3 summarises these eQTLs detected for the different classifications described above, as well as *cis*- or *trans-*acting. It is worth noting that CRBL has more *trans*-acting eQTLs compared with other regions (132 out of 209 total). There were two main issues that prevented us from continuing the analyses using these three classifications: transcript-only, exon-only, both. Firstly, the number of transcript-only eQTLs available for analysis was greatly reduced (47.5% of total transcript-level eQTLs). Secondly, there was ambiguity with the eQTLs classified as “both”. For example, there were some transcript clusters where all the exons were found to be associated with eQTLs but was not an eQTL at the transcript-level. In Table 3, we considered these exon-level eQTLs as “exon-only”, but it is also reasonable for the corresponding transcript to be regarded as an eQTL. Currently, there is no consensus on such issues. Therefore, for the remainder of the paper, all analyses were undertaken either at the transcript-level eQTLs (i.e. includes transcript-only and a subset of “both”) or at the exon-level eQTLs (i.e. includes exon-only and a subset of “both”).

**Table 3:** Number of eQTLs detected in a genome-wide scan.

Region	Transcript-only			Exon-only			Both		
	*cis*	*trans*	total	*cis*	*trans*	Total	*cis*	*trans*	total
CRBL	77	132	209	1479	578	2057	390	64	454
FCTX	50	12	62	519	441	960	126	26	152
HIPP	46	30	76	522	518	1040	94	28	122
MEDU	36	24	60	424	161	585	84	14	98
OCTX	37	32	69	377	267	644	100	10	110
PUTM	29	26	55	166	353	519	42	20	62
SNIG	27	36	63	88	110	198	22	20	42
TCTX	71	47	118	530	398	928	136	14	150
THAL	31	36	67	280	188	468	62	20	82
WHMT	42	28	70	1091	262	1353	234	18	252
Total	446	403	849	5476	3276	8752	1290	234	1524

The number of eQTLs are grouped into transcript-only, exon-only and both and further classified by *cis*-acting and *trans*-acting. “Transcript-only” are eQTLs found only at the transcript-level. “Exon-only” are eQTLs found only at the exon-level. “Both” are eQTLs found at both the transcript-level and at the exon-level within the transcript. Due to these definitions, the numbers under the “Both” column include both transcript-level and exon-level eQTLs. There may also be overlapping numbers of eQTLs between the regions as some eQTLs may have been detected in several regions.

**Figure 5:**
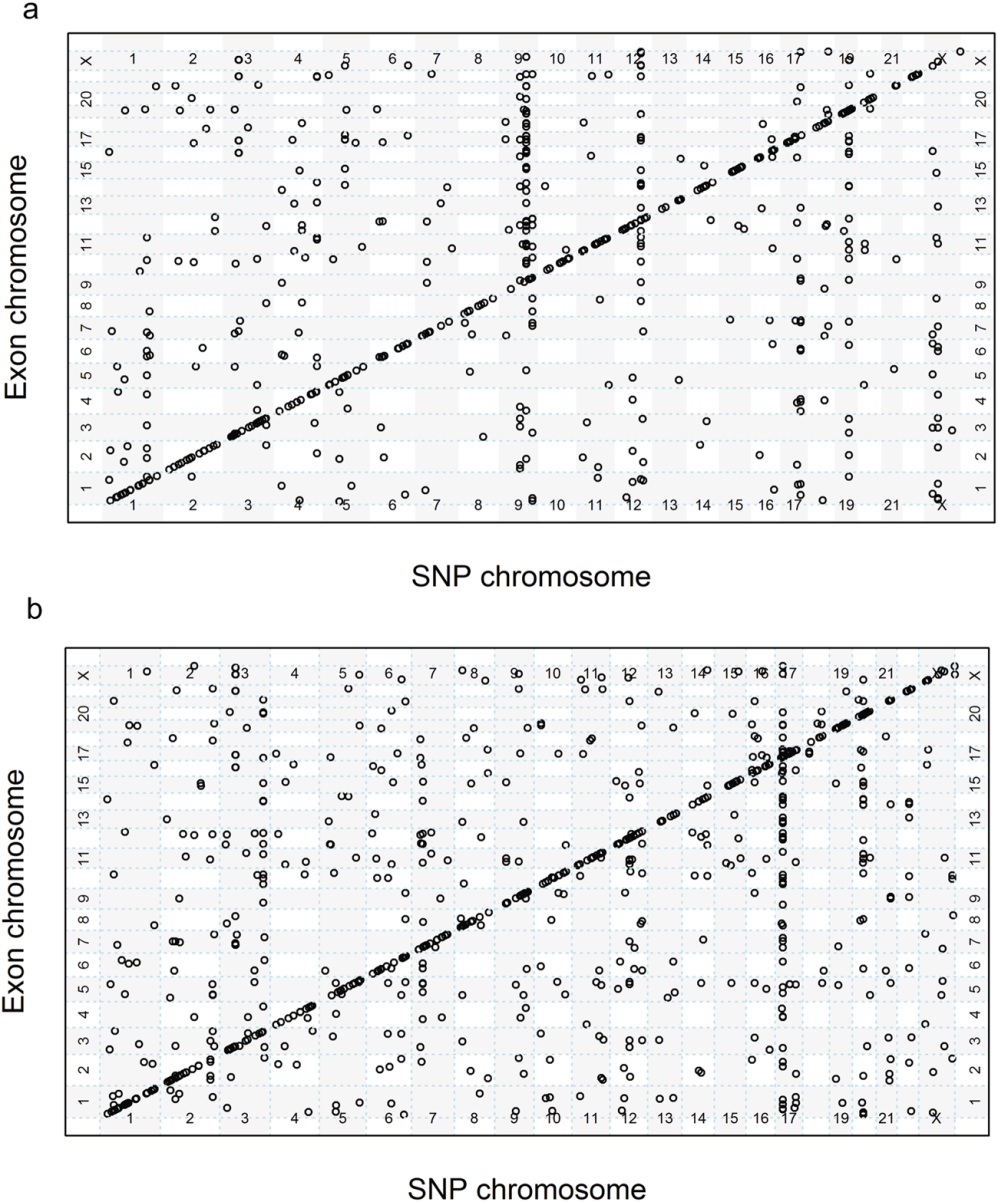
Genome-wide distribution of exon-level eQTLs for the (**a**) TCTX and (**b**) CRBL. Each point in both plots represents an exon-level eQTL with the SNP chromosomes on the *x*-axis and the transcript chromosome on the *y*-axis. Note that these are the eQTLs after redundant SNPs were removed (i.e. these eQTLs are associated with haplotypes representing a block of SNPs in linkage disequilibrium). The clear diagonal line represents *cis*-acting eQTLs while the off-diagonal points represent *trans*-acting eQTLs. There are vertical lines of eQTLs points indicating that there are haplotypes or adjacent haplotypes that are associated with multiple exons. Less evident are horizontal lines of eQTL points, though more apparent in the temporal cortex (**a**) than the cerebellum (**b**). The eQTL points along these horizontal lines suggest that there are exons associated with many haplotypes.

From Fig. 1 which shows map locations for CRBL transcript-level eQTLs, *cis*-acting eQTLs clustered along the diagonal whilst the off-diagonal points represent *trans*-acting eQTLs. Vertical lines of eQTLs are particularly evident at the exon-level for TCTX and CRBL (see Fig. 5 and Supplementary Figs. S10–18 for the other nine regions). Table 4 demonstrates the frequency distribution for eQTLs per gene bin (1 gene, 2–5 genes, 6–10 genes, >10 genes) to highlight how one haplotype can be associated with multiple transcripts/exons. In particular, there is one haplotype associated with 187 exons in the PUTM, namely rs13045538 on Chr 20 (bolded in Table 4).

**Table 4:** Frequency distribution of eQTLs associated with one or more transcripts/exons.

Gene bins	Transcript level					Exon level				
	1	2-5	6–10	>10	Max	1	2–5	6–10	>10	Max
CRBL	297	34	6	1	11	658	217	68	20	60
FCTX	119	4	0	1	11	313	103	9	8	129
HIPP	100	7	1	1	14	295	108	17	7	115
MEDU	92	8	0	0	3	269	76	12	3	37
OCTX	104	3	1	0	8	297	81	12	5	41
PUTM	61	2	0	1	20	180	31	2	3	**187**
SNIG	55	8	1	0	7	93	23	2	2	26
TCTX	156	15	0	0	5	358	117	18	11	34
THAL	96	6	0	0	2	261	62	5	2	25
WHMT	161	8	2	0	9	428	196	36	12	42

This table shows the association of a haplotype with one or more transcripts/exons as specified by bin categories. Genes were grouped into four bins: 1, 2–5, 6–10, >10 and grouped into transcript-level and exon-level for each region of the brain. For example, CRBL at transcript level, on one hand has one haplotype associated with 11 transcripts, i.e. we have 11 eQTLs. On the other hand, there are 34 haplotypes, each associated with between two and five transcripts i.e. between two and five eQTLs per haplotype. The “Max” gene bin is the maximum number of genes (transcript/exon) associated with a single haplotype.

What is not so apparent from Figs. 1 and 5 is that expression traits at both transcript- and exon-levels may be associated with multiple haplotypes, i.e. horizontal lines in figures. Table 5 shows the frequency distributions of eQTLs for the association of transcript/exon per haplotype block bin (1 haplotype, 2–5 haplotypes, 6–10 haplotypes, >10 haplotypes). In particular, there are 337 haplotypes in the CRBL associated with one exon (bolded).

**Table 5:** Frequency distributions of eQTLs associated with one or more haplotypes.

Haplo bins	Transcript level					Exon level				
	1	2–5	6–10	>10	Max	1	2–5	6–10	>10	Max
CRBL	206	56	5	4	18	534	160	31	33	**337**
FCTX	65	22	2	0	9	403	128	15	8	53
HIPP	75	14	1	1	11	460	113	8	14	48
MEDU	57	20	0	0	5	188	53	19	7	67
OCTX	76	14	1	0	7	293	74	12	7	32
PUTM	54	9	1	0	7	234	91	8	1	13
SNIG	62	9	0	0	3	128	25	1	1	16
TCTX	103	22	4	0	9	432	106	19	6	33
THAL	73	13	1	0	8	248	63	8	2	14
WHMT	103	23	4	0	10	296	97	24	24	136

This table shows the association of a transcript/exon with one or more haplotypes as specified by bin categories. Haplotypes were grouped into four bins: 1, 2–5, 6–10, >10 and grouped into transcript-level and exon-level for each region of the brain. The “Max” haplotype bin is the maximum number of haplotypes associated with a single transcript/exon. For example, at the transcript-level, there is only one eQTL detected in the putamen (PUTM) associated with between six to 10 haplotypes, the “Max” column would indicate that the exact number would be seven haplotypes. At the exon-level, there are 33 exons in the cerebellum (CRBL) that are associated with >10 haplotypes, where one of these 33 exons is associated with 337 haplotypes (from the “Max” column).

#### Single-region eQTLs

Table 6 shows the numbers of SR-eQTLs across the ten regions at transcript and exon levels and also *cis*- versus *trans*-acting. It is evident that CRBL has the most transcript-level SR-eQTLs with 347 followed by the WHMT with 116. In general, the vast majority of transcript-level SR-eQTLs were *trans*-acting.

**Table 6:** Number of eQTLs detected in a single-region only (SR-eQTLs).

Region	Transcript-level			Exon-level		
	*cis*	*trans*	Total	*cis*	*trans*	total
CRBL	185	**162**	347	1237	574	1811
FCTX	25	22	47	176	422	598
HIPP	10	41	51	158	471	629
MEDU	17	29	46	127	114	241
OCTX	9	35	44	88	228	316
PUTM	10	35	45	34	347	381
SNIG	6	46	52	7	98	105
TCTX	26	53	79	182	370	552
THAL	15	45	60	67	160	227
WHMT	80	36	116	849	216	1065
Total	383	504	887	2925	3000	5925

This table shows the number of single-region eQTL (SR-eQTL) mapped in each of the ten regions. At the transcript and exon levels, as well as cis versus trans. It is worth noting that the largest number of SR-eQTLs is in CRBL and it is clear that that the majority of single-region eQTLs are trans-acting (e.g. 504/887 = 56.82% trans-acting eQTLs at the transcript-level).

#### Multi-region eQTLs

The results from the region-by-region and single-region viewpoints suggested that there are different patterns of the eQTLs across the ten regions at transcript and exon levels. This led us to study eQTLs that were detected to have an effect across multiple regions of the brain, i.e. MR-eQTLs.

**Figure 6:**
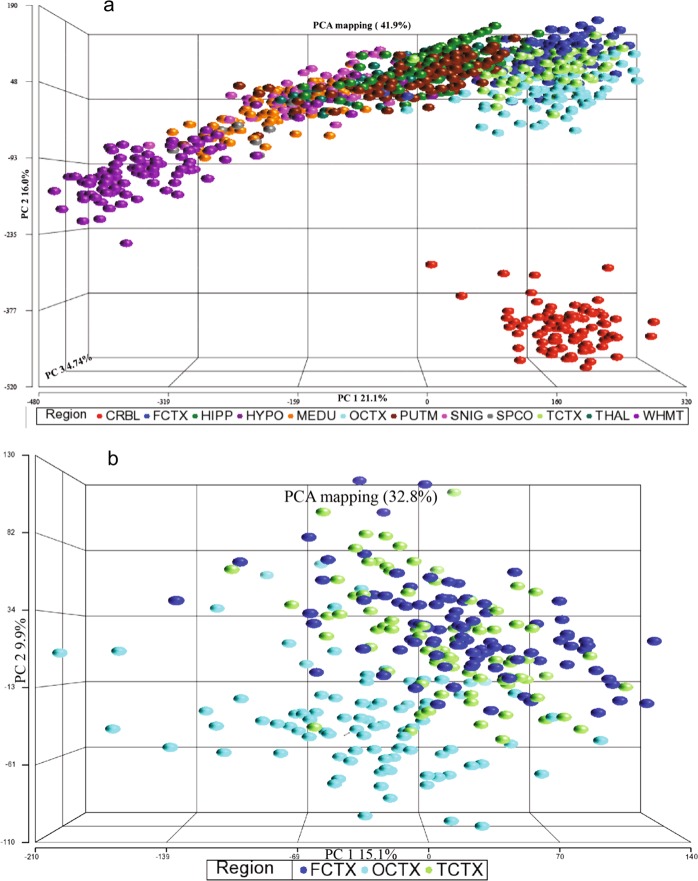
First three principal component scores of (**a**) all CNS-regions and (**b**) three cortex regions. These are 3D scatterplots of the first three PCs from a PCA on the expression data of (**a**) 12 brain regions (cerebellum, frontal cortex, hippocampus, hypothalamus, medulla, occipital cortex, putamen, substantia nigra, spinal cord, temporal cortex, thalamus, and white matter) and (**b**) the three cortices (frontal, occipital and temporal). Each point represents a single brain sample from a specific region with regions colour coded to demonstrate regional differences in expression values. Looking at all regions, the first three components explain 41.9% of the variability in expression values across all transcripts. The cerebellum region (CRBL; red spheres) cluster separately from the other 11 regions indicating that the expression pattern in this region is unique. In addition, white matter (WHMT; purple spheres) also shows a separate cluster demonstrating different expression patterns than other regions. Looking at the cortex regions, the first three components explain 32.8% of the variability in expression values across all transcripts. The occipital cortex region (OCTX; light blue spheres) is clustering separately from the frontal cortex (FCTX; dark blue spheres) and the temporal cortex regions (TCTX; light green spheres), indicating that the expression pattern is more distinct in this region.

Looking at eQTL patterns, there were four transcript-level eQTLs and eleven exon-level eQTLs that were mapped in all ten brain regions (Table 7). It is also evident that eQTLs tended to cluster in certain regions, as shown in Table 8 (transcript level) and Table 9 (exon level). Generally, the FCTX showed the greatest sharing of eQTLs at the transcript-level (66%) (Table 8) while the MEDU had the greatest at the exon-level (62%) (Table 9). The three cortical regions (FCTX, OCTX, and TCTX) have many eQTLs in common where for each of these regions, the highest number shared were with the other two cortical regions. This reflects the clustering seen in the Principal component analysis (PCA) that was used to explore the different patterns of gene expression across the ten brain regions (see Fig. 6b) where the cortical regions had similar expression profiles. Interestingly, there was a slight separation of OCTX from the other two cortex regions in the PCA. Furthermore, it is clear from Fig. 6a, that CRBL clustered separately from the other regions suggesting a distinctive expression pattern. As alluded to in Table 6 and Fig. 6a, eQTLs found in CRBL showed high region specificity (transcript: 20% shared, exon: 21% shared).

**Table 7:** Frequency distributions of eQTLs and multi-region eQTLs (MR-eQTLs) for the number of regions.

No. Regions	Transcript level		Exon level	
	Frequency (MR-eQTLs)	Cumulative Frequency (eQTLs)	Frequency (MR-eQTLs)	Cumulative Frequency (eQTLs)
1	*887	887	*5925	5925
2	87	174	523	1046
3	50	150	216	648
4	29	116	130	520
5	15	75	77	385
6	12	72	59	354
7	4	28	30	210
8	3	24	26	208
9	5	45	12	108
10	4	40	11	110
Total	1096	1611	7009	9514

This table includes the number of eQTLs (i.e. frequency for single-region, SR-eQTL) and the number of multi-region eQTLs (MR-eQTLs) (i.e. frequency for more than one brain region) grouped by transcript-level and exon-level. For example, at the transcript-level, there are four MR-eQTLs detected in all ten brain regions which adds to a total of 40 eQTLs (i.e. the cumulative frequency). At the exon-level, there are 77 MR-eQTLs detected in five brain regions, adding to 385 eQTLs. In total, there are 1,096 SR/MR-eQTLs and 1,611 eQTLs at the transcript-level. Furthermore, there were 7,009 SR/MR-eQTLs and 9,514 eQTLs at the exon-level. *The 887 and 5925 are the SR-eQTLs from the transcript and exon level respectively.

**Table 8:** Regional distribution of transcript-level multi-region eQTLs (MR-eQTLs).

	CRBL	FCTX	HIPP	MEDU	OCTX	PUTM	SNIG	TCTX	THAL	WHMT
CRBL										
FCTX	37									
HIPP	34	41								
MEDU	25	20	36							
OCTX	46	**45**	30	20						
PUTM	15	26	28	14	21					
SNIG	14	17	18	17	16	13				
TCTX	49	62	54	27	53	28	17			
THAL	19	27	32	21	26	20	18	34		
WHMT	28	30	36	34	18	18	14	34	29	
MR	**89**	91	86	63	80	41	32	114	48	80
Total	**436**	138	137	109	124	86	84	193	108	196
Percent	20	66	63	58	65	48	38	59	44	41

The MR-eQTLs used in this table are all the eQTLs found at the transcript-level (i.e. includes transcript-only and subset of “both”). The entries below the diagonal are the number of MR-eQTLs in common between those two regions. ‘Total’ is the total number of eQTLs detected in that brain region while ‘MR’ is the number of MR-eQTLs in that region that are also detected in other brain regions. For example, in the cerebellum (CRBL), 436 eQTLs were found at the transcript-level, of these, 89 were also detected in another region (i.e. 20%). Note that the number of MR-eQTLs shared between a region and another do not add up to the total number of MR-eQTLs for that particular region as there are MR-eQTLs that were detected in more than two regions. For example, there are 45 MR-eQTLs shared between the frontal (FCTX) and occipital (OCTX) cortices and some of these are also shared with the temporal cortex (TCTX) as well (i.e. entries can overlap).

**Table 9:** Regional distribution of exon-level multi-region eQTLs (MR-eQTLs).

	CRBL	FCTX	HIPP	MEDU	OCTX	PUTM	SNIG	TCTX	THAL	WHMT
CRBL										
FCTX	227									
HIPP	188	202								
MEDU	112	111	226							
OCTX	180	**201**	192	137						
PUTM	76	95	97	67	107					
SNIG	34	51	77	85	58	38				
TCTX	197	223	202	143	208	101	55			
THAL	99	124	154	138	139	78	68	156		
WHMT	167	131	168	192	139	91	51	167	139	
MR	**473**	438	472	393	383	169	114	451	282	414
Total	**2284**	1036	1101	634	699	550	219	1003	509	1479
Percent	21	42	43	62	55	31	52	45	55	28

The MR-eQTLs used in this table are all the eQTLs found at the exon-level (i.e. includes exon-only and subset of “both”). The entries below the diagonal are the number of MR-eQTLs in common between those two regions. ‘Total’ is the total number of eQTLs detected in that brain region while ‘MR’ is the number of MR-eQTLs in that region that are also detected in other brain regions. For example, in the cerebellum (CRBL), 2284 eQTLs were found at the exon-level, of these, 473 were also detected in another region (i.e. 21%). Note that the number of MR-eQTLs shared between a region and another do not add up to the total number of MR-eQTLs for that particular region as there are MR-eQTLs that were detected in more than two regions. For example, there are 201 MR-eQTLs shared between the frontal (FCTX) and occipital (OCTX) cortices and some of these are also shared with the temporal cortex (TCTX) as well (i.e. entries can overlap).

#### 3D MR-eQTLs visualisation (Shiny app)

**Figure 7:**
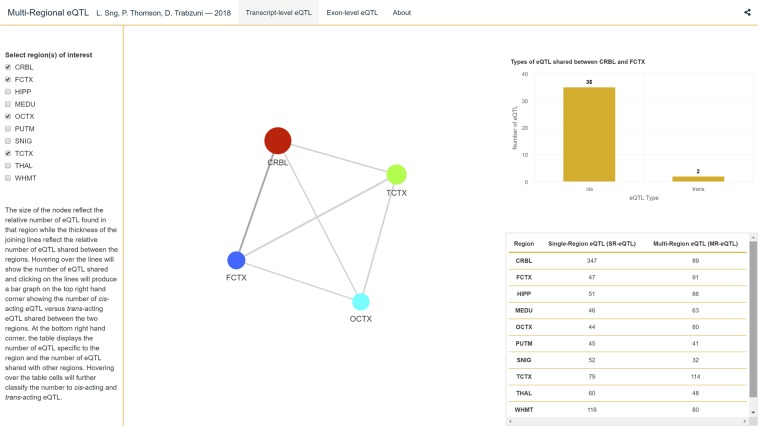
A screen capture of the Shiny App. In this app, users can visualise the MR-eQTL patterns between specific regions of interest. The diameter of each sphere is proportional to the number of eQTLs associated with the region of the brain. The width of the line connecting a pair of regions is proportional to the number of eQTLs in common between those two regions with the actual number presented for the *cis*/*trans*-acting eQTLs. There are two pages, one for transcript-level and another for exon-level. The app can be found at https://lmf-sng.shinyapps.io/Multi-Regional_eQTL/.

To visualise these MR-eQTLs across all regions, a ‘Shiny app’ has been created for the transcript-level and exon-level (see Fig. 7). Using this dynamic app (https://lmf-sng.shinyapps.io/Multi-Regional_eQTL/), regions can be specified and the number of the shared eQTLs between these different regions are displayed for both *cis*- and *trans*-acting eQTLs. A table summary including the numbers of SR-eQTLs at transcript and exon levels is also incorporated.

#### Effect sizes of multi-region eQTLs

**Figure 8:**
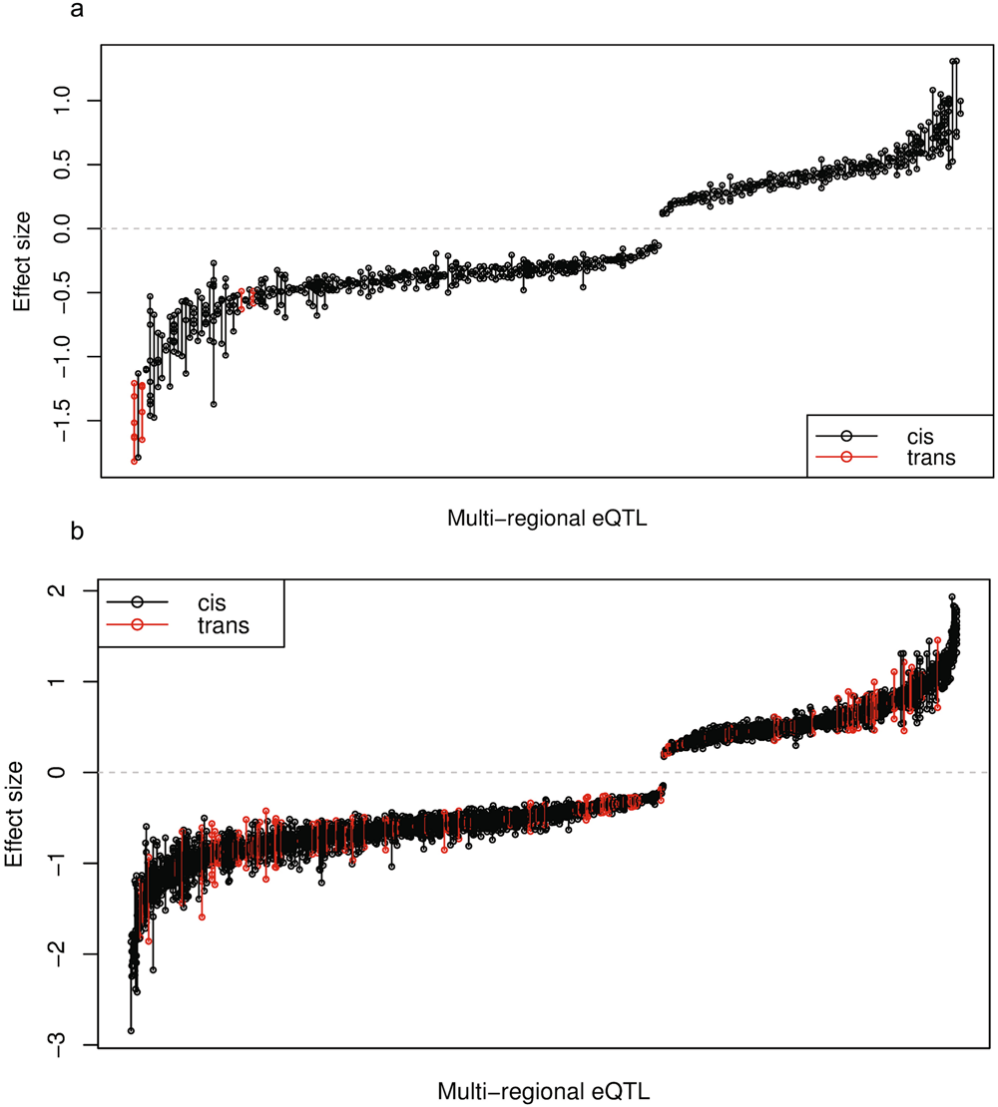
Estimated effects sizes of (**a**) transcript-level and (**b**) exon-level multi-region eQTLs. These are the regression coefficients of the MR-eQTLs found at the transcript-level and at the exon-level. Each line is an MR-eQTL with the points representing the effect size for a brain region. These are sorted from the lowest to the highest mean eQTL effect size with *cis*-acting MR-eQTLs in black and *trans*-acting MR-eQTLs in red. The plots also highlight the much smaller proportion of *trans*-acting MR-eQTLs in both transcript- and exon-levels.

The effect sizes of MR-eQTLs at both transcript and exon-level across regions were mapped (see Fig. 8). Results suggested that when an eQTL is active in multiple regions, it affects expression levels in a similar way. It is also worth noting that some MR-eQTLs clustered in different regions between the transcript-level and exon-level. For example, at the transcript-level, the eQTLs associated with FLYWCH-type zinc finger 1 (*FLYWCH1*) were significant in the CRBL, MEDU and WHMT while at the exon-level they were mapped in the CRBL, TCTX and WHMT. Figure 8 also highlights the relatively small number of *trans*-acting MR-eQTLs: 1.9% (4) at the transcript level and 10.6% (115) at the exon level.

**Figure 9:**
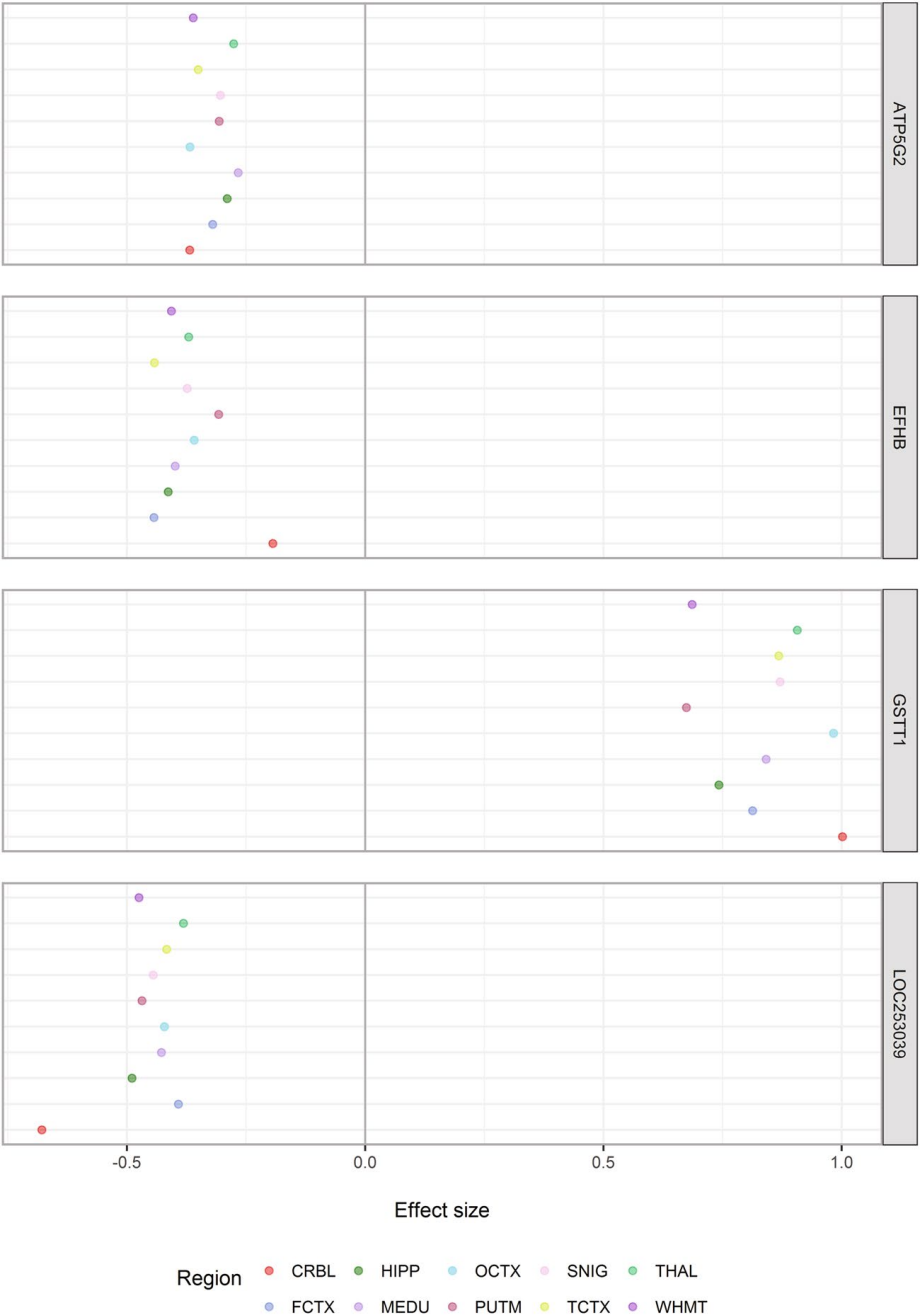
Estimated effect sizes for transcript-level eQTLs detected in all 10 brain regions. Each point represents the estimated effect size (regression coefficient) of the transcript-level eQTLs detected in a specific brain region. Regions are colour coded. These are the only four transcript-level eQTLs detected in all ten brain regions. From these plots, MR-eQTLs in general have similar effect sizes across the brain regions. The effect sizes for the MR-eQTLs corresponding to genes EFHB and LOC253039 in the cerebellum (CRBL) seems to diverge from the effect sizes in the other nine regions.

Figure 9 shows the estimated eQTL effect sizes for the four transcript-level eQTLs which were detected in all 10 brain regions. This further highlights the observation that MR-eQTLs have similar effect sizes in regions they are present in. Also, in the two cases where the MR-eQTLs (i.e. for genes Ef-hand domain family member B (*EFHB*) and *LOC253039*) had an effect size that deviates from the rest, they were detected in the CRBL. This points to CRBL having a separate eQTL pattern as aforementioned.

### Regional and chromosomal differences between *cis*- and *trans*-acting eQTLs

Observations from Table 6 motivate us to explore patterns of *cis*- and *trans*-acting eQTLs more systematically. Specifically, there were differing ratios of *cis*-acting versus *trans*-acting SR-eQTLs across different regions (e.g. CRBL, FCTX, and WHMT showed less *trans*-acting SR-eQTLs compared to *cis*-acting, but the other regions showed the opposite). Furthermore, as part of the genome-wide mapping approach, we were interested to assess if some chromosomes had relatively different patterns compared with other chromosomes (e.g. more or less *cis*- versus *trans*-eQTL between chromosomes).

#### Percentage of *cis*- versus *trans*-acting eQTLs

**Figure 10:**
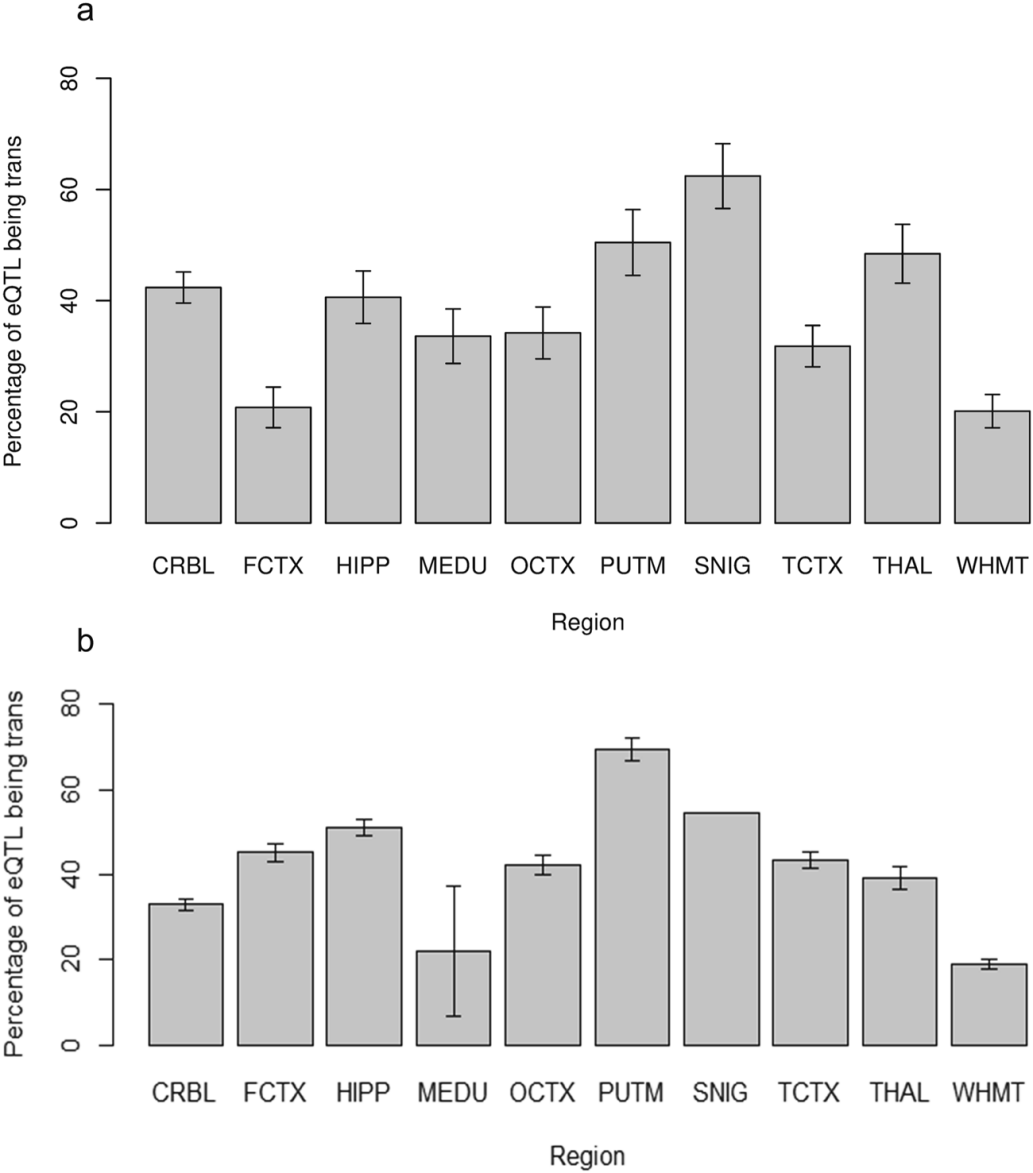
Percentage of eQTLs that are *trans*-acting at (**a**) transcript-level and (**b**) exon-level by brain regions. Note that these numbers are corresponding to eQTLs that were found at the transcript-level (i.e. includes transcript-only and a subset of “both”) and at the exon-level (i.e. includes exon-only and a subset of “both”). Error bars of both plots are model-based standard errors. At the transcript-level (**a**), the percentage varies across the brain regions with the frontal cortex (FCTX) and white matter (WHMT) showing a low percentage (~20%) of *trans*-acting eQTLs while the substantia nigra (SNIG) and putamen (PUTM) show a higher percentage (≥50%). At the exon-level (**b**), it is important to consider the significant interaction of region and chromosome. The error bar for SNIG is not included and the error bar for the medulla (MEDU) is relatively large because for these regions, there are chromosomes with no *trans*-acting eQTLs (e.g. there are no exon-level *trans*-acting eQTLs on transcript Chr 21 in SNIG and MEDU).

**Figure 11:**
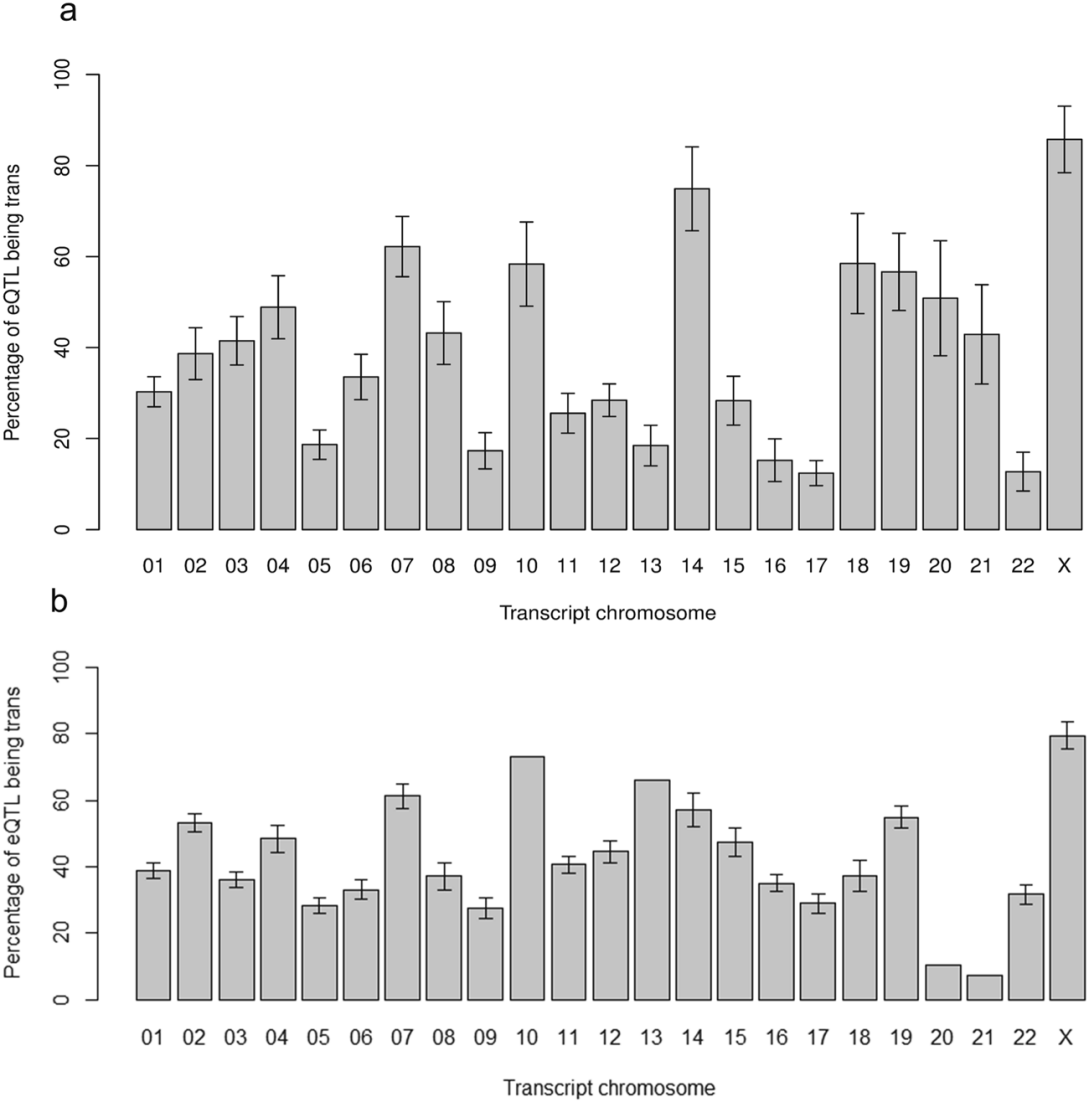
Percentage of eQTLs that are *trans*-acting at (**a**) transcript-level and (**b**) exon-level by chromosomes. Note that these numbers are corresponding to eQTLs that were found at the transcript-level (i.e. includes transcript-only and subset of “both”) and at the exon-level (i.e. includes exon-only and a subset of “both”). Error bars of both plots are model-based standard errors. At the transcript-level (**a**), the percentage of *trans*-acting eQTLs clearly varies between chromosomes with some chromosomes having less than 20% of eQTLs being *trans*-acting (i.e. Chr 5, 9, 13, 16, 17 and 22) while other chromosomes having more than 50% of eQTLs being *trans*- (i.e. Chr 7, 10, 14, 18, 19 and X). At exon-level (**b**), the significant interaction of region and chromosome needs to be considered. The error bars are missing for chromosomes 10, 13, 20 and 21 because one or more regions may not have any *trans*-acting eQTLs (e.g. there are no exon-level *trans*-acting eQTLs on transcript Chr 20 in the substantia nigra (SNIG).

Firstly, we looked at the numbers of *cis*-acting versus *trans*-acting eQTL and how they differed across regions and chromosomes using a logistic regression analysis. Results showed that regions had a significant effect (*P* = 1.5 × 10^−10^) on the percentage of *trans*-acting eQTLs at the transcript-level: WHMT has the lowest percentage of *trans*-acting eQTLs (20.1% ± 3.0%) and SNIG the highest (62.4% ± 5.8%) (see Fig. 10a). This variation between regions was also found at the exon-level where WHMT has the lowest percentage of *trans*-eQTLs at 18.9% ± 1.3% while PUTM has the highest at 69.4% ± 2.7% (see Fig. 10b). Interestingly, the percentage of *cis*-acting versus *trans*-acting differed significantly between chromosomes at the transcript-level (*P* = 3.68 × 10^−21^), from Chr 17: 12.4% ± 2.8% *trans*-acting, up to Chr X: 85.7% ± 7.3% *trans*-acting (see Fig. 11a). Similarly, at the exon-level, there was a variation between chromosomes: Chr 21 had the lowest percentage of *trans*-eQTLs at 7.1% while Chr X had the highest at 79.6% ± 4.1% (see Fig. 11b).

**Figure 12:**
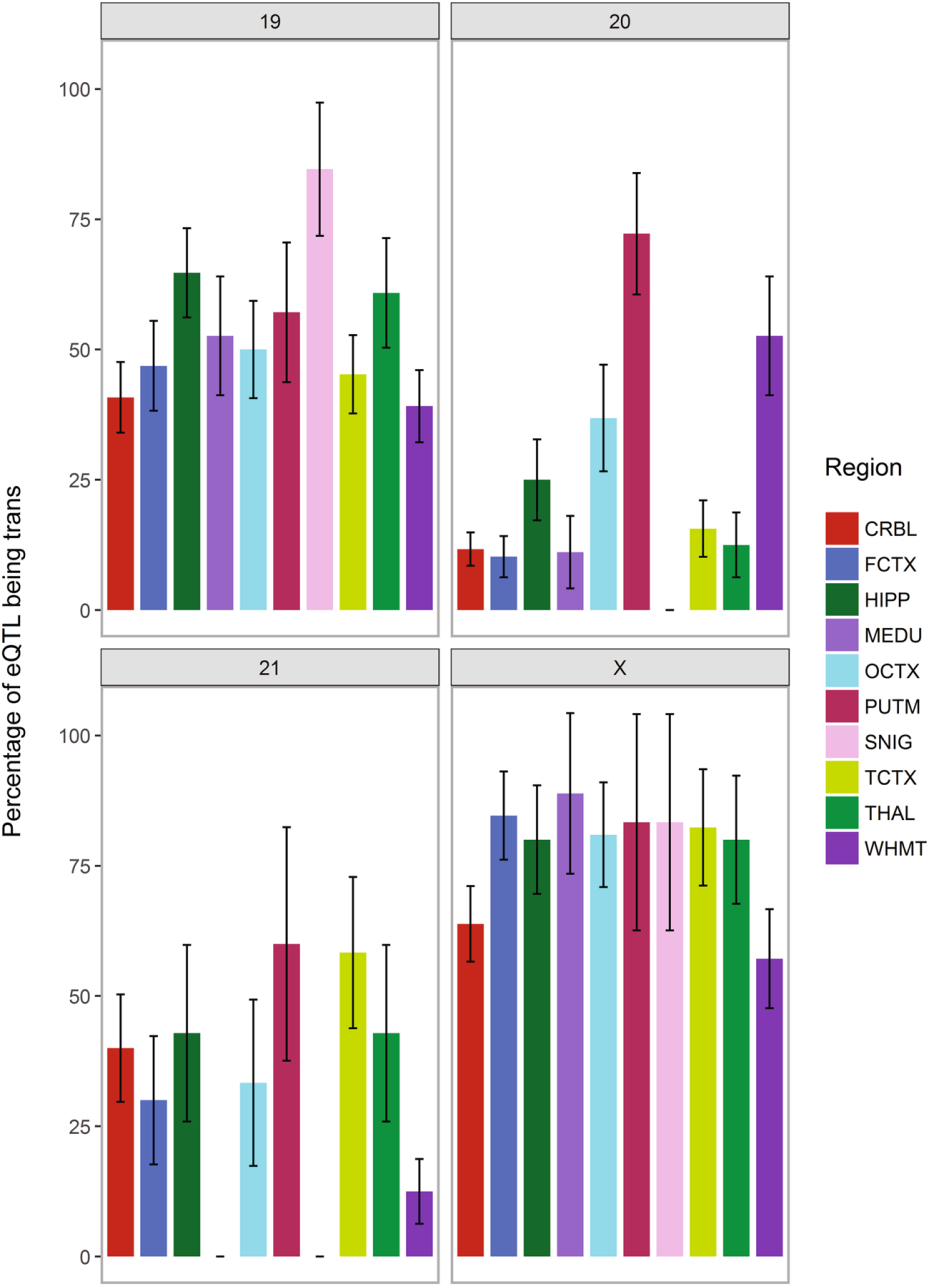
Percentage of exon-level eQTLs that are *trans*-acting for all regions on selected chromosomes. Transcript Chr 19, 20, 21 and X were chosen to demonstrate the significant interaction between region and chromosome on the percentage of exon-level *trans*-acting eQTLs. For example, across the four chromosomes, the putamen (PUTM) shows a constantly high percentage of *trans*-acting eQTLs (also seen in (**b**) while the substantia nigra (SNIG) has more variability, high on transcript Chr 19 and X but none on transcript Chr 20 and 21. Transcript Chr X shows a high percentage of *trans*-acting eQTLs for all regions, highlighting what was shown in (**a**).

Intriguingly, there was a highly significant region × chromosome interaction (*P* = 2.35 × 10^−49^) effect at the exon-level but not at the transcript-level (*P* = 1.00). Figure 12 illustrates this significant exon-level interaction for Chr 19, 21, 22 and X across the ten regions (see Supplementary Fig. S19 for the interactions between all 23 chromosomes and 10 regions). SNIG had high percentages of *trans*-acting eQTLs in Chr 19 and Chr X but none in Chr 20 and 21. MEDU showed a similar pattern with high percentages of *trans*-acting eQTLs in Chr 19 and Chr X but none Chr 21. On the other hand, WHMT showed a contrary pattern where it had higher percentages of *trans*-acting in Chr 20 and 21 but lower percentages in Chr 19 and X (when compared to SNIG and MEDU). These observations suggest that at the exon-level, the percentage of *trans*-acting eQTLs across the 10 regions are variable between different chromosomes; while at the transcript-level, the effect of the interaction of region and chromosome is uniform (e.g. if a region has a high percentage of *trans*-acting eQTL, it is held true for all chromosomes). This suggests that different combinations of chromosomes carrying specific set of genes may affect targeted biological processes affecting the underlying mechanism of certain diseases. Further functional biological studies using these observations are required to validate the concept.

#### Effect sizes in *cis*- versus *trans*-eQTLs

**Figure 13:**
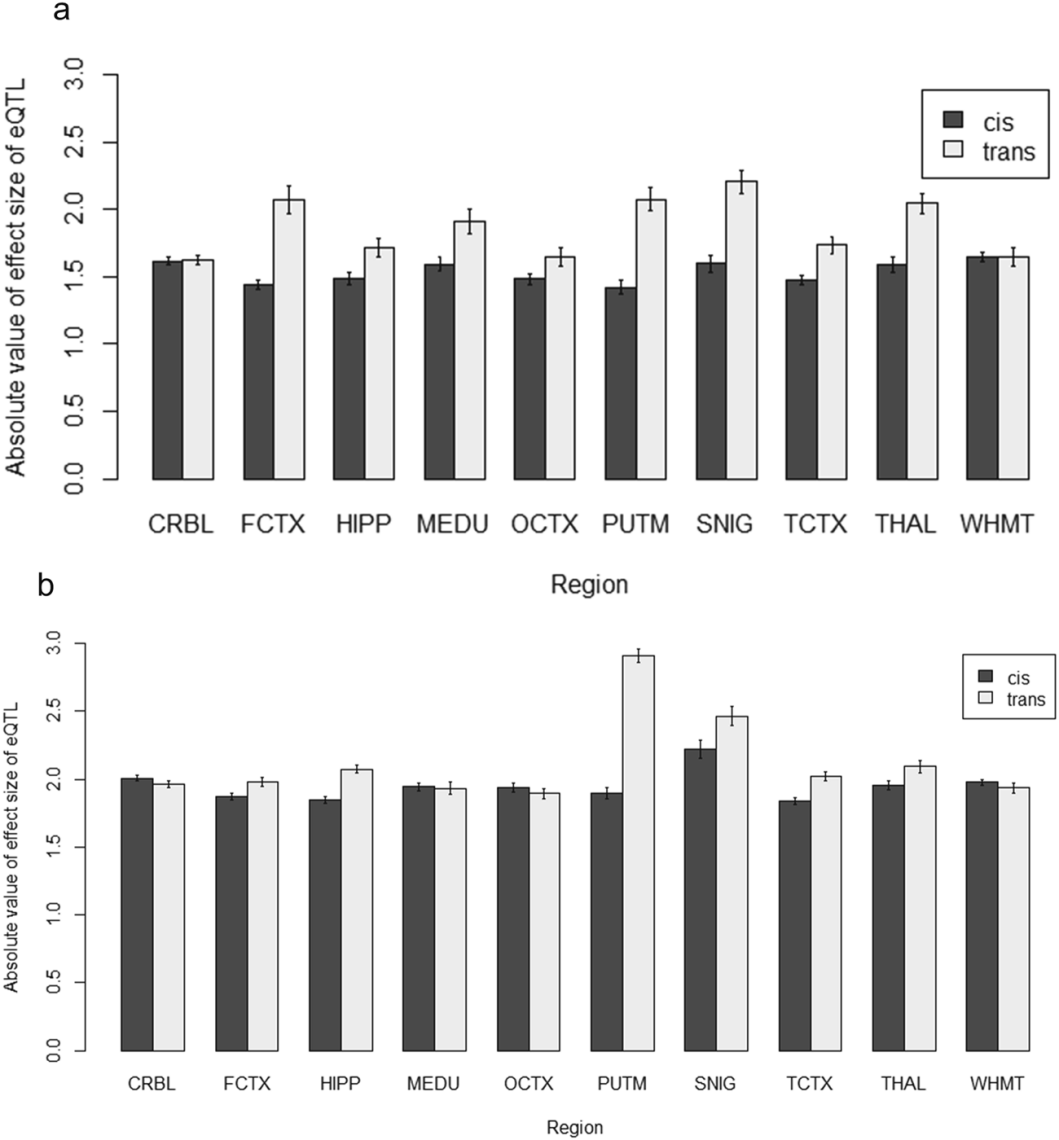
eQTL effect sizes for *cis*- versus *trans*-acting by brain regions at (**a**) transcript-level and (**b**) exon-level. These are the absolute values of effect sizes for *cis*- and *trans*-acting eQTLs by ten brain regions. On average, the absolute values of *trans*-acting eQTL effect sizes are larger than that of *cis*-acting eQTLs. This can be seen more clearly at the transcript-level (**a**) than at the exon-level (**b**). At the transcript-level, the frontal cortex (FCTX), putamen (PUTM) and substantia nigra (SNIG) show the largest difference in effect sizes between *cis*- and *trans*-acting eQTLs. At the exon-level, the cerebellum (CRBL), medulla (MEDU), occipital cortex (OCTX) and white matter (WHMT) show slightly larger *cis*-acting eQTL effect sizes than *trans*-acting.

**Figure 14:**
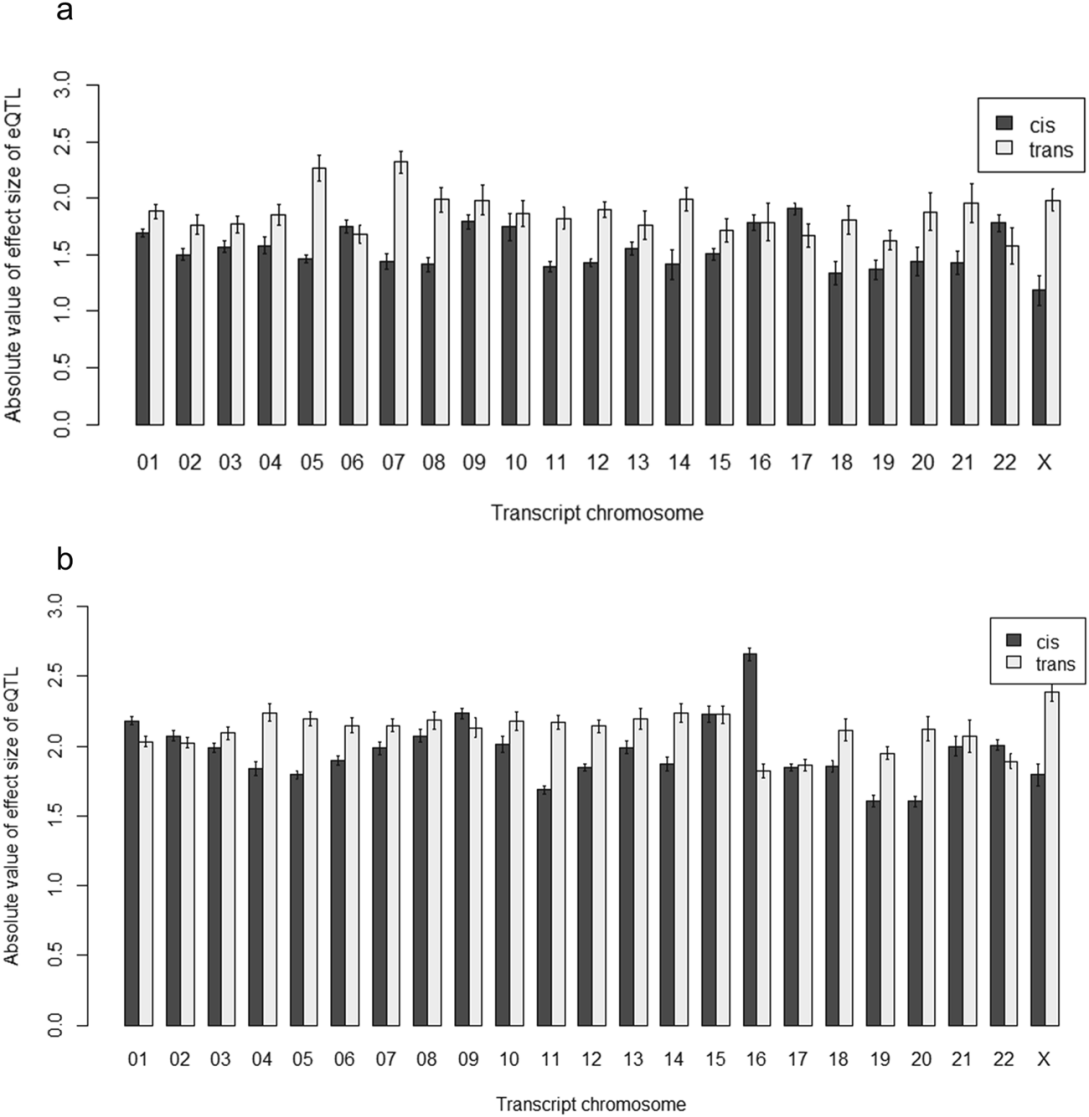
eQTL effect sizes for *cis*- versus *trans*-acting by transcript chromosome at (**a**) transcript-level and (**b**) exon-level. These are the absolute values of effect sizes for *cis*- and *trans*-acting eQTLs by chromosomes. On average, the absolute values of *trans*-acting eQTL effect sizes are larger than that of *cis*-acting eQTLs. This can be seen more clearly at the transcript-level (**a**) than at the exon-level (**b**). Note that the eQTLs used for these plots are all the eQTLs found at the transcript-level and exon-level respectively. There are some chromosomes where the absolute effect sizes of *cis*-acting eQTLs are larger than that of the *trans*-acting: transcript Chr 6, 17 and 22 at the transcript-level and transcript Chr 1, 2, 9, 16 and 22 at the exon-level. Transcript Chr 16 showed a large increase in *cis*-acting effect sizes going from the transcript-level to the exon-level.

Given that most MR-eQTLs were *cis*-acting with similar effect sizes across regions, we used a linear model to see if this pattern applied to eQTLs in general, in particular to see how eQTL effect size varied between brain regions, between chromosomes and between *cis*- versus *trans*-eQTLs. Overall, we found that the (absolute values of) effect sizes (|β|) of *trans*-eQTLs were on average larger than those of *cis*-eQTLs (effect sizes are taken as the allele substitution effect). Specifically, there was a significant *cis*/*trans* × region interaction for eQTL effect size at both the transcript-level (*P* = 1.07 × 10^−14^) and exon-level (*P* = 7.35 × 10^−48^). From Fig. 13a, the greatest difference at the transcript-level was observed for PUTM: *trans* = 2.08 ± 0.09, *cis* = 1.42 ± 0.05, while the smallest difference was observed for CRBL: *trans* = 1.63 ± 0.03, *cis* = 1.61 ± 0.03. Noticeably, there was no observed difference for WHMT. In addition, at the exon-level PUTM showed the greatest difference: *trans* = 2.91 ± 0.05, *cis* = 1.90 ± 0.04, whereas the smallest difference at the exon-level was observed for MEDU: *trans* = 1.93 ± 0.05, *cis* = 1.94 ± 0.03 (see Fig. 13b). It is also worth noting, at the exon-level, there were four regions (CRBL, MEDU, OCTX and WHMT) where the average (absolute) *cis*-eQTL effect sizes were greater than *trans*-eQTL effect sizes. The largest in that direction was found to be CRBL: *trans* = 1.96 ± 0.03, *cis* = 2.01 ± 0.02. Similarly, there was a significant *cis*/*trans* × chromosome interaction at the transcript-level (*P* = 8.36 × 10^−16^) and exon-level (*P* = 3.60 × 10^−81^). From Fig. 14a, Chr 7 showed the greatest difference between effect sizes with *trans*: 2.32 ± 0.10 and *cis*: 1.44 ± 0.07. However, on three chromosomes (Chr 6, 17 and 22), the average (absolute) effect sizes of *cis*-eQTLs were greater than that of *trans*-eQTLs. The largest difference in that direction was found in Chr 17 where *trans* = 1.67 ± 0.10 against *cis* = 1.91 ± 0.05. At the exon-level, Chr X showed the greatest difference where the absolute value of *trans*-eQTL effect sizes were greater than that of *cis*-eQTLs: *trans* = 2.386 ± 0.061, *cis* = 1.796 ± 0.079 (see Fig. 14b). On the other hand, there were seven chromosomes (Chr 1, 2, 9, 15, 16 and 22) where the average (absolute) effect sizes were greater in *cis*-eQTLs than *trans*-eQTLs. The chromosome with the greatest difference in this direction was Chr 16: *trans* = 1.824 ± 0.046, *cis* = 2.658 ± 0.043. This was also the greatest difference in any direction. Surprisingly, at the transcript-level, Chr 16 showed the smallest difference: *trans* = 1.788 ± 0.07, *cis* = 1.787 ± 0.168.

## Discussion

In this study, in-depth analyses were performed to obtain more insights from the public UKBEC dataset. This commenced with evaluating the UKBEC sample size through subsampling and simulation studies to address the sensitivity and specificity to detect eQTLs. This was followed by the genome-wide mapping of eQTLs in ten brain regions which were then used to study their patterns, particularly in terms of multi/single-region eQTLs, exon-level eQTLs and *trans*-acting eQTLs. In this study, a decision was made to only use genotyped SNPs. While a common practice is to use imputation for missing SNP genotypes, it was considered that including additional SNPs was not essential, given the number of SNPs that are in tight linkage disequilibrium. Indeed, after the initial eQTL mapping, there were redundant SNPs in LD associated with eQTLs that needed to be removed. A web-based visualisation interactive tool (Shiny app) was also developed to visualise and interrogate different patterns of eQTLs at multiple levels, making it a valuable tool in this area.

An important finding from the simulation part of this study was that only a small fraction of eQTLs were detected, given that sensitivity was generally under 5%. This suggests that only the ‘tip of the iceberg’ is being discovered. This finding was repeated across a range of model assumptions as in practice, we do not know the particular conditions in which the eQTL mapping data were generated. It is of course possible to discover more eQTL, but this would be at the expense of decreased specificity resulting in an excess of “false positives”. This underpins the need to maintain a stringent FDR threshold (≤0.01 as used in this study). Considering that only a partial set of genome-wide eQTLs is being detected, an important concern is how robust and reliable subsequent downstream analyses might be. For example, functional genomic network analysis is one of the main analyses used following this type of study and results should be looked at carefully. Nonetheless, these simulations did support that the number of samples used in this study which ranged from *n*_SNIG_ = 101 to *n*_WHMT_ = 131, is sufficient and as a result we recommend a bare minimum of 100 samples for eQTL mapping.

A key area investigated in this paper is the existence of MR-eQTLs, where one eQTL was mapped to more than one region, with extreme examples being mapped to all 10 regions. It was found that these MR-eQTLs have similar effect sizes within each region that they were acting in. Also, most of these MR-eQTL were *cis*-acting in contrast to many *trans*-acting eQTLs which tend to be unique to specific regions. Of particular note, there are four *cis*-acting MR-eQTLs that were present in all ten regions at the transcript-level with comparable effect sizes, indicating that these MR-eQTLs are more likely to have an impact on gene functions which are important for the brain as a whole. One example of this is SNP *rs5760176* associated with the gene *GSTT1* (glutathione S-transferase theta 1). Interestingly, SNP *rs5760176* (which is located within the deleted fragment of *GSTT1*) has been related to the null genotype (a homozygous deletion of part of *GSTT1*)^20^. There have been multiple studies of the *GSTT1* null genotype including an increased risk of brain tumours in UK European individuals^21^. In this study, the minor allele (*A*) of this SNP is associated with an increase of the transcript expression level (Supplementary Fig. S20). The encoded protein is part of the theta class of the GST superfamily that has been shown to play a critical role in the protection against oxidative stress and toxic chemicals within the cell^22^. This suggests that in the normal human brains, the increased expression levels of *GSTT1* may play a protective role in the oxidative stress mechanism.

Another MR-eQTL (SNP *rs1133328*, minor allele *G*) is associated with a decreased in the expression of protein coding gene *EFHB* (EF-hand domain family member B) across all ten regions (Supplementary Fig. S21). *EFHB* is still understudied but a recent study has shown that it may play a role in cellular Ca^2+^ mechanisms^23^. Furthermore, unlike the previous MR-eQTL example, the effect size of this MR-eQTL in CRBL was smaller compared to the other regions, highlighting the distinctive pattern of eQTLs in CRBL.

This leads us to highlight an interesting point: the uniqueness of CRBL compared to the other brain regions. Remarkably, CRBL has six times more SR-eQTLs compared with other regions and the lowest number of shared MR-eQTLs. The most significant *cis*-acting SR-eQTL (*rs10886711*) in the CRBL affects the expression of the *PLPP4* (phosphatidic acid phosphatase type 2 domain containing 1 A) transcript where the ‘*G*’ allele is associated with a decrease in transcript expression level (Supplementary Fig. S22). This association is confirmed by another eQTL brain study^9^. In a previous GWAS, the *PPLP4* gene is one of the top genes associated with cognitive decline in Alzheimer’s disease^24^. However, the nominated SNP in the GWAS study is not in the same LD block as our significant SNP. Therefore, *PLPP4* needs to be studied further to understand its relation to some brain mechanisms. Another SR-eQTL (*rs4688690*) associated with the *ZCCHC13* (Zinc Finger CCHC-Type containing 13) gene in the CRBL is *trans*-acting. The ‘*A*’ allele is associated with a decrease in *ZZCHC13* transcript expression (Supplementary Fig. S23). Further investigation into the gene needs to be done as there is a limitation of information about how this gene may link to brain diseases.

Major findings were obtained in this study are related to *trans*-eQTL in comparison to *cis*-eQTL: no other studies have made a systematic comparison of the number of *cis*- versus *trans*-eQTL, nor *cis* versus *trans* effect sizes using human brain, that we are aware of. There were differences in the ratio of *cis*-eQTL versus *trans*-eQTL between regions of the brain, and chromosomes. Another important finding was larger effect sizes of *trans*-acting eQTLs compared to *cis*-acting eQTLs in some chromosomes and brain regions. This was the case for both transcript-level and exon-level eQTLs. This contrasts with previous eQTL studies^9,14^ which suggested that the effect sizes of *cis*-acting eQTL are larger than *trans*-acting eQTL. A possible explanation for the differences in results from Gibbs, *et al*.^9^ were that they were investigating all four brain regions as a whole rather than at a regional level and without formal statistical testing as we have done. Furthermore, Grundberg, *et al*.^14^ was looking at different tissue types (adipose and LCLs) which may show different eQTL patterns to brain tissue. Interestingly, *trans*-acting eQTLs were more likely to be a SR-eQTL which suggests that there is a complex and unique system of interaction between genes that regulate activity within a particular brain region. In addition, there were some haplotypes that were singularly associated with many expression traits in *trans*. This reinforces the idea that these *trans*-acting eQTLs have a complex pattern in multiple brain regions. However, these findings in relation to *trans*-eQTL need to be confirmed using a larger cohort size in various tissues and cell types in addition to functional biological studies.

Through the in-depth analyses undertaken in this study, more insights into the patterns of genome-wide eQTLs in the human brain were gained, especially in terms of *trans*-eQTLs and multi-region patterns. Future investigations using advanced platforms and tools, for example long RNA-sequencing analysis, are required to study the contrast of eQTLs across different brain regions and different human tissues/cell types in more depth.

## Methods

### Collection of biological data

The brain tissue samples, DNA extraction and genotyping, together with the generation of the gene expression array data, are as described by Trabzuni, *et al*.^18^ and Ramasamy, *et al*.^11^. However, a brief summary of the collection procedure is provided here. In total, 134 human brain samples of European descent were obtained; all were classified as neurologically normal, and ages at death ranged from 16 to 102 years old (median 59 years old). From each brain, tissue was extracted from ten regions of the brain, namely cerebellum (CRBL, from *n* = 130 brains), frontal cortex (FCTX, *n* = 127), hippocampus (HIPP, *n* = 122), medulla (specifically inferior olivary nucleus, MEDU, *n* = 119), occipital cortex (specifically primary visual cortex, OCTX, *n* = 129), putamen (PUTM, *n* = 129), substania nigra (SNIG, *n* = 101), temporal cortex (TCTX, *n* = 119), thalamus (THAL, *n* = 124), and intralobular white matter (WHMT, *n* = 131). Variation in the number of regions sampled per brain was due to the practicality of extracting sufficient tissue form each region.

RNA was extracted from each region of each sample and processed using Affymetrix Human Exon 1.0 ST arrays. Only probe sets with at least three uniquely hybridising probes that were free of the polymorphism-in-probe problem were used. Expression levels were extracted from the remaining 291,705 exon-level probe sets, and transcript-level expression was calculated for 26,493 transcripts by calculating the Winsorised mean expression of all probe sets corresponding to each gene, as identified by using Netaffx annotation file Release 31 (HuEx-1_0-st-v2 Probeset Annotations). Finally, the expression data were residual-adjusted for brain bank, gender and batch effects.

Samples were genotyped on the Illumina Infinium Omni1-Quad BeadChip array. Overall, 1 million SNPs were genotyped, but only 788,474 of these SNPs were used in the analysis. A filter of the major allele frequency (MAF) > 5% was then applied, reducing the SNP set to 787,220 (i.e. 99.8% of SNPs had MAF > 5%). Next, any SNP that was missing in any of the 134 samples was omitted, reducing the number of SNPs available to 720,851, i.e. 91.6% of these SNPs had a complete set of genotypes. The advantages of this filter is that each SNP has equal power for detection of eQTLs *a priori*, i.e. no bias is introduced by some SNPs having fewer replicates, with consequent loss of power. Further details about SNP selection are shown in Supplementary Materials (S1).

### Expression QTL mapping

Due to the computational burden of assessing a large number of potential expression-SNP associations, a simple linear regression approach was used to map eQTLs. The R package MatrixEQTL^17^ was used as a computationally-efficient method of eQTL detection, with the eQTL effect size being the estimated regression coefficient (i.e. allele substitution effect for the minor allele on the expression phenotype). The Benjamini-Hochberg procedure was used for false discovery rate (FDR) control^25^, as part of MatrixEQTL, and a threshold of FDR < 0.01 was used to identify significant eQTLs. Note that the same procedure was used for the analysis of real and simulated data (see below). For the real data, a separate eQTL analysis was performed for each of the 10 brain regions, and separately for transcript-level and exon-level (region-by-region eQTL section).

For the real data, eQTLs with the same transcript (or the same exon, for exon-level eQTLs) with adjacent SNPs having an *R*^2^ over 0.5 were identified: these SNPs were considered as being in sufficient linkage disequilibrium, to represent a single block of SNPs, and hence a single eQTL. The eQTL with the highest significance in the block was identified, and the other redundant eQTLs in that block were discarded. This procedure was repeated separately for each brain region. Following this, a list of eQTLs across all 10 regions was complied.

### Sample size evaluation: analysis of transcript-level expression from cerebellum (CRBL) (real data)

As a first step towards evaluating the adequacy of the number of brain samples available for this study, eQTLs from the cerebellum were used as a model for other regions (before redundant SNPs were removed). Four sample sizes were evaluated (*n* = 100, 50, 25, and 13) against the original *n* = 130 brains. Each sample size was replicated ten times (randomly selected without replacements). MatrixEQTL was used to identify the number of eQTLs for each generated data set, and those in common with the original full analysis (*n* = 130 brains) was determined.

### Sample size evaluation: Simulations data and different Scenarios

Following the random selection of CRBL real data, R v 3.4.2^26^ was used to simulate data. For simplicity, the number of genetic markers (*n*_SNP_) and gene transcripts (*n*_trs_) were both set at 20,000 for all simulations and scenarios. Based off the sample sizes available from the UKBEC, six sample sizes were chosen (*n*_sample_ = 50, 100, 150, 200, 250, 300) and for every sample size, 100 simulations were run. These simulation parameters were kept constant for all five scenarios tested: 1: SNP genotypes in linkage equilibrium (LE); 2: SNP genotypes in linkage disequilibrium (LD); 3: SNP genotypes with genotyping error (GE); 4: lower expression level variance compared with residual variance (LV) and 5: dominance effect (Dom); (i.e. for each scenario, there were 100 simulations for the six sample sizes. So, a total of 600 simulations were run for each scenario, resulting in a grand total of 3,000 simulations for the study.

The method of each scenario is based on the simplest scenario, LE and parameters are kept constant unless otherwise described.

#### Scenario 1: SNP genotypes in linkage equilibrium (LE)

Allele frequencies of SNPs were randomly generated from a beta distribution with shape parameters *a* = *b* = 0.7, producing a set of SNPs which tend to have either high or low allele frequencies as observed in human SNP data. Only simulated SNPs with allele frequencies greater than 0.05 and less than 0.95 were kept and was used as the genotype probability to generate a matrix of genotype data using a binomial sampling distribution (0, 1 or 2 copies of the allele, at each SNP with genotype frequencies (1 − *p*_*i*_) ^2^, 2(1 − *p*_*i*_)*p*_*i*_, *p*_*i*_^2^)), assuming Hardy-Weinberg equilibrium.

Expression QTL effects, as allele substitution (additive) effects, were simulated in two steps, first simulating the occurrence of *trans*- and *cis*-eQTLs, followed by simulating the effect sizes, i.e. β values. The probability of a SNP having *trans*-acting eQTLs was simulated from a beta distribution with shape parameters *a* = 0.0004 and *b* = 10. The resulting probabilities were used as the probability of success for a binomial distribution to simulate *trans*-eQTLs. *Cis*-eQTLs were generated based on a binomial distribution with a 0.05 probability. For both *cis*- and *trans*-eQTLs, 1 corresponds with a simulated eQTL and 0 to the absence of a simulated eQTL. This matrix of 0 s and 1 s was multiplied by a matrix of eQTL effects generated from a normal distribution, *N*(0, σ^2^_β_) where σ_β_ = 1.15, resulting in a matrix of eQTL effects. Note that no minimum effect size was imposed on these eQTL effect sizes.

An additional matrix of random errors was simulated from a normal distribution, *N*(0, σ^2^_ε_) where σ_ε_ = 1. Transcript expression values were simulated using the sum of these two matrices (genotype data × eQTL effects, random errors).

#### Scenario 2: SNP genotypes in linkage disequilibrium (LD)

In the LD scenario, correlated SNPs were simulated at the genotype data simulation step instead of independent SNPs. For more details, see Supplementary Materials (S2).

#### Scenario 3: SNP genotypes with genotyping error (GE)

In this scenario, another SNP genotype matrix with genotyping errors was generated in the genotype simulation step. Genotyping error parameters were calculated from the cross-classification of SNPs from microarrays and sequencing reported by Rogers, *et al*.^27^ where the sequencing data were assumed to be more accurate and thus considered the “true genotype”^27^ (Supplementary Materials (S3)). This table (Supplementary Table S1) was used to create an “error” SNP genotype matrix that was used as the genotyping data for the R package MatrixEQTL^17^ as opposed to the “true” SNP genotype (as in the LE scenario).

#### Scenario 4: Lower expression level variance (LV)

In this scenario, eQTL effects were generated from a normal distribution, *N*(0, σ^2^_β_) where σ_β_ = 0.85, as opposed to σ_β_ = 1.15 (as in the LE scenario).

#### Scenario 5: Dominance Effect (Dom)

In addition to the additive effects simulated (as in LE scenario), dominance effects were simulated randomly from a normal distribution, *N*(0, σ^2^_D_) where σ_D_ = 0.25. From a matrix of SNP genotyped (as in LE scenario), another matrix where the homozygous genotypes were recoded as 0 and the heterozygous genotypes recoded as 1 was produced. Similar to the LE scenario, transcript expression values were calculated based on a linear model but with the dominance effects added.

With all scenarios, the R package MatrixEQTL^17^ was used to identify eQTLs followed by a filtering step where the false discovery rate (FDR) threshold (0.01) was set. These “detected eQTLs” and simulated “true” eQTLs were used to calculate the false positive (FP), false negative (FN), true positive (TP) and true negative (TN) eQTLs for a range of eQTL effect size thresholds, *k* (0 ≤ *k* ≤ 3). eQTLs were included in the calculations if the absolute values of their effect sizes (|β|) were equal to or above the threshold (i.e. |β| ≥ *k*). Following this, the sensitivity (Se) and specificity (Sp) were calculated where the sensitivity is the proportion of simulated eQTLs of a certain effect size or greater being correctly identified as eQTLs and the specificity is the proportion of non-existent eQTLs, i.e. background noise, being identified as such, i.e. Se = TP/(TP + FN), and Sp = TN/(TN + FP). The average sensitivity and specificity for the 100 simulations of each sample size for each scenario were then calculated.

In addition, three FDR thresholds (0.10, 0.05 and 0.01) were used to filter “detected eQTLs” for the LE scenario when *n* = 150 and *k* = 2.

### Combining lists of transcript-level and exon-level eQTLs

To combine the lists of eQTLs detected at transcript-level and exon-level, exon-level eQTLs corresponding to a transcript were identified. Where a transcript-level eQTL was identified, and none of its corresponding exons were eQTLs, it was considered “transcript-only”. Similarly, if the exon-level eQTL identified within a transcript cluster had no transcript-level eQTL identified, it was considered “exon-only”. For eQTLs with some exon-level eQTLs within a transcript which also had a transcript-level eQTL, these were considered “both”. Please note that the both eQTLs category depend on the length (number of exons) of the corresponding transcript.

### *Cis*- and *trans*-acting eQTLs

Based on exploratory plots of distances between the transcript (or exon) and the SNP on the same chromosome, undertaken on a log scale, a cut-off distance of 10^6.5^ bp = 3.16 Mb was used, i.e. if the distance was under 3.16 Mb it was classified *cis*-acting, otherwise is was classified *trans*-acting (Supplementary Materials (S4)). To assess patterns of *cis*- versus *trans*-acting eQTLs, logistic regression was used (with *trans* coded as ‘1’, *cis* as ‘0’), with explanatory variables of brain region, chromosome, as well as their interaction. In addition, effect sizes of eQTLs (absolute value of regression coefficient from MatrixEQTL) were analysed using a linear model, with explanatory variables of *cis* versus *trans*, region, and chromosome, as well as their interactions. Both these analyses were undertaken using ASReml-R^28^.

### Single-region (SR-eQTLs) and Multi-region (MR-eQTLs)

To investigate the existence of SR/MR-eQTLs, i.e. an eQTL expressed in one region only or more than one brain region respectively, eQTLs of the same transcript ID (or same probe-level ID) and with nearby SNP positions (*R*^2^ > 0.5) were identified, and considered the same eQTLs, operating in all these regions. The numbers of SR/MR-eQTLs are summarised in Tables 3 and 6 in detail for all categories (transcript/exon-only, both, *cis* and *trans* as outlined previously). The different patterns of multi-region eQTLs in terms of frequency distributions were explored. Effect sizes of eQTLs were also compared across regions for transcript-level and exon-level eQTLs.

### 3D visualisation web tool “Shiny app”

As a means of visualising MR-eQTLs and SR-eQTLs, an interactive 3D app was constructed, with the size of the node (sphere) representing the number of eQTLs detected in that region, and the width of the line connecting the nodes indicating the number of eQTLs in common between that pair of regions. This has been undertaken for all eQTLs detected for transcript or exon levels, as well as subsets of eQTLs (i.e. *cis*-acting and *trans*-acting). This visualisation was created using the rgl package^29^ and the networkd3 package in R^30^. To make it accessible outside of the R environment, these visualisations were published using shiny^31^.

## Supplementary information

Supplementary Material

## Data Availability

UKBEC dataset analysed in the current study is a public dataset and have been previously published (PMID: 24264146, PMID: 25174004, PMID: 24519379) and available on Gene Expression Omnibus (GEO) using the accession code GSE30483 (http://www.ncbi.nlm.nih.gov/geo/query/acc.cgi?acc=GSE30483) and accession code GSE46706 (http://www.ncbi.nlm.nih.gov/geo/query/acc.cgi?acc=GSE46706) as well as on the following websites: (https://omictools.com/braineac-tool) and (http://www.braineac.org/). In addition, R code used and described in the study is available on the University of Sydney’s GitHub Repository at (https://github.sydney.edu.au/lsng7727/Genome-wide-human-brain-eQTL).
