# Author Correction: Genome-wide human brain eQTLs: In-depth analysis and insights using the UKBEC dataset

**DOI:** 10.1038/s41598-020-73067-3

**Published:** 2020-10-01

**Authors:** Letitia M. F. Sng, Peter C. Thomson, Daniah Trabzuni

**Affiliations:** 1 The University of Sydney, School of Life and Environmental Sciences, New South Wales, 2006 Australia; 2 Department of Neurodegenerative Diseases, UCL Queen Square Institute of Neurology, Queen Square, London, WC1N 3BG UK; 3 Department of Genetics, King Faisal Specialist Hospital and Research Centre, Riyadh, 11211 Saudi Arabia

Correction to: *Scientific Reports* https://doi.org/10.1038/s41598-019-55590-0, published online 16 December 2019

This Article contains errors:

A list of *Trans*-eQTLs identified using microarray expression values was previously omitted in the Article. It is now included below in Table 1.

**Table 1:** *Trans*-eQTLs identified using microarray expression values.

rsid	snp_chromo	snp_pos	gene	trs_Chr	trs_start	trs_stop	region	GWAS_Cat_AD	pvalue_Nalls	GWAS_Cat_PD
rs6668752	1	85003211	ANGPTL3	1	63063178	63071969	CRBL	Not reported	9.07E−01	Not reported
rs10158928	1	35473498	IL12RB2	1	67743735	67879005	SNIG	Not reported	2.64E−01	Not reported
rs4301626	1	159785370	ABCA4	1	94458401	94609628	MEDU	Not reported	6.03E−01	Not reported
rs2228565	1	40775937	SPAG17	1	118496298	118727845	CRBL	Not reported	9.69E−01	Not reported
rs4313407	1	221968779	TNR	1	175289727	175712896	WHMT	Not reported	8.15E−01	Not reported
rs7533324	1	165387533	CCNYL1	2	208576254	208626563	SNIG	Not reported	7.81E−01	Not reported
rs1874791	1	67806432	GLB1L	2	220100184	220110185	WHMT	Not reported	5.96E−01	Not reported
rs1874791	1	67806432	MLF1	3	158284254	158344870	WHMT	Not reported	5.96E−01	Not reported
rs10749775	1	67822087	DNAH12	3	57327747	57386253	WHMT	Not reported	6.82E−01	Not reported
rs10158928	1	35473498	RXFP1	4	159236477	159588848	SNIG	Not reported	2.64E−01	Not reported
rs7354949	1	58194530	IGFBP7	4	57896593	57976823	SNIG	Not reported	8.77E−01	Not reported
rs6668752	1	85003211	CENPE	4	104026970	104119566	CRBL	Not reported	9.07E−01	Not reported
rs12063430	1	24604438	LOC100289077	5	55592544	55796396	FCTX	Not reported	7.22E−01	Not reported
rs3753242	1	2069681	GRIN1	9	140032842	140063207	OCTX	Not reported	3.38E−01	Not reported
rs11571552	1	212792191	SRGN	10	70847838	70864554	TCTX	Not reported	9.36E−01	Not reported
rs2228565	1	40775937	TTC18	10	75013547	75118768	CRBL	Not reported	9.69E−01	Not reported
rs2228565	1	40775937	C10orf79	10	105889646	106061767	CRBL	Not reported	9.69E−01	Not reported
rs10749775	1	67822087	AGBL2	11	47681143	47736910	WHMT	Not reported	6.82E−01	Not reported
rs2228565	1	40775937	C12orf55	12	96883283	97040906	CRBL	Not reported	9.69E−01	Not reported
rs10749775	1	67822087	DNAH10	12	124265627	124298804	WHMT	Not reported	6.82E−01	Not reported
rs12028800	1	195071346	RNF17	13	25378426	25378504	FCTX	Not reported	8.05E−01	Not reported
rs228692	1	7880712	LOC100287415	14	75745593	75748974	CRBL	Not reported	5.58E−01	Not reported
rs16824543	1	1871337	LOC390595	15	65394693	65394817	THAL	Not reported	9.16E−02	Not reported
rs6429443	1	244171992	FBXL16	16	742501	762015	CRBL	Not reported	6.57E−01	Not reported
rs10749775	1	67822087	WDR16	17	9479959	9546764	WHMT	Not reported	6.82E−01	Not reported
rs10749775	1	67822087	DNAH9	17	11501748	11873465	WHMT	Not reported	6.82E−01	Not reported
rs163402	1	194817966	ATP2A3	17	3827195	3867771	TCTX	Not reported	9.86E−01	Not reported
rs12040095	1	220393414	SOCS3	17	76343619	76362494	HIPP	Not reported	3.50E−01	Not reported
rs12035579	1	40586438	LOC100288797	20	2779562	2800920	WHMT	Not reported	4.83E−01	Not reported
rs11579420	1	230896238	L3MBTL2	22	41605136	41613611	SNIG	Not reported	2.41E−01	Not reported
rs10749775	1	67822087	CXorf22	X	35937871	36015542	WHMT	Not reported	6.82E−01	Not reported
rs3792095	2	234238949	PPFIA4	1	202995724	203047863	WHMT	Not reported	8.18E−01	Not reported
rs1876716	2	235252788	ABCA4	1	94458401	94609628	CRBL	Not reported	7.85E−02	Not reported
rs1876716	2	235252788	F5	1	169482580	169555999	CRBL	Not reported	7.85E−02	Not reported
rs6729076	2	168295284	TNR	1	175289727	175712896	THAL	Not reported	6.50E−01	Not reported
rs6754133	2	115536791	FLJ45340	1	243216891	243279123	SNIG	Not reported	3.08E−01	Not reported
rs17046413	2	55071861	MAP2	2	210288791	210598834	FCTX	Not reported	8.75E−01	Not reported
rs3792095	2	234238949	KCNH7	2	163200662	163695500	WHMT	Not reported	8.18E−01	Not reported
rs2304773	2	234235820	GRM7	3	6863238	7806217	WHMT	Not reported	7.62E−01	Not reported
rs10195337	2	223400520	GPR128	3	100328445	100419603	CRBL	Not reported	2.28E−01	Not reported
rs6531084	2	16559585	GPR128	3	100328445	100419603	FCTX	Not reported	9.04E−01	Not reported
rs10495790	2	33719473	PVRL3	3	110789318	111358749	CRBL	Not reported	5.81E−01	Not reported
rs3792095	2	234238949	KALRN	3	123752230	124238687	WHMT	Not reported	8.18E−01	Not reported
rs11677415	2	64471185	LIPH	3	185221674	185290286	MEDU	Not reported	7.26E−01	Not reported
rs1876716	2	235252788	ATP13A5	3	192992102	193110456	CRBL	Not reported	7.85E−02	Not reported
rs1990851	2	15256606	DRD5	4	9782216	9786571	PUTM	Not reported	3.21E−01	Not reported
rs3792095	2	234238949	LNX1	4	54321985	54658057	WHMT	Not reported	8.18E−01	Not reported
rs1876716	2	235252788	PRLR	5	35048865	35264354	CRBL	Not reported	7.85E−02	Not reported
rs17046413	2	55071861	CAP2	6	17393658	17558009	FCTX	Not reported	8.75E−01	Not reported
rs2540050	2	201575564	HIST1H2AC	6	26124373	26140084	PUTM	Not reported	7.44E−02	Not reported
rs6729076	2	168295284	HIST1H1E	6	26156559	26157343	THAL	Not reported	6.50E−01	Not reported
rs4387746	2	168330745	HIST1H1E	6	26156559	26157343	THAL	Not reported	8.16E−01	Not reported
rs3792095	2	234238949	GPLD1	6	24424793	24496179	WHMT	Not reported	8.18E−01	Not reported
rs111832461	2	186132403	CRISP1	6	49784415	49916170	THAL	Not reported	5.04E−02	Not reported
rs3792095	2	234238949	ICA1	7	8127802	8324787	WHMT	Not reported	8.18E−01	Not reported
rs17046413	2	55071861	PCLO	7	82585650	82586251	FCTX	Not reported	8.75E−01	Not reported
rs10495790	2	33719473	SEMA3D	7	84624528	84816513	CRBL	Not reported	5.81E−01	Not reported
rs17046413	2	55071861	PDP1	8	94929103	94969970	FCTX	Not reported	8.75E−01	Not reported
rs1996289	2	146108380	PKHD1L1	8	110374628	110543484	HIPP	Not reported	5.11E−01	Not reported
rs10189550	2	13517891	RNF122	8	33397634	33430151	SNIG	Not reported	1.64E−01	Not reported
rs3769464	2	201216280	DNAI1	9	34457432	34551542	WHMT	Not reported	5.64E−01	Not reported
rs6741535	2	142501896	FBP2	9	97321002	97356094	CRBL	Not reported	4.33E−01	Not reported
rs17046413	2	55071861	C9orf4	9	111897903	111929571	FCTX	Not reported	8.75E−01	Not reported
rs1876716	2	235252788	SLC39A12	10	18228207	18370880	CRBL	Not reported	7.85E−02	Not reported
rs17321444	2	23108116	ZNF518A	10	97857613	97923502	OCTX	Not reported	6.35E−01	Not reported
rs17321444	2	23108116	LCOR	10	98719621	98719863	OCTX	Not reported	6.35E−01	Not reported
rs17046413	2	55071861	KCNMA1	10	78635716	78635883	FCTX	Not reported	8.75E−01	Not reported
rs17321444	2	23108116	TIAL1	10	121301491	121356531	OCTX	Not reported	6.35E−01	Not reported
rs17046413	2	55071861	C11orf41	11	33693187	33707692	FCTX	Not reported	8.75E−01	Not reported
rs2216536	2	15265510	CBL	11	119076917	119178852	PUTM	Not reported	4.46E−01	Not reported
rs15628	2	37872871	OR8G5	11	124120423	124135763	CRBL	Not reported	4.56E−01	Not reported
rs17321444	2	23108116	HCFC2	12	104458339	104500295	OCTX	Not reported	6.35E−01	Not reported
rs6434893	2	197818806	PZP	12	9288945	9360966	CRBL	Not reported	2.32E−01	Not reported
rs1462060	2	6550266	PZP	12	9288945	9360966	PUTM	Not reported	1.19E−01	Not reported
rs17321444	2	23108116	WBP11	12	14938214	14956904	OCTX	Not reported	6.35E−01	Not reported
rs17046413	2	55071861	SLC2A13	12	40150765	40152741	FCTX	Not reported	8.75E−01	Not reported
rs3792095	2	234238949	GLS2	12	56864742	56882181	WHMT	Not reported	8.18E−01	Not reported
rs16855610	2	197504764	RASSF9	12	86197986	86230297	CRBL	Not reported	2.50E−01	Not reported
rs10490431	2	17108634	HPD	12	122277457	122326507	MEDU	Not reported	3.34E−01	Not reported
rs198688	2	158270960	PCDH8	13	53323563	53430763	FCTX	Not reported	8.43E−01	Not reported
rs3792095	2	234238949	SYT16	14	62278712	62568427	WHMT	Not reported	8.18E−01	Not reported
rs17321444	2	23108116	CNIH	14	54831664	54908284	OCTX	Not reported	6.35E−01	Not reported
rs17046413	2	55071861	MYO5A	15	52603264	52603398	FCTX	Not reported	8.75E−01	Not reported
rs75758327	2	168115797	CHSY1	15	101649177	101792119	THAL	Not reported	7.00E−01	Not reported
rs17321444	2	23108116	RBL2	16	53468372	53527880	OCTX	Not reported	6.35E−01	Not reported
rs17321444	2	23108116	MBP	18	74724754	74894075	OCTX	Not reported	6.35E−01	Not reported
rs2602204	2	159127641	CLEC4G	19	7793850	7796988	CRBL	Not reported	9.37E−02	Not reported
rs17046413	2	55071861	UNC13A	19	17712155	17714454	FCTX	Not reported	8.75E−01	Not reported
rs13389571	2	217874931	OTOR	20	16729003	16749094	CRBL	Not reported	6.41E−02	Not reported
rs1876716	2	235252788	CLIC6	21	36003007	36090519	CRBL	Not reported	7.85E−02	Not reported
rs17046413	2	55071861	PDK3	X	24560663	24561501	FCTX	Not reported	8.75E−01	Not reported
rs3792095	2	234238949	DRP2	X	100474275	100517065	WHMT	Not reported	8.18E−01	Not reported
rs1876716	2	235252788	HTR2C	X	113818551	114144623	CRBL	Not reported	7.85E−02	Not reported
rs3772295	3	1374539	C1orf51	1	150254963	150259499	MEDU	Not reported	6.56E−01	Not reported
rs9850692	3	599626	ABCA4	1	94458401	94609628	CRBL	Not reported	2.03E−01	Not reported
rs6763067	3	54036605	ADAMTS4	1	161154110	161169647	OCTX	Not reported	9.02E−01	Not reported
rs9850692	3	599626	F5	1	169482580	169555999	CRBL	Not reported	2.03E−01	Not reported
rs13064993	3	149403563	SELE	1	169691810	169703200	WHMT	Not reported	4.73E−01	Not reported
rs16858887	3	146496302	TBR1	2	162271078	162282381	CRBL	Not reported	6.49E−01	Not reported
rs7620792	3	132694401	LOC440925	2	171568969	171571067	THAL	Not reported	3.09E−01	Not reported
rs1403725	3	142444133	ZNF518B	4	10446096	10446650	TCTX	Not reported	7.46E−01	Not reported
rs1403725	3	142444133	SLC6A3	5	1345874	1445550	TCTX	Not reported	7.46E−01	Not reported
rs16858887	3	146496302	MOXD1	6	132568464	132722672	CRBL	Not reported	6.49E−01	Not reported
rs308739	3	4376030	SOD2	6	160100096	160183551	HIPP	Not reported	9.16E−01	Not reported
rs1513375	3	131480369	LOC285902	7	64330560	64350223	TCTX	Not reported	6.76E−01	Not reported
rs6765484	3	50041313	C7orf42	7	66395349	66396191	CRBL	Not reported	8.48E−02	Not reported
rs2681780	3	49897830	C7orf42	7	66395349	66396191	THAL	Not reported	3.41E−01	Not reported
rs6763067	3	54036605	FLNC	7	128470470	128499317	OCTX	Not reported	9.02E−01	Not reported
rs9290042	3	159140615	LOC100294067	7	4175724	4175942	CRBL	Not reported	3.90E−01	Not reported
rs13089169	3	56608033	ARHGEF3	7	147539172	147539604	CRBL	Not reported	1.58E−01	Not reported
rs3821414	3	56762245	ARHGEF3	7	147539172	147539604	CRBL	Not reported	5.69E−01	Not reported
rs13089169	3	56608033	ARHGEF3	7	147539172	147539604	FCTX	Not reported	1.58E−01	Not reported
rs1489948	3	56757717	ARHGEF3	7	147539172	147539604	FCTX	Not reported	6.06E−01	Not reported
rs13089169	3	56608033	ARHGEF3	7	147539172	147539604	HIPP	Not reported	1.58E−01	Not reported
rs3821414	3	56762245	ARHGEF3	7	147539172	147539604	HIPP	Not reported	5.69E−01	Not reported
rs13089169	3	56608033	ARHGEF3	7	147539172	147539604	MEDU	Not reported	1.58E−01	Not reported
rs3821414	3	56762245	ARHGEF3	7	147539172	147539604	MEDU	Not reported	5.69E−01	Not reported
rs3821414	3	56762245	ARHGEF3	7	147539172	147539604	OCTX	Not reported	5.69E−01	Not reported
rs3821414	3	56762245	ARHGEF3	7	147539172	147539604	TCTX	Not reported	5.69E−01	Not reported
rs1489948	3	56757717	ARHGEF3	7	147539172	147539604	THAL	Not reported	6.06E−01	Not reported
rs9871961	3	175529750	PI15	8	75736779	75762317	MEDU	Not reported	4.51E−01	Not reported
rs17395659	3	100014606	CRH	8	67088612	67090842	PUTM	Not reported	5.18E−01	Not reported
rs1403725	3	142444133	SLC18A2	10	118991784	119039223	TCTX	Not reported	7.46E−01	Not reported
rs6798316	3	36012538	IAPP	12	21507914	21532912	OCTX	Not reported	3.42E−01	Not reported
rs6763067	3	54036605	GPD1	12	50497799	50505087	OCTX	Not reported	9.02E−01	Not reported
rs9866764	3	30404343	WIBG	12	56322021	56322118	TCTX	Not reported	6.69E−01	Not reported
rs9850692	3	599626	SCNN1A	12	6456015	6486653	CRBL	Not reported	2.03E−01	Not reported
rs16858887	3	146496302	KIAA0748	12	55313725	55378510	CRBL	Not reported	6.49E−01	Not reported
rs10933844	3	106081419	DLK1	14	101193263	101201525	THAL	Not reported	7.14E−01	Not reported
rs7618466	3	6154807	KCNH5	14	63089146	63568752	PUTM	Not reported	1.84E−01	Not reported
rs6806885	3	115340985	CYP19A1	15	51471990	51630787	WHMT	Not reported	4.52E−01	Not reported
rs12152251	3	190915008	DENND4A	15	65950404	66084622	CRBL	Not reported	6.20E−01	Not reported
rs9850692	3	599626	ST6GALNAC2	17	74559749	74589466	CRBL	Not reported	2.03E−01	Not reported
rs2735247	3	125477762	MBP	18	74724754	74894075	MEDU	Not reported	9.40E−01	Not reported
rs9850692	3	599626	NWD1	19	16830797	16928764	CRBL	Not reported	2.03E−01	Not reported
rs9850692	3	599626	SLC5A5	19	17982764	18005983	CRBL	Not reported	2.03E−01	Not reported
rs7626401	3	190583974	LOC728533	19	38283661	38283884	FCTX	Not reported	7.81E−01	Not reported
rs308739	3	4376030	FPR1	19	52248435	52255150	THAL	Not reported	9.16E−01	Not reported
rs6763067	3	54036605	C21orf37	21	18811228	18821483	OCTX	Not reported	9.02E−01	Not reported
rs10933844	3	106081419	CHODL	21	19273590	19639682	THAL	Not reported	7.14E−01	Not reported
rs4688690	3	50022292	ZCCHC13	X	73524025	73540464	CRBL	Not reported	1.49E−01	Not reported
rs3774745	3	50204745	ZCCHC13	X	73524025	73540464	CRBL	Not reported	1.88E−01	Not reported
rs2526743	3	50115245	ZCCHC13	X	73524025	73540464	OCTX	Not reported	2.25E−01	Not reported
rs3774749	3	50207227	ZCCHC13	X	73524025	73540464	OCTX	Not reported	2.19E−01	Not reported
rs6763067	3	54036605	MBNL3	X	131444464	131624111	OCTX	Not reported	9.02E−01	Not reported
rs11937784	4	164340073	DNAJC8	1	28527088	28527811	CRBL	Not reported	1.47E−01	Not reported
rs3774909	4	23828708	NGEF	2	233743418	233877960	SNIG	Not reported	9.23E−01	Not reported
rs6828005	4	7774352	LPP	3	188599225	188600149	THAL	Not reported	2.81E−01	Not reported
rs11945108	4	41283279	CCDC39	3	180320666	180588773	HIPP	Not reported	1.15E−01	Not reported
rs12650613	4	182459990	PRLR	5	35048865	35264354	CRBL	Not reported	4.89E−01	Not reported
rs62323939	4	123124149	PRLR	5	35048865	35264354	MEDU	Not reported	7.18E−01	Not reported
rs62324204	4	123547596	PRLR	5	35048865	35264354	MEDU	Not reported	7.83E−01	Not reported
rs1469240	4	65529405	ANKRD31	5	74441812	74532703	THAL	Not reported	1.86E−01	Not reported
rs7665116	4	23853011	PHACTR1	6	12695088	13302923	SNIG	Not reported	4.52E−01	Not reported
rs11945108	4	41283279	LOC100287718	6	46701273	46727223	HIPP	Not reported	1.15E−01	Not reported
rs11945108	4	41283279	MAK	6	10762891	10838782	HIPP	Not reported	1.15E−01	Not reported
rs3774909	4	23828708	EPHB6	7	142540198	142568840	SNIG	Not reported	9.23E−01	Not reported
rs61747381	4	113351822	HOXA4	7	27165830	27175659	TCTX	Not reported	2.59E−02	Not reported
rs894996	4	104418307	LOC286002	7	107297023	107302191	SNIG	Not reported	5.40E−01	Not reported
rs17676292	4	165161269	FABP4	8	82389906	82395478	HIPP	Not reported	9.23E−01	Not reported
rs62321947	4	123011263	SLC16A12	10	91190061	91316378	MEDU	Not reported	2.60E−01	Not reported
rs17314336	4	13181596	CPXM2	10	125465736	125656071	TCTX	Not reported	2.24E−01	Not reported
rs10516614	4	122973693	IGF2	11	2150360	2151150	TCTX	Not reported	6.96E−01	Not reported
rs3774909	4	23828708	PTPN5	11	18749479	18814553	SNIG	Not reported	9.23E−01	Not reported
rs3774909	4	23828708	CHRM1	11	62674920	62689332	SNIG	Not reported	9.23E−01	Not reported
rs13110084	4	24150414	RAB6A	11	73386575	73472201	HIPP	Not reported	9.43E−01	Not reported
rs61747381	4	113351822	KIF5A	12	57978624	57979296	TCTX	Not reported	2.59E−02	Not reported
rs10489129	4	106956108	SCNN1A	12	6456015	6486653	CRBL	Not reported	6.18E−01	Not reported
rs3774909	4	23828708	DDN	12	49378978	49393173	SNIG	Not reported	9.23E−01	Not reported
rs7665116	4	23853011	WIF1	12	65339931	65515301	SNIG	Not reported	4.52E−01	Not reported
rs7665116	4	23853011	AKAP5	14	64933825	64936425	SNIG	Not reported	4.52E−01	Not reported
rs7665116	4	23853011	AKAP5	14	64939005	64940143	SNIG	Not reported	4.52E−01	Not reported
rs10016298	4	157068585	TNFAIP2	14	103589522	103603776	CRBL	Not reported	3.04E−01	Not reported
rs13147094	4	39310882	MYO5A	15	52603264	52603398	SNIG	Not reported	8.86E−01	Not reported
rs3774909	4	23828708	KCNH4	17	40308913	40333296	SNIG	Not reported	9.23E−01	Not reported
rs13147094	4	39310882	ZNF134	19	58125625	58134895	SNIG	Not reported	8.86E−01	Not reported
rs3774909	4	23828708	RASD2	22	35936642	35961734	SNIG	Not reported	9.23E−01	Not reported
rs13147094	4	39310882	HDAC8	X	71549367	71792888	SNIG	Not reported	8.86E−01	Not reported
rs2229882	5	56168712	GRHL3	1	24562799	24690962	SNIG	Not reported	6.32E−01	Not reported
rs152528	5	142017860	LOC100132111	1	151814373	151822840	WHMT	Not reported	9.81E−02	Not reported
rs4921390	5	160802427	HSPA6	1	161494449	161496700	WHMT	Not reported	3.34E−01	Not reported
rs6883539	5	110779829	NUP210L	1	153965172	154127582	SNIG	Not reported	2.76E−02	Not reported
rs16897939	5	67888580	SLC5A7	2	108602636	108630668	THAL	Not reported	4.38E−01	Not reported
rs386073	5	9393029	HLA-DQA2	6	32714666	32715221	OCTX	Not reported	6.63E−02	Not reported
rs4257741	5	4481091	CHRNB3	8	42552562	42592530	TCTX	Not reported	2.89E−02	Not reported
rs4921390	5	160802427	SERPINH1	11	75260734	75283816	WHMT	Not reported	3.34E−01	Not reported
rs17630822	5	179943955	TSPAN9	12	3393551	3393682	THAL	Not reported	6.26E−01	Not reported
rs2195394	5	54247654	KIAA0748	12	55313725	55378510	CRBL	Not reported	2.43E−01	Not reported
rs2229882	5	56168712	C13orf33	13	31404792	31593362	SNIG	Not reported	6.32E−01	Not reported
rs1579319	5	54258180	REPS2	X	17167690	17171393	TCTX	Not reported	9.26E−01	Not reported
rs2195394	5	54247654	ZIC3	X	136568196	136659839	CRBL	Not reported	2.43E−01	Not reported
rs7771542	6	105660039	LOC284475	1	117041573	117041672	CRBL	Not reported	2.15E−01	Not reported
rs6456102	6	167107444	C2CD4D	1	151812679	151812827	TCTX	Not reported	8.23E−01	Not reported
rs16884828	6	75101820	MPZ	1	161274528	161279725	SNIG	Not reported	2.01E−02	Not reported
rs4896938	6	147994707	DNAH6	2	84931191	85046704	CRBL	Not reported	7.77E−01	Not reported
rs4896938	6	147994707	WDR69	2	228735665	228835163	CRBL	Not reported	7.77E−01	Not reported
rs7761230	6	112126303	IL1B	2	113587337	113602248	THAL	Not reported	1.62E−02	Not reported
rs16874275	6	13648864	UPK1B	3	118892372	118930566	FCTX	Not reported	9.79E−01	Not reported
rs16874275	6	13648864	UPK1B	3	118892372	118930566	OCTX	Not reported	9.79E−01	Not reported
rs2010219	6	14103048	ADAMTS9	3	64501336	64730291	THAL	Not reported	8.39E−01	Not reported
rs4896938	6	147994707	ZBBX	3	166912780	167098193	CRBL	Not reported	7.77E−01	Not reported
rs1981093	6	134581016	TNFAIP3	6	138184421	138304298	THAL	Not reported	4.83E−01	Not reported
rs2486420	6	149329220	CYP39A1	6	46464172	46620523	CRBL	Not reported	6.33E−01	Not reported
rs4896938	6	147994707	EFCAB1	8	49603701	49667383	CRBL	Not reported	7.77E−01	Not reported
rs114327274	6	32289594	C9orf107	9	105281928	105419791	CRBL	Not reported	9.13E−01	Not reported
rs3793137	6	39872504	PIK3C2A	11	17108123	17229523	HIPP	Not reported	6.64E−01	Not reported
rs7760902	6	128167328	TCN1	11	59620290	59649542	CRBL	Not reported	8.32E−01	Not reported
rs3793137	6	39872504	CCDC91	12	28286202	28733186	HIPP	Not reported	6.64E−01	Not reported
rs9689119	6	137873084	TMBIM6	12	50110497	50171829	OCTX	Not reported	6.19E−01	Not reported
rs4896938	6	147994707	LRRIQ1	12	85380615	85667645	CRBL	Not reported	7.77E−01	Not reported
rs3798936	6	161646714	COCH	14	31343767	31364773	HIPP	Not reported	1.69E−01	Not reported
rs4896938	6	147994707	LRRC9	14	60366373	60530277	CRBL	Not reported	7.77E−01	Not reported
rs7761230	6	112126303	ANGPTL4	19	8411399	8439246	SNIG	Not reported	1.62E−02	Not reported
rs7768422	6	110796405	ICAM1	19	10381765	10397289	THAL	Not reported	2.82E−01	Not reported
rs2182644	6	112138882	EMR2	19	14843205	14946068	THAL	Not reported	3.47E−02	Not reported
rs1334708	6	138481846	USHBP1	19	17359180	17375544	CRBL	Not reported	6.80E−01	Not reported
rs4896938	6	147994707	HTR2C	X	113818551	114144623	CRBL	Not reported	7.77E−01	Not reported
rs16869492	6	54082465	NDUFA1	X	119004438	119019714	TCTX	Not reported	7.03E−01	Not reported
rs2010219	6	14103048	CCL2	17	32489317	32584212	THAL	Not reported	8.39E−01	Not reported
rs10235765	7	36442113	CLCN6	1	11866135	11908118	HIPP	Not reported	2.30E−01	Not reported
rs10235765	7	36442113	HIAT1	1	100503662	100548916	HIPP	Not reported	2.30E−01	Not reported
rs66553243	7	28222812	KIAA1324	1	109656538	109749587	CRBL	Not reported	8.14E−01	Not reported
rs4563786	7	28248461	KIAA1324	1	109656538	109749587	CRBL	Not reported	6.64E−01	Not reported
rs7788171	7	76845398	SLC44A5	1	75667817	76076791	CRBL	Not reported	5.02E−01	Not reported
rs10235765	7	36442113	FAM69A	1	93298463	93427025	HIPP	Not reported	2.30E−01	Not reported
rs917098	7	44750079	CD48	1	160640369	160693839	MEDU	Not reported	2.87E−01	Not reported
rs10235765	7	36442113	CEP170	1	243287741	243418613	HIPP	Not reported	2.30E−01	Not reported
rs10235765	7	36442113	RAB10	2	26256835	26367556	HIPP	Not reported	2.30E−01	Not reported
rs10235765	7	36442113	RAB7A	3	128444979	128534045	HIPP	Not reported	2.30E−01	Not reported
rs10235765	7	36442113	HERC3	4	89501266	89630407	HIPP	Not reported	2.30E−01	Not reported
rs10235765	7	36442113	RAP1GDS1	4	99182567	99365012	HIPP	Not reported	2.30E−01	Not reported
rs10235765	7	36442113	KIAA1109	4	123167738	123283904	HIPP	Not reported	2.30E−01	Not reported
rs3735569	7	28215361	LNX1	4	54321985	54658057	CRBL	Not reported	8.74E−01	Not reported
rs4563786	7	28248461	LNX1	4	54321985	54658057	CRBL	Not reported	6.64E−01	Not reported
rs10235765	7	36442113	UBE2D3	4	103715543	103790032	HIPP	Not reported	2.30E−01	Not reported
rs10235765	7	36442113	TBCK	4	106927312	107240387	HIPP	Not reported	2.30E−01	Not reported
rs2704938	7	105989414	HPGD	4	175411165	175443955	WHMT	Not reported	1.54E−01	Not reported
rs7788171	7	76845398	GLRA3	4	175502020	175750465	CRBL	Not reported	5.02E−01	Not reported
rs3735569	7	28215361	KCTD16	5	143584850	143856934	CRBL	Not reported	8.74E−01	Not reported
rs4563786	7	28248461	KCTD16	5	143584850	143856934	CRBL	Not reported	6.64E−01	Not reported
rs10235765	7	36442113	MOCS2	5	52393916	52416327	HIPP	Not reported	2.30E−01	Not reported
rs2188403	7	92920114	PHACTR1	6	12695088	13302923	THAL	Not reported	1.51E−01	Not reported
rs4720305	7	4518110	NR2E1	6	108486754	108510009	MEDU	Not reported	8.92E−01	Not reported
rs10235765	7	36442113	MAP3K7	6	91223310	91320206	HIPP	Not reported	2.30E−01	Not reported
rs73072534	7	28144510	LOC286002	7	107297023	107302191	CRBL	Not reported	1.19E−01	Not reported
rs2709911	7	82937146	PI15	8	75736779	75762317	WHMT	Not reported	1.19E−01	Not reported
rs17144779	7	21647686	LOXL2	8	23154162	23282831	MEDU	Not reported	3.32E−01	Not reported
rs317722	7	29118935	C8orf84	8	73956434	74109897	TCTX	Not reported	8.25E−01	Not reported
rs3735569	7	28215361	C9orf91	9	117373640	117408840	CRBL	Not reported	8.74E−01	Not reported
rs10235765	7	36442113	BLOC1S1	12	56109777	56113484	HIPP	Not reported	2.30E−01	Not reported
rs317722	7	29118935	DAO	12	109268564	109294799	TCTX	Not reported	8.25E−01	Not reported
rs66553243	7	28222812	GRIN2B	12	13714144	14301613	CRBL	Not reported	8.14E−01	Not reported
rs17144779	7	21647686	SOCS3	17	76343619	76362494	SNIG	Not reported	3.32E−01	Not reported
rs7788171	7	76845398	DCC	18	49866843	51060014	CRBL	Not reported	5.02E−01	Not reported
rs1718618	7	68632251	ONECUT2	18	55152307	55153840	THAL	Not reported	8.69E−01	Not reported
rs317722	7	29118935	GYG2	X	2746859	2810005	TCTX	Not reported	8.25E−01	Not reported
rs2330339	7	53100319	VGLL1	X	135614311	135711808	CRBL	Not reported	6.26E−01	Not reported
rs3735569	7	28215361	DCX	X	110537018	110657756	CRBL	Not reported	8.74E−01	Not reported
rs1849490	7	28249048	DCX	X	110537018	110657756	CRBL	Not reported	9.47E−01	Not reported
rs1879678	8	27745205	KIF1B	1	10270548	10441651	HIPP	Not reported	6.31E−01	Not reported
rs2244368	8	11452638	ABCA4	1	94458401	94609628	CRBL	Not reported	6.36E−01	Not reported
rs4875408	8	4777221	PXDN	2	1635667	1748698	CRBL	Not reported	2.43E−01	Not reported
rs1879678	8	27745205	LRRFIP1	2	238536243	238674746	HIPP	Not reported	6.31E−01	Not reported
rs7820834	8	130250443	LOC100129449	2	153439499	153442408	TCTX	Not reported	7.68E−01	Not reported
rs4875129	8	4802339	LOC100287763	3	98241642	98244670	PUTM	Not reported	7.87E−01	Not reported
rs13274442	8	70883360	COL29A1	3	130064466	130064824	THAL	Not reported	3.23E−01	Not reported
rs1879678	8	27745205	YEATS2	3	183415606	183534905	HIPP	Not reported	6.31E−01	Not reported
rs7827166	8	15773120	LPP	3	187870811	188597210	TCTX	Not reported	6.86E−01	Not reported
rs1879678	8	27745205	HACL1	3	15580270	15643097	HIPP	Not reported	6.31E−01	Not reported
rs1879678	8	27745205	PDS5A	4	39811935	39979566	HIPP	Not reported	6.31E−01	Not reported
rs13439094	8	37125422	GABRA6	5	161111618	161129599	PUTM	Not reported	1.77E−01	Not reported
rs2244368	8	11452638	PRLR	5	35048865	35264354	CRBL	Not reported	6.36E−01	Not reported
rs1879678	8	27745205	DOPEY1	6	83777267	83881063	HIPP	Not reported	6.31E−01	Not reported
rs2155939	8	102847202	TXLNB	6	139480299	139613276	CRBL	Not reported	6.18E−01	Not reported
rs1879678	8	27745205	CALU	7	128379214	128413453	HIPP	Not reported	6.31E−01	Not reported
rs74583398	8	55311323	MGAM	7	141672558	141806541	PUTM	Not reported	1.67E−01	Not reported
rs1879678	8	27745205	COG5	7	106826219	107204696	HIPP	Not reported	6.31E−01	Not reported
rs6995553	8	129540236	DNA2	10	70173822	70239022	TCTX	Not reported	4.83E−01	Not reported
rs1980262	8	9906271	C1S	12	7096273	7096460	THAL	Not reported	4.10E−01	Not reported
rs13269996	8	40981047	PMCH	12	102584251	102625919	THAL	Not reported	1.16E−01	Not reported
rs2244368	8	11452638	KL	13	33552841	33644545	CRBL	Not reported	6.36E−01	Not reported
rs10956489	8	130727016	FLJ37307	13	52387483	52419499	SNIG	Not reported	4.80E−01	Not reported
rs17270119	8	35534138	ASXL3	18	31251711	31327690	CRBL	Not reported	9.86E−01	Not reported
rs7836513	8	69613880	SMAD7	18	46400890	46518500	TCTX	Not reported	3.49E−01	Not reported
rs13250313	8	18652812	OAZ1	19	2263854	2274028	CRBL	Not reported	9.76E−01	Not reported
rs2726559	8	59657936	ZNF738	19	21568643	21568982	CRBL	Not reported	5.92E−01	Not reported
rs2244368	8	11452638	CLIC6	21	36003007	36090519	CRBL	Not reported	6.36E−01	Not reported
rs17655533	8	14970795	GTSE1	22	46692757	46730193	WHMT	Not reported	3.13E−01	Not reported
rs16905072	8	135107424	XIST	X	73048903	73049066	THAL	Not reported	3.64E−01	Not reported
rs16931910	9	117618309	ATF3	1	212738696	212794097	CRBL	Not reported	7.37E−01	Not reported
rs7868935	9	90917641	C1orf115	1	220863187	220872483	CRBL	Not reported	6.28E−01	Not reported
rs34230770	9	140188045	BAI2	1	32192719	32229965	TCTX	Not reported	6.78E−01	Not reported
rs34230770	9	140188045	FBXO41	2	73481730	73517601	TCTX	Not reported	6.78E−01	Not reported
rs34230770	9	140188045	BSN	3	49591922	49708962	TCTX	Not reported	6.78E−01	Not reported
rs4526484	9	134910165	LOC100130701	3	120068377	120106964	OCTX	Not reported	7.14E−01	Not reported
rs16928181	9	128196221	RXFP1	4	159236477	159588848	CRBL	Not reported	7.68E−01	Not reported
rs2777877	9	72018961	CXCL10	4	76932369	76944669	THAL	Not reported	1.67E−01	Not reported
rs1535480	9	7925381	MCM3	6	52128780	52248684	TCTX	Not reported	8.48E−01	Not reported
rs1328293	9	16172580	EMID2	7	101006114	101248972	SNIG	Not reported	7.00E−01	Not reported
rs34230770	9	140188045	EPB49	8	21906521	21940030	TCTX	Not reported	6.78E−01	Not reported
rs34230770	9	140188045	RHOBTB2	8	22844311	22878201	TCTX	Not reported	6.78E−01	Not reported
rs16928181	9	128196221	GDA	9	74729573	74899450	CRBL	Not reported	7.68E−01	Not reported
rs10979157	9	110757887	ANO3	11	26154079	26684835	THAL	Not reported	3.58E−01	Not reported
rs10963871	9	18957669	MUC19	12	40920898	40920951	MEDU	Not reported	9.14E−02	Not reported
rs7868935	9	90917641	C12orf64	12	80645473	80672946	CRBL	Not reported	6.28E−01	Not reported
rs10125101	9	1184213	MFAP5	12	8789967	8853992	THAL	Not reported	8.17E−01	Not reported
rs716875	9	109520131	ABCD2	12	39945207	39945558	OCTX	Not reported	9.14E−01	Not reported
rs9696881	9	109274146	APEX1	14	20922688	20926246	TCTX	Not reported	8.48E−01	Not reported
rs10979157	9	110757887	FOXG1	14	29234449	29262375	THAL	Not reported	3.58E−01	Not reported
rs11243819	9	135406613	TMEM90A	14	74874238	74876504	MEDU	Not reported	4.05E−01	Not reported
rs10511650	9	18005255	CYP19A1	15	51471990	51630787	TCTX	Not reported	7.00E−02	Not reported
rs9696881	9	109274146	NTRK3	15	88406099	88406561	TCTX	Not reported	8.48E−01	Not reported
rs9696881	9	109274146	TMEM199	17	26684694	26690705	TCTX	Not reported	8.48E−01	Not reported
rs7870439	9	100951838	LOC728675	17	42722779	42722954	CRBL	Not reported	7.71E−01	Not reported
rs7868935	9	90917641	NETO1	18	70409551	70535468	CRBL	Not reported	6.28E−01	Not reported
rs321736	9	28753619	OR7D4	19	9315008	9345932	OCTX	Not reported	4.83E−02	Not reported
rs7043651	9	138114837	IL13RA2	X	114195370	114266684	WHMT	Not reported	4.16E−01	Not reported
rs16911135	10	59606236	FSIP2	2	186610156	186631276	CRBL	Not reported	5.57E−01	Not reported
rs9971243	10	68756845	GPR128	3	100328445	100419603	HIPP	Not reported	6.28E−01	Not reported
rs9971243	10	68756845	GPR128	3	100328445	100419603	PUTM	Not reported	6.28E−01	Not reported
rs9971243	10	68756845	GPR128	3	100328445	100419603	WHMT	Not reported	6.28E−01	Not reported
rs9416748	10	60590203	PLA1A	3	119316526	119348650	OCTX	Not reported	7.74E−01	Not reported
rs7894146	10	59371321	SI	3	164676877	164875690	CRBL	Not reported	2.39E−01	Not reported
rs877345	10	8053776	ENPEP	4	111397212	111531100	THAL	Not reported	1.29E−01	Not reported
rs587039	10	89807852	RXFP1	4	159236477	159588848	CRBL	Not reported	2.87E−02	Not reported
rs7894146	10	59371321	TECRL	4	65143420	65275174	CRBL	Not reported	2.39E−01	Not reported
rs587039	10	89807852	GABRA6	5	161111618	161129599	CRBL	Not reported	2.87E−02	Not reported
rs12218075	10	101900190	VNN2	6	133065010	133084598	SNIG	Not reported	4.20E−01	Not reported
rs7894146	10	59371321	ASZ1	7	117003276	117067555	CRBL	Not reported	2.39E−01	Not reported
rs7894146	10	59371321	LRRC19	9	26993144	27005695	CRBL	Not reported	2.39E−01	Not reported
rs2043090	10	77298609	KLHL1	13	70172464	70682591	TCTX	Not reported	7.60E−01	Not reported
rs7086215	10	121087695	PNPLA6	19	7599054	7626636	PUTM	Not reported	7.92E−01	Not reported
rs4750000	10	6165525	PTTG1IP	21	46246033	46294237	CRBL	Not reported	7.94E−01	Not reported
rs17298481	11	20675645	ABCA4	1	94458401	94609628	MEDU	Not reported	5.04E−01	Not reported
rs3862356	11	89150372	TTC21B	2	131489803	131490255	HIPP	Not reported	9.98E−01	Not reported
rs12419429	11	22572968	GALNT5	2	158114109	158175809	MEDU	Not reported	7.32E−01	Not reported
rs12295797	11	94304270	SERPINB1	6	2796160	2853674	TCTX	Not reported	4.29E−01	Not reported
rs2173000	11	133507330	DSERG1	9	3464712	3464938	PUTM	Not reported	6.80E−01	Not reported
rs7116819	11	13897617	MED15	22	20847344	20941902	HIPP	Not reported	2.95E−01	Not reported
rs10501219	11	40134083	DGKK	X	50117970	50158226	SNIG	Not reported	7.34E−01	Not reported
rs12822135	12	18558204	GRHL3	1	24562799	24690962	MEDU	Not reported	5.91E−01	Not reported
rs1800973	12	69744014	LOC100287934	1	224109930	224198578	CRBL	Not reported	7.90E−01	Not reported
rs11047073	12	23893317	MTHFD2	2	74425703	74444797	TCTX	Not reported	9.05E−01	Not reported
rs7136193	12	59316881	IL1B	2	113587337	113602248	THAL	Not reported	5.57E−01	Not reported
rs11169059	12	49924832	ARGFX	3	121289535	121289663	MEDU	Not reported	2.49E−03	Not reported
rs775451	12	70112707	ATP13A5	3	192992102	193110456	HIPP	Not reported	3.10E−01	Not reported
rs1800973	12	69744014	GABRA6	5	161111618	161129599	CRBL	Not reported	7.90E−01	Not reported
rs1800973	12	69744014	NA	6	80247215	80296555	CRBL	Not reported	7.90E−01	Not reported
rs7295770	12	116755819	HOXA4	7	27165830	27175659	WHMT	Not reported	9.94E−01	Not reported
rs638043	12	53044267	PROSC	8	37619675	37637427	OCTX	Not reported	7.45E−01	Not reported
rs17797090	12	104324266	SAMD12	8	119204488	119204782	TCTX	Not reported	7.10E−01	Not reported
rs1045603	12	101018161	DCAF12	9	34107143	34107643	CRBL	Not reported	2.91E−01	Not reported
rs1800973	12	69744014	ZNF503	10	77022456	77174998	CRBL	Not reported	7.90E−01	Not reported
rs11175392	12	64830924	ZNF408	11	46722368	46727461	WHMT	Not reported	2.03E−01	Not reported
rs623536	12	20450454	OR5T1	11	56043110	56044095	THAL	Not reported	5.88E−02	Not reported
rs1800973	12	69744014	CCND1	11	69452700	69469227	CRBL	Not reported	7.90E−01	Not reported
rs1800973	12	69744014	CHST1	11	45670431	45723808	CRBL	Not reported	7.90E−01	Not reported
rs17044922	12	78532738	ACACB	12	109554410	109706024	CRBL	Not reported	9.45E−01	Not reported
rs1163934	12	17601074	ADAMTS20	12	43699999	43965908	SNIG	Not reported	2.03E−01	Not reported
rs1800973	12	69744014	SOX1	13	112721105	112726020	CRBL	Not reported	7.90E−01	Not reported
rs1800973	12	69744014	ITPK1	14	93403259	93582697	CRBL	Not reported	7.90E−01	Not reported
rs2241220	12	109675029	C15orf54	15	39542895	39547038	TCTX	Not reported	7.74E−01	Not reported
rs1800973	12	69744014	THSD4	15	71413033	72075702	CRBL	Not reported	7.90E−01	Not reported
rs1800973	12	69744014	RGMA	15	93586646	93642479	CRBL	Not reported	7.90E−01	Not reported
rs11067218	12	115086078	SLC26A11	17	78194232	78227297	MEDU	Not reported	2.25E−01	Not reported
rs2936548	12	47867510	TBCD	17	80709925	80907425	CRBL	Not reported	3.85E−01	Not reported
rs1800973	12	69744014	EBF4	20	2672915	2758037	CRBL	Not reported	7.90E−01	Not reported
rs10506085	12	32630503	VSIG4	X	65241591	65294614	WHMT	Not reported	3.62E−02	Not reported
rs2230343	13	99948097	HSPA6	1	161494449	161496700	CRBL	Not reported	8.29E−01	Not reported
rs11616433	13	100162063	HSPA6	1	161494449	161496700	CRBL	Not reported	6.33E−01	Not reported
rs9568580	13	51938876	HFM1	1	91817106	91866666	MEDU	Not reported	7.43E−01	Not reported
rs17662661	13	59323804	PTX3	3	157122033	157161370	THAL	Not reported	2.92E−01	Not reported
rs7327537	13	88003946	DPP6	7	153445287	154685994	PUTM	Not reported	4.73E−01	Not reported
rs17591314	13	103222899	STC1	8	23663443	23717910	TCTX	Not reported	4.89E−01	Not reported
rs17252283	13	63594334	PTPN11	12	112856536	113056212	OCTX	Not reported	3.69E−01	Not reported
rs17252283	13	63594334	WASF3	13	27075110	27271698	OCTX	Not reported	3.69E−01	Not reported
rs2036130	13	82606614	ENOX1	13	43755076	44361106	CRBL	Not reported	3.45E−01	Not reported
rs17252283	13	63594334	SOS2	14	50583853	50584044	OCTX	Not reported	3.69E−01	Not reported
rs17252283	13	63594334	SPRED1	15	38544256	38681651	OCTX	Not reported	3.69E−01	Not reported
rs9576782	13	39964668	CCDC79	16	66788428	66835590	TCTX	Not reported	3.33E−02	Not reported
rs17252283	13	63594334	SMAD4	18	48554802	48607297	OCTX	Not reported	3.69E−01	Not reported
rs4597193	13	69343101	LOC100289661	19	40704282	40704591	FCTX	Not reported	3.76E−02	Not reported
rs7327537	13	88003946	NSFL1C	20	1422815	1448423	OCTX	Not reported	4.73E−01	Not reported
rs9516495	13	95581692	PRO0628	20	39665648	39666824	TCTX	Not reported	5.07E−02	Not reported
rs17089624	13	53358100	TSIX	X	73044489	73046527	TCTX	Not reported	9.21E−01	Not reported
rs1152509	14	56831820	HPCA	1	33351615	33364022	MEDU	Not reported	1.51E−01	Not reported
rs7154607	14	67866928	UGT2A1	4	70441062	70573679	CRBL	Not reported	8.32E−01	Not reported
rs1152509	14	56831820	SH3RF2	5	145316142	145461336	MEDU	Not reported	1.51E−01	Not reported
rs17107503	14	78871577	FBLL1	5	167955508	167957639	THAL	Not reported	8.40E−01	Not reported
rs17677652	14	83091423	C7orf68	7	128095718	128109225	FCTX	Not reported	5.66E−01	Not reported
rs1051340	14	92441066	LOC341378	12	106545715	106566215	THAL	Not reported	2.65E−01	Not reported
rs12879079	14	98530364	KRT5	12	52638832	52914477	CRBL	Not reported	5.91E−01	Not reported
rs1152509	14	56831820	FOXG1	14	29234449	29262375	MEDU	Not reported	1.51E−01	Not reported
rs1152561	14	56868616	TMEM90A	14	74872606	74874143	MEDU	Not reported	2.22E−01	Not reported
rs7154607	14	67866928	LOC284801	20	26167659	26190480	CRBL	Not reported	8.32E−01	Not reported
rs8034650	15	71798425	AK5	1	77747637	78025651	CRBL	Not reported	9.64E−01	Not reported
rs16976698	15	56330627	SIPA1L2	1	232508852	232697281	CRBL	Not reported	8.80E−01	Not reported
rs16976698	15	56330627	CAMK2A	5	149598962	149669467	CRBL	Not reported	8.80E−01	Not reported
rs7163671	15	61845418	CALB1	8	91068109	91188353	THAL	Not reported	5.90E−01	Not reported
rs16976698	15	56330627	C10orf58	10	82167601	82193852	CRBL	Not reported	8.80E−01	Not reported
rs11855944	15	78088127	C11orf73	11	86051634	86054777	HIPP	Not reported	9.30E−01	Not reported
rs8182039	15	53985450	BRF1	14	105675625	105781906	CRBL	Not reported	6.64E−01	Not reported
rs1224660	15	48000762	LOC645877	15	57025902	57138804	THAL	Not reported	4.33E−01	Not reported
rs9927776	16	7882110	WDR63	1	85464840	85599626	CRBL	Not reported	3.85E−01	Not reported
rs12596957	16	74349043	TBR1	2	162271078	162282381	CRBL	Not reported	8.25E−02	Not reported
rs9927776	16	7882110	WDR69	2	228735665	228835163	CRBL	Not reported	3.85E−01	Not reported
rs12934339	16	57610423	IBSP	4	88720702	88733952	THAL	Not reported	9.84E−01	Not reported
rs11864162	16	84046490	SH3RF2	5	145316142	145461336	MEDU	Not reported	5.20E−01	Not reported
rs36064924	16	23399312	FOLR1	11	71892921	71907337	MEDU	Not reported	8.78E−01	Not reported
rs1391229	16	86294764	LOC728147	12	2901259	2902150	CRBL	Not reported	3.51E−02	Not reported
rs39662	16	49489167	FLJ37307	13	52387483	52419499	MEDU	Not reported	6.09E−01	Not reported
rs10852312	16	27040683	STARD9	15	42979117	42979273	HIPP	Not reported	6.15E−01	Not reported
rs4888671	16	77783580	BCL6B	17	6926369	6933592	OCTX	Not reported	3.70E−01	Not reported
rs2304483	16	56917953	TIMM13	19	2425622	2428057	CRBL	Not reported	6.82E−01	Not reported
rs894621	17	55540976	PCLO	7	82585650	82586251	TCTX	Not reported	4.46E−01	Not reported
rs12603672	17	51115243	LOC100288975	10	91597930	91598276	WHMT	Not reported	7.86E−01	Not reported
rs1017346	17	8604154	ESAM	11	124623035	124633127	CRBL	Not reported	9.34E−01	Not reported
rs7216800	17	74885948	C12orf63	12	97041653	97159034	CRBL	Not reported	2.11E−03	Not reported
rs1017346	17	8604154	LIN7A	12	81191186	81331684	CRBL	Not reported	9.34E−01	Not reported
rs1017346	17	8604154	CA10	17	49692313	50258884	CRBL	Not reported	9.34E−01	Not reported
rs1017346	17	8604154	ERG	21	39722417	40033695	CRBL	Not reported	9.34E−01	Not reported
rs1017346	17	8604154	BRWD3	X	79927836	79928504	CRBL	Not reported	9.34E−01	Not reported
rs206614	18	10331765	CD200R1	3	112618234	112693956	FCTX	Not reported	7.03E−01	Not reported
rs16960057	18	33866095	CXCL10	4	76932369	76944669	OCTX	Not reported	4.12E−02	Not reported
rs16950303	18	46582359	MDGA1	6	37600293	37665766	OCTX	Not reported	5.71E−01	Not reported
rs7998	18	67671294	PSD	10	104162396	104179476	TCTX	Not reported	6.23E−02	Not reported
rs4987821	18	60813215	GLT8D2	12	104382499	104457951	THAL	Not reported	2.44E−01	Not reported
rs8089993	18	24854314	C22orf30	22	32111932	32112273	OCTX	Not reported	8.79E−01	Not reported
rs8103016	19	9250370	LRRIQ3	1	73942197	74552085	WHMT	Not reported	3.32E−01	Not reported
rs11673027	19	41098179	KRTCAP2	1	155141888	155151310	CRBL	Not reported	7.11E−02	Not reported
rs1820184	19	42245573	DOCK3	3	50927457	50927512	TCTX	Not reported	3.75E−02	Not reported
rs1820184	19	42245573	ABCC5	3	183637726	183735717	TCTX	Not reported	3.75E−02	Not reported
rs10406557	19	16250914	P4HA2	5	131563831	131564024	CRBL	Not reported	7.55E−01	Not reported
rs3826892	19	46388379	OAS1	12	113344602	113369975	CRBL	Not reported	7.55E−01	Not reported
rs1820184	19	42245573	TRMT5	14	61440320	61440759	TCTX	Not reported	3.75E−02	Not reported
rs4398207	19	610729	CYP19A1	15	51471990	51630787	TCTX	Not reported	2.46E−01	Not reported
rs10408632	19	7466835	DYX1C1	15	55709960	55800432	WHMT	Not reported	6.96E−01	Not reported
rs10421438	19	51994351	RUNDC3A	17	42385791	42396033	FCTX	Not reported	7.43E−01	Not reported
rs1003467	19	30991130	MYOM1	18	3044938	3247539	OCTX	Not reported	3.30E−01	Not reported
rs9305001	19	22783220	ACE2	X	15579162	15620409	SNIG	Not reported	1.88E−01	Not reported
rs13045538	20	33595525	CAMTA1	1	6845394	7829758	PUTM	Not reported	7.73E−02	Not reported
rs6101058	20	59336158	C1orf88	1	111867305	111900301	CRBL	Not reported	3.21E−01	Not reported
rs34075384	20	32963639	ATP1B1	1	169075580	169102322	PUTM	Not reported	2.50E−01	Not reported
rs1535044	20	44739156	EDN2	1	41884885	41950545	CRBL	Not reported	2.98E−01	Not reported
rs6101058	20	59336158	ABCA4	1	94458401	94609628	CRBL	Not reported	3.21E−01	Not reported
rs13045538	20	33595525	PCNXL2	1	233118775	233431486	PUTM	Not reported	7.73E−02	Not reported
rs13045538	20	33595525	TMEM178	2	39892658	39960570	PUTM	Not reported	7.73E−02	Not reported
rs13045538	20	33595525	FAM123C	2	131506377	131565684	PUTM	Not reported	7.73E−02	Not reported
rs13045538	20	33595525	AAK1	2	69685135	69930320	PUTM	Not reported	7.73E−02	Not reported
rs4405822	20	2050481	CCKAR	4	26473318	26558929	TCTX	Not reported	2.28E−01	Not reported
rs13045538	20	33595525	TBC1D9	4	141538721	141733197	PUTM	Not reported	7.73E−02	Not reported
rs34075384	20	32963639	RAB3C	5	57878029	58147791	PUTM	Not reported	2.50E−01	Not reported
rs4405822	20	2050481	SLC6A3	5	1345874	1445550	TCTX	Not reported	2.28E−01	Not reported
rs6101058	20	59336158	PRLR	5	35048865	35264354	CRBL	Not reported	3.21E−01	Not reported
rs13045538	20	33595525	TMEM14A	6	52535758	52586796	PUTM	Not reported	7.73E−02	Not reported
rs13045538	20	33595525	RIMS1	6	72596329	73177344	PUTM	Not reported	7.73E−02	Not reported
rs13045538	20	33595525	GRIK2	6	101841683	102690448	PUTM	Not reported	7.73E−02	Not reported
rs6101058	20	59336158	DNAH11	7	21582571	21951191	CRBL	Not reported	3.21E−01	Not reported
rs13045538	20	33595525	C8orf79	8	12803930	12865165	PUTM	Not reported	7.73E−02	Not reported
rs13045538	20	33595525	C8orf79	8	12883872	12884432	PUTM	Not reported	7.73E−02	Not reported
rs13045538	20	33595525	MIR124-1	8	9642110	9856682	PUTM	Not reported	7.73E−02	Not reported
rs13045538	20	33595525	DIRAS2	9	93372122	93405128	PUTM	Not reported	7.73E−02	Not reported
rs13045538	20	33595525	MADD	11	47290775	47351568	PUTM	Not reported	7.73E−02	Not reported
rs6101058	20	59336158	FOLR1	11	71892921	71907337	CRBL	Not reported	3.21E−01	Not reported
rs6042389	20	13834807	MMP8	11	102583185	102595661	CRBL	Not reported	9.72E−01	Not reported
rs13045538	20	33595525	OPCML	11	132278376	133402800	PUTM	Not reported	7.73E−02	Not reported
rs34075384	20	32963639	SLC2A13	12	40150765	40152741	PUTM	Not reported	2.50E−01	Not reported
rs13045538	20	33595525	HS6ST3	13	96742995	97520479	PUTM	Not reported	7.73E−02	Not reported
rs13045538	20	33595525	CALM1	14	90854917	90874600	PUTM	Not reported	7.73E−02	Not reported
rs13045538	20	33595525	MEG8	14	101359265	101402336	PUTM	Not reported	7.73E−02	Not reported
rs11700338	20	25595868	CLK3	15	74890853	74932052	CRBL	Not reported	2.03E−01	Not reported
rs11700338	20	25595868	IL16	15	81605605	81606398	CRBL	Not reported	2.03E−01	Not reported
rs13045538	20	33595525	NUP93	16	56742885	56881922	PUTM	Not reported	7.73E−02	Not reported
rs11700338	20	25595868	SMG1	16	70253494	70283941	CRBL	Not reported	2.03E−01	Not reported
rs11700338	20	25595868	PTBP1	19	796768	812505	CRBL	Not reported	2.03E−01	Not reported
rs6101058	20	59336158	SLC5A5	19	17982764	18005983	CRBL	Not reported	3.21E−01	Not reported
rs13045538	20	33595525	CHGB	20	5892117	5905983	PUTM	Not reported	7.73E−02	Not reported
rs11700338	20	25595868	TIAM1	21	32423201	32932280	CRBL	Not reported	2.03E−01	Not reported
rs11700338	20	25595868	HPS4	22	26839397	26879820	CRBL	Not reported	2.03E−01	Not reported
rs7277302	21	44183895	TBR1	2	162271078	162282381	CRBL	Not reported	6.93E−01	Not reported
rs7277302	21	44183895	KALRN	3	123752230	124238687	CRBL	Not reported	6.93E−01	Not reported
rs3850712	21	43093894	CXCL10	4	76932369	76944669	THAL	Not reported	8.96E−01	Not reported
rs7277302	21	44183895	PART1	5	59783781	59881123	CRBL	Not reported	6.93E−01	Not reported
rs2410215	21	41525807	LOC100132005	6	38147084	38149009	MEDU	Not reported	1.75E−01	Not reported
rs858027	21	39104180	FER1L6	8	124968386	125035836	SNIG	Not reported	2.89E−01	Not reported
rs7277302	21	44183895	GDA	9	74729573	74899450	CRBL	Not reported	6.93E−01	Not reported
rs7277302	21	44183895	KIAA0748	12	55313725	55378510	CRBL	Not reported	6.93E−01	Not reported
rs4920118	21	43319984	MTUS2	13	30078996	30085332	TCTX	Not reported	1.48E−01	Not reported
rs2834330	21	35366219	LIPG	18	47087079	47227325	TCTX	Not reported	3.08E−01	Not reported
rs16997746	21	32261641	ICAM1	19	10381765	10397289	FCTX	Not reported	2.06E−01	Not reported
rs1015020	21	24334028	FLJ16779	20	61886613	61887039	HIPP	Not reported	6.29E−01	Not reported
rs7277302	21	44183895	TIAM1	21	32423201	32932280	CRBL	Not reported	6.93E−01	Not reported
rs17181008	22	49809003	S100A9	1	153330330	153341061	SNIG	Not reported	7.01E−01	Not reported
rs5749232	22	31599664	PLXNA2	1	208197792	208200170	TCTX	Not reported	8.27E−01	Not reported
rs17181008	22	49809003	DSP	6	7541870	7587036	SNIG	Not reported	7.01E−01	Not reported
rs447017	22	20100158	GDA	9	74729573	74899450	SNIG	Not reported	6.33E−01	Not reported
rs447017	22	20100158	PDE2A	11	72287189	72385453	SNIG	Not reported	6.33E−01	Not reported
rs447017	22	20100158	CRYM	16	21255193	21314384	SNIG	Not reported	6.33E−01	Not reported
rs447017	22	20100158	PRKCG	19	54385461	54410899	SNIG	Not reported	6.33E−01	Not reported
rs6623842	X	86297381	WDR63	1	85464840	85599626	CRBL	Not reported	NA	Not reported
rs6623842	X	86297381	SPAG17	1	118496298	118727845	CRBL	Not reported	NA	Not reported
rs4503218	X	121930793	ASH1L	1	155305061	155532313	HIPP	Not reported	NA	Not reported
rs6640729	X	11346716	FLJ45340	1	243216891	243279123	SNIG	Not reported	NA	Not reported
rs17322868	X	41170168	SLC5A7	2	108602636	108630668	THAL	Not reported	NA	Not reported
rs17261124	X	118670532	GPR128	3	100328445	100419603	TCTX	Not reported	NA	Not reported
rs5934655	X	9557779	GPR98	5	89854393	90460232	THAL	Not reported	NA	Not reported
rs6623842	X	86297381	PRLR	5	35048865	35264354	CRBL	Not reported	NA	Not reported
rs12557891	X	141582899	C6orf103	6	146919922	147136595	CRBL	Not reported	NA	Not reported
rs6623842	X	86297381	DNAH11	7	21582571	21951191	CRBL	Not reported	NA	Not reported
rs12557891	X	141582899	CD36	7	79998901	80351915	CRBL	Not reported	NA	Not reported
rs7055508	X	95725865	SEMA3A	7	83421440	84121918	CRBL	Not reported	NA	Not reported
rs12559574	X	83309097	ASH2L	8	37963069	38008450	FCTX	Not reported	NA	Not reported
rs1442577	X	4148701	KIAA2026	9	6007195	6007787	SNIG	Not reported	NA	Not reported
rs6623842	X	86297381	ARMC3	10	23216891	23335197	CRBL	Not reported	NA	Not reported
rs6645723	X	116498965	KIAA0748	12	55313725	55378510	MEDU	Not reported	NA	Not reported
rs1198718	X	92472403	TMEM90A	14	74874238	74876504	HIPP	Not reported	NA	Not reported
rs11796333	X	141114829	UBE2MP1	16	34403742	34423018	THAL	Not reported	NA	Not reported
rs6523516	X	100851308	ONECUT2	18	55152307	55153840	SNIG	Not reported	NA	Not reported
rs6523516	X	100851308	ONECUT2	18	55155404	55175523	SNIG	Not reported	NA	Not reported
rs6523516	X	100851308	DGKK	X	50117970	50158226	SNIG	Not reported	NA	Not reported

The *trans*-eQTLs (520 variants) were merged through SNPs with the following datasets/databases:^1^ GWAS_Cat_AD: The GWAS Catalog with search parameters ‘Alzheimer’s Disease’;^2^ *p* value_Nalls: Parkinson’s Disease Meta-Analysis of GWAS by Nalls et al. (2019);^3^ GWAS_Cat_PD: The GWAS Catalog with search parameters ‘Parkinsons’s Disease’. SNPs/eQTLs combined with GWAS databases/datasets [i.e.^1,3^] are indicated either ‘Reported/Not reported’^2^. Nalls et al. *P-*values are available. Cells indicated by *NA* were not in the databases/datasets.

A list of *Trans*-eQTLS identified using RNA-Seq expression values was also omitted and is now included below as Table 2.

**Table 2:** *Trans*-eQTLs identified using RNA-Seq expression values.

rsid	snp_Chr	snp_pos	gene	trs_Chr	trs_start	trs_stop	region	GWAS_Cat_AD	pvalue_Nalls	GWAS_Cat_PD
rs12080565	1	223905833	LOC254312	10	10972373	11010195	PUTM	Not reported	8.21E−01	Not reported
rs16840259	1	240666644	ADAMTS9	3	64501336	64730291	SNIG	Not reported	5.11E−01	Not reported
rs1360838	1	61306961	GPR6	6	110242114	110336576	SNIG	Not reported	9.60E−01	Not reported
rs12062545	1	164684722	LPPR2	19	11466107	11476334	SNIG	Not reported	4.15E−01	Not reported
rs2601062	2	169274248	ISPD	7	16127162	16129265	PUTM	Not reported	7.61E−01	Not reported
rs17021120	2	37843019	STK32B	4	5053344	5534441	SNIG	Not reported	7.49E−01	Not reported
rs2011966	2	37844646	STK32B	4	5053344	5534441	SNIG	Not reported	6.92E−01	Not reported
rs1608518	2	168187606	SPTBN1	2	54683417	54898563	SNIG	Not reported	7.13E−01	Not reported
rs899185	2	168216090	SPTBN1	2	54683417	54898563	SNIG	Not reported	7.46E−01	Not reported
rs2617391	2	168217204	SPTBN1	2	54683417	54898563	SNIG	Not reported	7.82E−01	Not reported
rs2250619	2	168222414	SPTBN1	2	54683417	54898563	SNIG	Not reported	6.44E−01	Not reported
rs2689820	2	168226481	SPTBN1	2	54683417	54898563	SNIG	Not reported	7.17E−01	Not reported
rs1403895	2	168287130	SPTBN1	2	54683417	54898563	SNIG	Not reported	5.32E−01	Not reported
rs6729076	2	168295284	SPTBN1	2	54683417	54898563	SNIG	Not reported	6.50E−01	Not reported
rs6726976	2	168316021	SPTBN1	2	54683417	54898563	SNIG	Not reported	6.07E−01	Not reported
rs13419259	2	168322055	SPTBN1	2	54683417	54898563	SNIG	Not reported	6.53E−01	Not reported
rs899185	2	168216090	WNT5A	3	55499743	55521660	SNIG	Not reported	7.46E−01	Not reported
rs2617391	2	168217204	WNT5A	3	55499743	55521660	SNIG	Not reported	7.82E−01	Not reported
rs2250619	2	168222414	WNT5A	3	55499743	55521660	SNIG	Not reported	6.44E−01	Not reported
rs2689820	2	168226481	WNT5A	3	55499743	55521660	SNIG	Not reported	7.17E−01	Not reported
rs1403895	2	168287130	WNT5A	3	55499743	55521660	SNIG	Not reported	5.32E−01	Not reported
rs6729076	2	168295284	WNT5A	3	55499743	55521660	SNIG	Not reported	6.50E−01	Not reported
rs6726976	2	168316021	WNT5A	3	55499743	55521660	SNIG	Not reported	6.07E−01	Not reported
rs13419259	2	168322055	WNT5A	3	55499743	55521660	SNIG	Not reported	6.53E−01	Not reported
rs899185	2	168216090	GRIA2	4	158125354	158639689	SNIG	Not reported	7.46E−01	Not reported
rs2617391	2	168217204	GRIA2	4	158125354	158639689	SNIG	Not reported	7.82E−01	Not reported
rs2250619	2	168222414	GRIA2	4	158125354	158639689	SNIG	Not reported	6.44E−01	Not reported
rs2689820	2	168226481	GRIA2	4	158125354	158639689	SNIG	Not reported	7.17E−01	Not reported
rs1403895	2	168287130	GRIA2	4	158125354	158639689	SNIG	Not reported	5.32E−01	Not reported
rs6729076	2	168295284	GRIA2	4	158125354	158639689	SNIG	Not reported	6.50E−01	Not reported
rs6726976	2	168316021	GRIA2	4	158125354	158639689	SNIG	Not reported	6.07E−01	Not reported
rs13419259	2	168322055	GRIA2	4	158125354	158639689	SNIG	Not reported	6.53E−01	Not reported
rs899185	2	168216090	ELOVL6	4	110967011	111119805	SNIG	Not reported	7.46E−01	Not reported
rs2617391	2	168217204	ELOVL6	4	110967011	111119805	SNIG	Not reported	7.82E−01	Not reported
rs2250619	2	168222414	ELOVL6	4	110967011	111119805	SNIG	Not reported	6.44E−01	Not reported
rs2689820	2	168226481	ELOVL6	4	110967011	111119805	SNIG	Not reported	7.17E−01	Not reported
rs1403895	2	168287130	ELOVL6	4	110967011	111119805	SNIG	Not reported	5.32E−01	Not reported
rs6729076	2	168295284	ELOVL6	4	110967011	111119805	SNIG	Not reported	6.50E−01	Not reported
rs6726976	2	168316021	ELOVL6	4	110967011	111119805	SNIG	Not reported	6.07E−01	Not reported
rs13419259	2	168322055	ELOVL6	4	110967011	111119805	SNIG	Not reported	6.53E−01	Not reported
rs899185	2	168216090	TRIM41	5	180649519	180662790	SNIG	Not reported	7.46E−01	Not reported
rs2617391	2	168217204	TRIM41	5	180649519	180662790	SNIG	Not reported	7.82E−01	Not reported
rs2250619	2	168222414	TRIM41	5	180649519	180662790	SNIG	Not reported	6.44E−01	Not reported
rs2689820	2	168226481	TRIM41	5	180649519	180662790	SNIG	Not reported	7.17E−01	Not reported
rs1403895	2	168287130	TRIM41	5	180649519	180662790	SNIG	Not reported	5.32E−01	Not reported
rs6729076	2	168295284	TRIM41	5	180649519	180662790	SNIG	Not reported	6.50E−01	Not reported
rs6726976	2	168316021	TRIM41	5	180649519	180662790	SNIG	Not reported	6.07E−01	Not reported
rs13419259	2	168322055	TRIM41	5	180649519	180662790	SNIG	Not reported	6.53E−01	Not reported
rs899185	2	168216090	LHFPL3	7	103945394	104549001	SNIG	Not reported	7.46E−01	Not reported
rs2617391	2	168217204	LHFPL3	7	103945394	104549001	SNIG	Not reported	7.82E−01	Not reported
rs2250619	2	168222414	LHFPL3	7	103945394	104549001	SNIG	Not reported	6.44E−01	Not reported
rs2689820	2	168226481	LHFPL3	7	103945394	104549001	SNIG	Not reported	7.17E−01	Not reported
rs1403895	2	168287130	LHFPL3	7	103945394	104549001	SNIG	Not reported	5.32E−01	Not reported
rs6729076	2	168295284	LHFPL3	7	103945394	104549001	SNIG	Not reported	6.50E−01	Not reported
rs6726976	2	168316021	LHFPL3	7	103945394	104549001	SNIG	Not reported	6.07E−01	Not reported
rs13419259	2	168322055	LHFPL3	7	103945394	104549001	SNIG	Not reported	6.53E−01	Not reported
rs13032587	2	177683404	ISCA1	9	88744563	88897514	SNIG	Not reported	3.81E−01	Not reported
rs75758327	2	168115797	LRRTM3	10	68640743	68859501	SNIG	Not reported	7.00E−01	Not reported
rs1403894	2	168161260	LRRTM3	10	68640743	68859501	SNIG	Not reported	8.18E−01	Not reported
rs1540821	2	168175848	LRRTM3	10	68640743	68859501	SNIG	Not reported	3.44E−01	Not reported
rs1540820	2	168176066	LRRTM3	10	68640743	68859501	SNIG	Not reported	2.89E−01	Not reported
rs1608518	2	168187606	LRRTM3	10	68640743	68859501	SNIG	Not reported	7.13E−01	Not reported
rs1357602	2	168196591	LRRTM3	10	68640743	68859501	SNIG	Not reported	7.73E−01	Not reported
rs2140501	2	168203116	LRRTM3	10	68640743	68859501	SNIG	Not reported	5.50E−01	Not reported
rs899185	2	168216090	LRRTM3	10	68640743	68859501	SNIG	Not reported	7.46E−01	Not reported
rs2617391	2	168217204	LRRTM3	10	68640743	68859501	SNIG	Not reported	7.82E−01	Not reported
rs2250619	2	168222414	LRRTM3	10	68640743	68859501	SNIG	Not reported	6.44E−01	Not reported
rs2689820	2	168226481	LRRTM3	10	68640743	68859501	SNIG	Not reported	7.17E−01	Not reported
rs1403895	2	168287130	LRRTM3	10	68640743	68859501	SNIG	Not reported	5.32E−01	Not reported
rs6729076	2	168295284	LRRTM3	10	68640743	68859501	SNIG	Not reported	6.50E−01	Not reported
rs6726976	2	168316021	LRRTM3	10	68640743	68859501	SNIG	Not reported	6.07E−01	Not reported
rs13419259	2	168322055	LRRTM3	10	68640743	68859501	SNIG	Not reported	6.53E−01	Not reported
rs899185	2	168216090	LRRTM3	10	68860546	68861289	SNIG	Not reported	7.46E−01	Not reported
rs2617391	2	168217204	LRRTM3	10	68860546	68861289	SNIG	Not reported	7.82E−01	Not reported
rs2250619	2	168222414	LRRTM3	10	68860546	68861289	SNIG	Not reported	6.44E−01	Not reported
rs2689820	2	168226481	LRRTM3	10	68860546	68861289	SNIG	Not reported	7.17E−01	Not reported
rs1403895	2	168287130	LRRTM3	10	68860546	68861289	SNIG	Not reported	5.32E−01	Not reported
rs6729076	2	168295284	LRRTM3	10	68860546	68861289	SNIG	Not reported	6.50E−01	Not reported
rs6726976	2	168316021	LRRTM3	10	68860546	68861289	SNIG	Not reported	6.07E−01	Not reported
rs13419259	2	168322055	LRRTM3	10	68860546	68861289	SNIG	Not reported	6.53E−01	Not reported
rs75758327	2	168115797	PCDH15	10	55566062	57388232	SNIG	Not reported	7.00E−01	Not reported
rs1403894	2	168161260	PCDH15	10	55566062	57388232	SNIG	Not reported	8.18E−01	Not reported
rs1540821	2	168175848	PCDH15	10	55566062	57388232	SNIG	Not reported	3.44E−01	Not reported
rs1540820	2	168176066	PCDH15	10	55566062	57388232	SNIG	Not reported	2.89E−01	Not reported
rs1608518	2	168187606	PCDH15	10	55566062	57388232	SNIG	Not reported	7.13E−01	Not reported
rs2140501	2	168203116	PCDH15	10	55566062	57388232	SNIG	Not reported	5.50E−01	Not reported
rs899185	2	168216090	PCDH15	10	55566062	57388232	SNIG	Not reported	7.46E−01	Not reported
rs2617391	2	168217204	PCDH15	10	55566062	57388232	SNIG	Not reported	7.82E−01	Not reported
rs2250619	2	168222414	PCDH15	10	55566062	57388232	SNIG	Not reported	6.44E−01	Not reported
rs2689820	2	168226481	PCDH15	10	55566062	57388232	SNIG	Not reported	7.17E−01	Not reported
rs1403895	2	168287130	PCDH15	10	55566062	57388232	SNIG	Not reported	5.32E−01	Not reported
rs6729076	2	168295284	PCDH15	10	55566062	57388232	SNIG	Not reported	6.50E−01	Not reported
rs6726976	2	168316021	PCDH15	10	55566062	57388232	SNIG	Not reported	6.07E−01	Not reported
rs13419259	2	168322055	PCDH15	10	55566062	57388232	SNIG	Not reported	6.53E−01	Not reported
rs899185	2	168216090	GFRA1	10	117709300	118033795	SNIG	Not reported	7.46E−01	Not reported
rs2617391	2	168217204	GFRA1	10	117709300	118033795	SNIG	Not reported	7.82E−01	Not reported
rs2250619	2	168222414	GFRA1	10	117709300	118033795	SNIG	Not reported	6.44E−01	Not reported
rs2689820	2	168226481	GFRA1	10	117709300	118033795	SNIG	Not reported	7.17E−01	Not reported
rs1403895	2	168287130	GFRA1	10	117709300	118033795	SNIG	Not reported	5.32E−01	Not reported
rs6729076	2	168295284	GFRA1	10	117709300	118033795	SNIG	Not reported	6.50E−01	Not reported
rs6726976	2	168316021	GFRA1	10	117709300	118033795	SNIG	Not reported	6.07E−01	Not reported
rs13419259	2	168322055	GFRA1	10	117709300	118033795	SNIG	Not reported	6.53E−01	Not reported
rs4387746	2	168330745	GFRA1	10	117709300	118033795	SNIG	Not reported	8.16E−01	Not reported
rs899185	2	168216090	NCAM1	11	112831988	113149148	SNIG	Not reported	7.46E−01	Not reported
rs2617391	2	168217204	NCAM1	11	112831988	113149148	SNIG	Not reported	7.82E−01	Not reported
rs2250619	2	168222414	NCAM1	11	112831988	113149148	SNIG	Not reported	6.44E−01	Not reported
rs2689820	2	168226481	NCAM1	11	112831988	113149148	SNIG	Not reported	7.17E−01	Not reported
rs1403895	2	168287130	NCAM1	11	112831988	113149148	SNIG	Not reported	5.32E−01	Not reported
rs6729076	2	168295284	NCAM1	11	112831988	113149148	SNIG	Not reported	6.50E−01	Not reported
rs6726976	2	168316021	NCAM1	11	112831988	113149148	SNIG	Not reported	6.07E−01	Not reported
rs13419259	2	168322055	NCAM1	11	112831988	113149148	SNIG	Not reported	6.53E−01	Not reported
rs4387746	2	168330745	NCAM1	11	112831988	113149148	SNIG	Not reported	8.16E−01	Not reported
rs13032587	2	177683404	DENND5B	12	31535162	31743975	SNIG	Not reported	3.81E−01	Not reported
rs899185	2	168216090	ITM2B	13	48801030	48837313	SNIG	Not reported	7.46E−01	Not reported
rs2617391	2	168217204	ITM2B	13	48801030	48837313	SNIG	Not reported	7.82E−01	Not reported
rs2250619	2	168222414	ITM2B	13	48801030	48837313	SNIG	Not reported	6.44E−01	Not reported
rs2689820	2	168226481	ITM2B	13	48801030	48837313	SNIG	Not reported	7.17E−01	Not reported
rs1403895	2	168287130	ITM2B	13	48801030	48837313	SNIG	Not reported	5.32E−01	Not reported
rs6729076	2	168295284	ITM2B	13	48801030	48837313	SNIG	Not reported	6.50E−01	Not reported
rs6726976	2	168316021	ITM2B	13	48801030	48837313	SNIG	Not reported	6.07E−01	Not reported
rs13419259	2	168322055	ITM2B	13	48801030	48837313	SNIG	Not reported	6.53E−01	Not reported
rs899185	2	168216090	KIF1C	17	4901243	4935905	SNIG	Not reported	7.46E−01	Not reported
rs2617391	2	168217204	KIF1C	17	4901243	4935905	SNIG	Not reported	7.82E−01	Not reported
rs2250619	2	168222414	KIF1C	17	4901243	4935905	SNIG	Not reported	6.44E−01	Not reported
rs2689820	2	168226481	KIF1C	17	4901243	4935905	SNIG	Not reported	7.17E−01	Not reported
rs1403895	2	168287130	KIF1C	17	4901243	4935905	SNIG	Not reported	5.32E−01	Not reported
rs6729076	2	168295284	KIF1C	17	4901243	4935905	SNIG	Not reported	6.50E−01	Not reported
rs6726976	2	168316021	KIF1C	17	4901243	4935905	SNIG	Not reported	6.07E−01	Not reported
rs13419259	2	168322055	KIF1C	17	4901243	4935905	SNIG	Not reported	6.53E−01	Not reported
rs899185	2	168216090	CYTSB	17	19912154	20218052	SNIG	Not reported	7.46E−01	Not reported
rs2617391	2	168217204	CYTSB	17	19912154	20218052	SNIG	Not reported	7.82E−01	Not reported
rs2250619	2	168222414	CYTSB	17	19912154	20218052	SNIG	Not reported	6.44E−01	Not reported
rs2689820	2	168226481	CYTSB	17	19912154	20218052	SNIG	Not reported	7.17E−01	Not reported
rs1403895	2	168287130	CYTSB	17	19912154	20218052	SNIG	Not reported	5.32E−01	Not reported
rs6729076	2	168295284	CYTSB	17	19912154	20218052	SNIG	Not reported	6.50E−01	Not reported
rs6726976	2	168316021	CYTSB	17	19912154	20218052	SNIG	Not reported	6.07E−01	Not reported
rs13419259	2	168322055	CYTSB	17	19912154	20218052	SNIG	Not reported	6.53E−01	Not reported
rs899185	2	168216090	SEZ6L	22	26565450	26818910	SNIG	Not reported	7.46E−01	Not reported
rs2617391	2	168217204	SEZ6L	22	26565450	26818910	SNIG	Not reported	7.82E−01	Not reported
rs2250619	2	168222414	SEZ6L	22	26565450	26818910	SNIG	Not reported	6.44E−01	Not reported
rs2689820	2	168226481	SEZ6L	22	26565450	26818910	SNIG	Not reported	7.17E−01	Not reported
rs1403895	2	168287130	SEZ6L	22	26565450	26818910	SNIG	Not reported	5.32E−01	Not reported
rs6729076	2	168295284	SEZ6L	22	26565450	26818910	SNIG	Not reported	6.50E−01	Not reported
rs6726976	2	168316021	SEZ6L	22	26565450	26818910	SNIG	Not reported	6.07E−01	Not reported
rs13419259	2	168322055	SEZ6L	22	26565450	26818910	SNIG	Not reported	6.53E−01	Not reported
rs899185	2	168216090	TRO	X	54925686	54957857	SNIG	Not reported	7.46E−01	Not reported
rs2617391	2	168217204	TRO	X	54925686	54957857	SNIG	Not reported	7.82E−01	Not reported
rs2250619	2	168222414	TRO	X	54925686	54957857	SNIG	Not reported	6.44E−01	Not reported
rs2689820	2	168226481	TRO	X	54925686	54957857	SNIG	Not reported	7.17E−01	Not reported
rs1403895	2	168287130	TRO	X	54925686	54957857	SNIG	Not reported	5.32E−01	Not reported
rs6729076	2	168295284	TRO	X	54925686	54957857	SNIG	Not reported	6.50E−01	Not reported
rs6726976	2	168316021	TRO	X	54925686	54957857	SNIG	Not reported	6.07E−01	Not reported
rs13419259	2	168322055	TRO	X	54925686	54957857	SNIG	Not reported	6.53E−01	Not reported
rs10510809	3	59439053	HOMER1	5	78668619	78809934	PUTM	Not reported	5.39E−01	Not reported
rs2366700	3	59443422	HOMER1	5	78668619	78809934	PUTM	Not reported	5.86E−01	Not reported
rs9883195	3	59460320	HOMER1	5	78668619	78809934	PUTM	Not reported	7.01E−01	Not reported
rs9863830	3	133411224	KIAA0040	1	175126127	175162240	SNIG	Not reported	2.96E−01	Not reported
rs13072780	3	133290960	ADAMTS9	3	64501336	64730291	SNIG	Not reported	2.13E−01	Not reported
rs10049041	3	133291621	ADAMTS9	3	64501336	64730291	SNIG	Not reported	2.22E−01	Not reported
rs12695595	3	133303289	ADAMTS9	3	64501336	64730291	SNIG	Not reported	2.09E−01	Not reported
rs1051772	3	133327457	ADAMTS9	3	64501336	64730291	SNIG	Not reported	3.58E−01	Not reported
rs71317417	3	133331230	ADAMTS9	3	64501336	64730291	SNIG	Not reported	1.18E−01	Not reported
rs1348574	3	133375081	ADAMTS9	3	64501336	64730291	SNIG	Not reported	1.19E−01	Not reported
rs9290095	3	161496202	C9orf43	9	116172357	116191964	SNIG	Not reported	4.80E−01	Not reported
rs10936238	3	161499569	C9orf43	9	116172357	116191964	SNIG	Not reported	5.05E−01	Not reported
rs11735575	4	84823287	ADCY3	2	25042041	25152258	PUTM	Not reported	3.08E−01	Not reported
rs11735575	4	84823287	SLC25A13	7	95749536	95951419	PUTM	Not reported	3.08E−01	Not reported
rs6833842	4	34600316	FAM66B	8	11973769	12037647	PUTM	Not reported	8.79E−01	Not reported
rs6843865	4	34662282	FAM66B	8	11973769	12037647	PUTM	Not reported	9.52E−01	Not reported
rs11735575	4	84823287	FNTB	14	65450485	65529359	PUTM	Not reported	3.08E−01	Not reported
rs10488947	4	10503752	HERC2P2	15	20587578	20711419	PUTM	Not reported	6.18E−01	Not reported
rs11735575	4	84823287	CNTNAP4	16	76311186	76650146	PUTM	Not reported	3.08E−01	Not reported
rs1996374	4	148413483	MYL9	20	35169897	35178209	PUTM	Not reported	2.75E−01	Not reported
rs236768	4	103075983	PCDH1	5	141229980	141262851	SNIG	Not reported	8.21E−01	Not reported
rs236770	4	103079845	PCDH1	5	141229980	141262851	SNIG	Not reported	7.56E−01	Not reported
rs17838972	4	71357077	MDC1	6	30667591	30685646	SNIG	Not reported	9.85E−01	Not reported
rs28674149	4	71384488	MDC1	6	30667591	30685646	SNIG	Not reported	9.16E−01	Not reported
rs17054460	4	169601223	RER1	1	2323242	2336861	SNIG	Not reported	3.20E−01	Not reported
rs4692947	4	169605918	RER1	1	2323242	2336861	SNIG	Not reported	3.26E−01	Not reported
rs62333891	4	169606649	RER1	1	2323242	2336861	SNIG	Not reported	3.20E−01	Not reported
rs17054460	4	169601223	HECTD3	1	45466711	45476986	SNIG	Not reported	3.20E−01	Not reported
rs4692947	4	169605918	HECTD3	1	45466711	45476986	SNIG	Not reported	3.26E−01	Not reported
rs62333891	4	169606649	HECTD3	1	45466711	45476986	SNIG	Not reported	3.20E−01	Not reported
rs17054460	4	169601223	LRRC41	1	46726888	46797829	SNIG	Not reported	3.20E−01	Not reported
rs4692947	4	169605918	LRRC41	1	46726888	46797829	SNIG	Not reported	3.26E−01	Not reported
rs62333891	4	169606649	LRRC41	1	46726888	46797829	SNIG	Not reported	3.20E−01	Not reported
rs17054460	4	169601223	TTLL3	3	9834245	9896812	SNIG	Not reported	3.20E−01	Not reported
rs4692947	4	169605918	TTLL3	3	9834245	9896812	SNIG	Not reported	3.26E−01	Not reported
rs62333891	4	169606649	TTLL3	3	9834245	9896812	SNIG	Not reported	3.20E−01	Not reported
rs17054460	4	169601223	LRPAP1	4	3508113	3534139	SNIG	Not reported	3.20E−01	Not reported
rs4692947	4	169605918	LRPAP1	4	3508113	3534139	SNIG	Not reported	3.26E−01	Not reported
rs62333891	4	169606649	LRPAP1	4	3508113	3534139	SNIG	Not reported	3.20E−01	Not reported
rs17054460	4	169601223	SQSTM1	5	179233408	179266247	SNIG	Not reported	3.20E−01	Not reported
rs4692947	4	169605918	SQSTM1	5	179233408	179266247	SNIG	Not reported	3.26E−01	Not reported
rs62333891	4	169606649	SQSTM1	5	179233408	179266247	SNIG	Not reported	3.20E−01	Not reported
rs17054460	4	169601223	GIGYF1	7	100277275	100277793	SNIG	Not reported	3.20E−01	Not reported
rs4692947	4	169605918	GIGYF1	7	100277275	100277793	SNIG	Not reported	3.26E−01	Not reported
rs62333891	4	169606649	GIGYF1	7	100277275	100277793	SNIG	Not reported	3.20E−01	Not reported
rs17054460	4	169601223	KBTBD2	7	32907793	32933723	SNIG	Not reported	3.20E−01	Not reported
rs4692947	4	169605918	KBTBD2	7	32907793	32933723	SNIG	Not reported	3.26E−01	Not reported
rs62333891	4	169606649	KBTBD2	7	32907793	32933723	SNIG	Not reported	3.20E−01	Not reported
rs17054460	4	169601223	LRCH4	7	100167690	100183891	SNIG	Not reported	3.20E−01	Not reported
rs4692947	4	169605918	LRCH4	7	100167690	100183891	SNIG	Not reported	3.26E−01	Not reported
rs62333891	4	169606649	LRCH4	7	100167690	100183891	SNIG	Not reported	3.20E−01	Not reported
rs17054460	4	169601223	SEC16A	9	139370703	139370980	SNIG	Not reported	3.20E−01	Not reported
rs4692947	4	169605918	SEC16A	9	139370703	139370980	SNIG	Not reported	3.26E−01	Not reported
rs62333891	4	169606649	SEC16A	9	139370703	139370980	SNIG	Not reported	3.20E−01	Not reported
rs17054460	4	169601223	TSSC4	11	2421728	2425086	SNIG	Not reported	3.20E−01	Not reported
rs4692947	4	169605918	TSSC4	11	2421728	2425086	SNIG	Not reported	3.26E−01	Not reported
rs62333891	4	169606649	TSSC4	11	2421728	2425086	SNIG	Not reported	3.20E−01	Not reported
rs17054460	4	169601223	B3GAT3	11	62382779	62389616	SNIG	Not reported	3.20E−01	Not reported
rs4692947	4	169605918	B3GAT3	11	62382779	62389616	SNIG	Not reported	3.26E−01	Not reported
rs62333891	4	169606649	B3GAT3	11	62382779	62389616	SNIG	Not reported	3.20E−01	Not reported
rs17054460	4	169601223	OGFOD2	12	123459247	123463980	SNIG	Not reported	3.20E−01	Not reported
rs4692947	4	169605918	OGFOD2	12	123459247	123463980	SNIG	Not reported	3.26E−01	Not reported
rs62333891	4	169606649	OGFOD2	12	123459247	123463980	SNIG	Not reported	3.20E−01	Not reported
rs17054460	4	169601223	THTPA	14	23980989	24030089	SNIG	Not reported	3.20E−01	Not reported
rs4692947	4	169605918	THTPA	14	23980989	24030089	SNIG	Not reported	3.26E−01	Not reported
rs62333891	4	169606649	THTPA	14	23980989	24030089	SNIG	Not reported	3.20E−01	Not reported
rs17054460	4	169601223	CLCN7	16	1494936	1525571	SNIG	Not reported	3.20E−01	Not reported
rs4692947	4	169605918	CLCN7	16	1494936	1525571	SNIG	Not reported	3.26E−01	Not reported
rs62333891	4	169606649	CLCN7	16	1494936	1525571	SNIG	Not reported	3.20E−01	Not reported
rs17054460	4	169601223	TRAP1	16	3700637	3773274	SNIG	Not reported	3.20E−01	Not reported
rs4692947	4	169605918	TRAP1	16	3700637	3773274	SNIG	Not reported	3.26E−01	Not reported
rs62333891	4	169606649	TRAP1	16	3700637	3773274	SNIG	Not reported	3.20E−01	Not reported
rs17054460	4	169601223	GTF3C1	16	27471936	27561274	SNIG	Not reported	3.20E−01	Not reported
rs4692947	4	169605918	GTF3C1	16	27471936	27561274	SNIG	Not reported	3.26E−01	Not reported
rs62333891	4	169606649	GTF3C1	16	27471936	27561274	SNIG	Not reported	3.20E−01	Not reported
rs17054460	4	169601223	SLC38A7	16	58699016	58718620	SNIG	Not reported	3.20E−01	Not reported
rs4692947	4	169605918	SLC38A7	16	58699016	58718620	SNIG	Not reported	3.26E−01	Not reported
rs62333891	4	169606649	SLC38A7	16	58699016	58718620	SNIG	Not reported	3.20E−01	Not reported
rs17054460	4	169601223	CENPT	16	67861952	67881351	SNIG	Not reported	3.20E−01	Not reported
rs4692947	4	169605918	CENPT	16	67861952	67881351	SNIG	Not reported	3.26E−01	Not reported
rs62333891	4	169606649	CENPT	16	67861952	67881351	SNIG	Not reported	3.20E−01	Not reported
rs17054460	4	169601223	ACADVL	17	7118434	7128948	SNIG	Not reported	3.20E−01	Not reported
rs4692947	4	169605918	ACADVL	17	7118434	7128948	SNIG	Not reported	3.26E−01	Not reported
rs62333891	4	169606649	ACADVL	17	7118434	7128948	SNIG	Not reported	3.20E−01	Not reported
rs17054460	4	169601223	G6PC3	17	42148118	42153681	SNIG	Not reported	3.20E−01	Not reported
rs4692947	4	169605918	G6PC3	17	42148118	42153681	SNIG	Not reported	3.26E−01	Not reported
rs62333891	4	169606649	G6PC3	17	42148118	42153681	SNIG	Not reported	3.20E−01	Not reported
rs6832582	4	169599633	FN3K	17	80693468	80709073	SNIG	Not reported	2.63E−01	Not reported
rs17054460	4	169601223	FN3K	17	80693468	80709073	SNIG	Not reported	3.20E−01	Not reported
rs4692947	4	169605918	FN3K	17	80693468	80709073	SNIG	Not reported	3.26E−01	Not reported
rs62333891	4	169606649	FN3K	17	80693468	80709073	SNIG	Not reported	3.20E−01	Not reported
rs17054460	4	169601223	TBCD	17	80709925	80907425	SNIG	Not reported	3.20E−01	Not reported
rs4692947	4	169605918	TBCD	17	80709925	80907425	SNIG	Not reported	3.26E−01	Not reported
rs62333891	4	169606649	TBCD	17	80709925	80907425	SNIG	Not reported	3.20E−01	Not reported
rs17054460	4	169601223	PFN1	17	4848955	4852609	SNIG	Not reported	3.20E−01	Not reported
rs4692947	4	169605918	PFN1	17	4848955	4852609	SNIG	Not reported	3.26E−01	Not reported
rs62333891	4	169606649	PFN1	17	4848955	4852609	SNIG	Not reported	3.20E−01	Not reported
rs17054460	4	169601223	GGA3	17	73232696	73257865	SNIG	Not reported	3.20E−01	Not reported
rs4692947	4	169605918	GGA3	17	73232696	73257865	SNIG	Not reported	3.26E−01	Not reported
rs62333891	4	169606649	GGA3	17	73232696	73257865	SNIG	Not reported	3.20E−01	Not reported
rs17054460	4	169601223	SGSH	17	78174050	78194193	SNIG	Not reported	3.20E−01	Not reported
rs4692947	4	169605918	SGSH	17	78174050	78194193	SNIG	Not reported	3.26E−01	Not reported
rs62333891	4	169606649	SGSH	17	78174050	78194193	SNIG	Not reported	3.20E−01	Not reported
rs17054460	4	169601223	MAP1S	19	17830312	17845295	SNIG	Not reported	3.20E−01	Not reported
rs4692947	4	169605918	MAP1S	19	17830312	17845295	SNIG	Not reported	3.26E−01	Not reported
rs62333891	4	169606649	MAP1S	19	17830312	17845295	SNIG	Not reported	3.20E−01	Not reported
rs17054460	4	169601223	UPF1	19	18942757	18979030	SNIG	Not reported	3.20E−01	Not reported
rs4692947	4	169605918	UPF1	19	18942757	18979030	SNIG	Not reported	3.26E−01	Not reported
rs62333891	4	169606649	UPF1	19	18942757	18979030	SNIG	Not reported	3.20E−01	Not reported
rs17054460	4	169601223	EGLN2	19	41304921	41332604	SNIG	Not reported	3.20E−01	Not reported
rs4692947	4	169605918	EGLN2	19	41304921	41332604	SNIG	Not reported	3.26E−01	Not reported
rs62333891	4	169606649	EGLN2	19	41304921	41332604	SNIG	Not reported	3.20E−01	Not reported
rs17054460	4	169601223	PRR12	19	50099562	50100046	SNIG	Not reported	3.20E−01	Not reported
rs4692947	4	169605918	PRR12	19	50099562	50100046	SNIG	Not reported	3.26E−01	Not reported
rs62333891	4	169606649	PRR12	19	50099562	50100046	SNIG	Not reported	3.20E−01	Not reported
rs17054460	4	169601223	PTOV1	19	50349518	50363966	SNIG	Not reported	3.20E−01	Not reported
rs4692947	4	169605918	PTOV1	19	50349518	50363966	SNIG	Not reported	3.26E−01	Not reported
rs62333891	4	169606649	PTOV1	19	50349518	50363966	SNIG	Not reported	3.20E−01	Not reported
rs17054460	4	169601223	NR2F6	19	17342701	17356708	SNIG	Not reported	3.20E−01	Not reported
rs4692947	4	169605918	NR2F6	19	17342701	17356708	SNIG	Not reported	3.26E−01	Not reported
rs62333891	4	169606649	NR2F6	19	17342701	17356708	SNIG	Not reported	3.20E−01	Not reported
rs17054460	4	169601223	LZTR1	22	21336325	21353304	SNIG	Not reported	3.20E−01	Not reported
rs4692947	4	169605918	LZTR1	22	21336325	21353304	SNIG	Not reported	3.26E−01	Not reported
rs62333891	4	169606649	LZTR1	22	21336325	21353304	SNIG	Not reported	3.20E−01	Not reported
rs17054460	4	169601223	PPIL2	22	22000759	22055603	SNIG	Not reported	3.20E−01	Not reported
rs4692947	4	169605918	PPIL2	22	22000759	22055603	SNIG	Not reported	3.26E−01	Not reported
rs62333891	4	169606649	PPIL2	22	22000759	22055603	SNIG	Not reported	3.20E−01	Not reported
rs17054460	4	169601223	TRMU	22	46731318	46753232	SNIG	Not reported	3.20E−01	Not reported
rs4692947	4	169605918	TRMU	22	46731318	46753232	SNIG	Not reported	3.26E−01	Not reported
rs62333891	4	169606649	TRMU	22	46731318	46753232	SNIG	Not reported	3.20E−01	Not reported
rs17054460	4	169601223	TRMT2A	22	20098499	20112868	SNIG	Not reported	3.20E−01	Not reported
rs4692947	4	169605918	TRMT2A	22	20098499	20112868	SNIG	Not reported	3.26E−01	Not reported
rs62333891	4	169606649	TRMT2A	22	20098499	20112868	SNIG	Not reported	3.20E−01	Not reported
rs17054460	4	169601223	UBA1	X	47049976	47074811	SNIG	Not reported	3.20E−01	Not reported
rs4692947	4	169605918	UBA1	X	47049976	47074811	SNIG	Not reported	3.26E−01	Not reported
rs62333891	4	169606649	UBA1	X	47049976	47074811	SNIG	Not reported	3.20E−01	Not reported
rs2292434	4	189371244	APEX2	X	55026084	55034286	SNIG	Not reported	5.14E−01	Not reported
rs34594012	5	177987740	GPM6A	4	176554108	176923766	PUTM	Not reported	3.07E−01	Not reported
rs1431948	5	121112267	ACN9	7	96721942	96974660	PUTM	Not reported	6.73E−01	Not reported
rs73292425	5	121131929	ACN9	7	96721942	96974660	PUTM	Not reported	7.27E−01	Not reported
rs34594012	5	177987740	LGI1	10	95514151	95560002	PUTM	Not reported	3.07E−01	Not reported
rs11133754	5	13624329	ASB13	10	5677457	5726888	SNIG	Not reported	2.87E−01	Not reported
rs7759754	6	145130578	STARD4	5	110831737	110848615	PUTM	Not reported	4.02E−01	Not reported
rs9483632	6	134259753	C5orf53	5	139505530	139508957	SNIG	Not reported	9.44E−01	Not reported
rs10484728	6	116011942	C10orf134	10	118503225	118671282	SNIG	Not reported	8.89E−01	Not reported
rs12525228	6	116040072	C10orf134	10	118503225	118671282	SNIG	Not reported	8.44E−01	Not reported
rs79074797	6	111589764	BRMS1	11	66104817	66112708	SNIG	Not reported	3.09E−01	Not reported
rs80248759	6	111613021	BRMS1	11	66104817	66112708	SNIG	Not reported	3.74E−01	Not reported
rs79074797	6	111589764	UBL5	19	9932207	9940787	SNIG	Not reported	3.09E−01	Not reported
rs80248759	6	111613021	UBL5	19	9932207	9940787	SNIG	Not reported	3.74E−01	Not reported
rs79074797	6	111589764	FKBP8	19	18642580	18654848	SNIG	Not reported	3.09E−01	Not reported
rs80248759	6	111613021	FKBP8	19	18642580	18654848	SNIG	Not reported	3.74E−01	Not reported
rs9394699	6	40449290	ZNF630	X	47917587	47931059	SNIG	Not reported	1.87E−01	Not reported
rs7804782	7	12558799	QARS	3	49133372	49142542	PUTM	Not reported	1.67E−01	Not reported
rs9656412	7	132422248	NKAIN1	1	31652029	31697450	SNIG	Not reported	4.14E−01	Not reported
rs1581568	7	13656560	CDNF	10	14861263	14881101	SNIG	Not reported	6.09E−01	Not reported
rs1581567	7	13656650	CDNF	10	14861263	14881101	SNIG	Not reported	4.89E−01	Not reported
rs7823489	8	5106677	C19orf2	19	30414561	30507515	PUTM	Not reported	1.84E−01	Not reported
rs7823489	8	5106677	LOC647979	20	34633555	34638877	PUTM	Not reported	1.84E−01	Not reported
rs7823489	8	5106677	FMR1	X	146993406	147032637	PUTM	Not reported	1.84E−01	Not reported
rs72653977	8	57015013	UBN2	7	138940653	138944114	SNIG	Not reported	4.52E−01	Not reported
rs36107033	8	37556987	LOC374890	19	22798359	22806763	SNIG	Not reported	4.52E−01	Not reported
rs10103391	8	132521299	EDN3	20	57865163	57941017	SNIG	Not reported	6.28E−01	Not reported
rs79977819	9	4284391	NBPF15	1	146032553	147400375	PUTM	Not reported	1.51E−02	Not reported
rs74742357	9	4286816	NBPF15	1	146032553	147400375	PUTM	Not reported	2.91E−02	Not reported
rs16921031	9	4286898	NBPF15	1	146032553	147400375	PUTM	Not reported	2.38E−02	Not reported
rs16921043	9	4288476	NBPF15	1	146032553	147400375	PUTM	Not reported	3.46E−02	Not reported
rs117445974	9	4288739	NBPF15	1	146032553	147400375	PUTM	Not reported	3.17E−02	Not reported
rs16921047	9	4288930	NBPF15	1	146032553	147400375	PUTM	Not reported	4.39E−02	Not reported
rs17717221	9	4292496	NBPF15	1	146032553	147400375	PUTM	Not reported	1.96E−02	Not reported
rs10975965	9	7004340	SH3BGRL	X	80457443	80554038	PUTM	Not reported	9.79E−01	Not reported
rs10813262	9	30484037	KCNQ5	6	73331809	73905682	SNIG	Not reported	7.48E−01	Not reported
rs4532690	9	30493749	KCNQ5	6	73331809	73905682	SNIG	Not reported	7.38E−01	Not reported
rs4878432	9	30500252	KCNQ5	6	73331809	73905682	SNIG	Not reported	7.57E−01	Not reported
rs1463984	9	108427062	GIGYF1	7	100276189	100287061	SNIG	Not reported	3.66E−01	Not reported
rs45449193	9	139748483	FUS	16	31185894	31203502	SNIG	Not reported	5.11E−01	Not reported
rs11002001	10	54186172	MPZ	1	161274528	161279725	SNIG	Not reported	8.24E−01	Not reported
rs11002001	10	54186172	RDH10	8	74207265	74322499	SNIG	Not reported	8.24E−01	Not reported
rs11002001	10	54186172	ACTN1	14	69340064	69446062	SNIG	Not reported	8.24E−01	Not reported
rs1670137	10	67727223	SYCE2	19	13009852	13030176	SNIG	Not reported	2.91E−01	Not reported
rs2707100	11	19935832	C14orf57	14	72456191	72576129	SNIG	Not reported	5.77E−01	Not reported
rs923171	11	12069248	HEXA	15	72613683	72668760	SNIG	Not reported	6.93E−02	Not reported
rs11608068	11	12070331	HEXA	15	72613683	72668760	SNIG	Not reported	8.99E−02	Not reported
rs923171	11	12069248	ZACN	17	74075273	74078885	SNIG	Not reported	6.93E−02	Not reported
rs11608068	11	12070331	ZACN	17	74075273	74078885	SNIG	Not reported	8.99E−02	Not reported
rs17125418	12	51530962	NBPF15	1	146032553	147400375	PUTM	Not reported	7.95E−01	Not reported
rs11169718	12	51533176	NBPF15	1	146032553	147400375	PUTM	Not reported	9.84E−01	Not reported
rs2286007	12	971291	ZDHHC8P1	22	23732793	23794207	SNIG	Not reported	7.11E−01	Not reported
rs888016	12	96524773	CLPB	11	71962014	72145689	SNIG	Not reported	2.77E−02	Not reported
rs888016	12	96524773	COBRA1	9	140149735	140166674	SNIG	Not reported	2.77E−02	Not reported
rs17090354	13	74277200	ZNF630	X	47917587	47931059	PUTM	Not reported	7.16E−01	Not reported
rs9561952	13	96427626	DFFB	1	3772468	3801990	SNIG	Not reported	3.50E−02	Not reported
rs7318664	13	76043991	TMEM222	1	27648634	27663052	SNIG	Not reported	9.85E−01	Not reported
rs9561952	13	96427626	SLC44A5	1	75667817	76076791	SNIG	Not reported	3.50E−02	Not reported
rs9561952	13	96427626	LOC400940	2	6122120	6126503	SNIG	Not reported	3.50E−02	Not reported
rs9561952	13	96427626	GTPBP8	3	112709785	112720314	SNIG	Not reported	3.50E−02	Not reported
rs9561952	13	96427626	CNTN3	3	74260038	74580481	SNIG	Not reported	3.50E−02	Not reported
rs9561952	13	96427626	PRDM8	4	81105000	81127127	SNIG	Not reported	3.50E−02	Not reported
rs9561952	13	96427626	C6orf89	6	36839512	36896735	SNIG	Not reported	3.50E−02	Not reported
rs9561952	13	96427626	COL9A1	6	70906426	71038692	SNIG	Not reported	3.50E−02	Not reported
rs7318664	13	76043991	POR	7	75544391	75616140	SNIG	Not reported	9.85E−01	Not reported
rs7318664	13	76043991	ADAP1	7	936564	994307	SNIG	Not reported	9.85E−01	Not reported
rs7318664	13	76043991	INTS1	7	1509153	1578668	SNIG	Not reported	9.85E−01	Not reported
rs9561952	13	96427626	NIPAL2	8	99202063	99306659	SNIG	Not reported	3.50E−02	Not reported
rs7318664	13	76043991	FBXW5	9	139834890	139839186	SNIG	Not reported	9.85E−01	Not reported
rs9561952	13	96427626	LRRTM3	10	68640743	68859501	SNIG	Not reported	3.50E−02	Not reported
rs9561952	13	96427626	PCDH15	10	55566062	57388232	SNIG	Not reported	3.50E−02	Not reported
rs7318664	13	76043991	C10orf125	10	135158147	135171511	SNIG	Not reported	9.85E−01	Not reported
rs7318664	13	76043991	TSSC4	11	2421728	2425086	SNIG	Not reported	9.85E−01	Not reported
rs9561952	13	96427626	CREB3L1	11	46299209	46342962	SNIG	Not reported	3.50E−02	Not reported
rs7318664	13	76043991	SCYL1	11	65292394	65306172	SNIG	Not reported	9.85E−01	Not reported
rs7318664	13	76043991	USP35	11	77899868	77925749	SNIG	Not reported	9.85E−01	Not reported
rs7318664	13	76043991	CTSD	11	1756041	1785783	SNIG	Not reported	9.85E−01	Not reported
rs7318664	13	76043991	NUDT8	11	67395417	67397526	SNIG	Not reported	9.85E−01	Not reported
rs7318664	13	76043991	LOC100288346	11	113140900	113141218	SNIG	Not reported	9.85E−01	Not reported
rs9561952	13	96427626	OPCML	11	132278376	133402800	SNIG	Not reported	3.50E−02	Not reported
rs9561952	13	96427626	PCDH20	13	61860381	62734963	SNIG	Not reported	3.50E−02	Not reported
rs9561952	13	96427626	SLITRK1	13	84451051	84456528	SNIG	Not reported	3.50E−02	Not reported
rs7318664	13	76043991	PIGQ	16	619970	634090	SNIG	Not reported	9.85E−01	Not reported
rs7318664	13	76043991	SPNS1	16	28985662	28995858	SNIG	Not reported	9.85E−01	Not reported
rs7318664	13	76043991	CLCN7	16	1494936	1525571	SNIG	Not reported	9.85E−01	Not reported
rs7318664	13	76043991	SLC38A7	16	58699016	58718620	SNIG	Not reported	9.85E−01	Not reported
rs7318664	13	76043991	ACADVL	17	7118434	7128948	SNIG	Not reported	9.85E−01	Not reported
rs7318664	13	76043991	GGA3	17	73232696	73257865	SNIG	Not reported	9.85E−01	Not reported
rs7318664	13	76043991	FOXK2	17	80560467	80562457	SNIG	Not reported	9.85E−01	Not reported
rs9561952	13	96427626	KIAA0802	18	8705183	8870242	SNIG	Not reported	3.50E−02	Not reported
rs7318664	13	76043991	NCLN	19	3172462	3219597	SNIG	Not reported	9.85E−01	Not reported
rs9561952	13	96427626	ATCAY	19	3866446	3928080	SNIG	Not reported	3.50E−02	Not reported
rs7318664	13	76043991	LPPR2	19	11466107	11476334	SNIG	Not reported	9.85E−01	Not reported
rs7318664	13	76043991	NACC1	19	13229091	13251956	SNIG	Not reported	9.85E−01	Not reported
rs7318664	13	76043991	C19orf60	19	18699555	18703137	SNIG	Not reported	9.85E−01	Not reported
rs7318664	13	76043991	SCAF1	19	50145392	50161859	SNIG	Not reported	9.85E−01	Not reported
rs7318664	13	76043991	DACT3	19	47150873	47164297	SNIG	Not reported	9.85E−01	Not reported
rs9561952	13	96427626	MYT1	20	62781959	62873606	SNIG	Not reported	3.50E−02	Not reported
rs7318664	13	76043991	XRCC6	22	42016313	42060026	SNIG	Not reported	9.85E−01	Not reported
rs7318664	13	76043991	TRMT2A	22	20098499	20112868	SNIG	Not reported	9.85E−01	Not reported
rs9561952	13	96427626	DCX	X	110537018	110657756	SNIG	Not reported	3.50E−02	Not reported
rs11845904	14	45404328	CHRM3	1	239549875	240074741	SNIG	Not reported	6.38E−01	Not reported
rs17115703	14	45416918	CHRM3	1	239549875	240074741	SNIG	Not reported	9.42E−01	Not reported
rs10152101	14	45420311	CHRM3	1	239549875	240074741	SNIG	Not reported	9.12E−01	Not reported
rs11157423	14	45430189	CHRM3	1	239549875	240074741	SNIG	Not reported	9.22E−01	Not reported
rs11845904	14	45404328	A2BP1	16	5274364	7889892	SNIG	Not reported	6.38E−01	Not reported
rs17115703	14	45416918	A2BP1	16	5274364	7889892	SNIG	Not reported	9.42E−01	Not reported
rs10152101	14	45420311	A2BP1	16	5274364	7889892	SNIG	Not reported	9.12E−01	Not reported
rs11157423	14	45430189	A2BP1	16	5274364	7889892	SNIG	Not reported	9.22E−01	Not reported
rs11845904	14	45404328	MPP3	17	41878177	41910537	SNIG	Not reported	6.38E−01	Not reported
rs17115703	14	45416918	MPP3	17	41878177	41910537	SNIG	Not reported	9.42E−01	Not reported
rs10152101	14	45420311	MPP3	17	41878177	41910537	SNIG	Not reported	9.12E−01	Not reported
rs11157423	14	45430189	MPP3	17	41878177	41910537	SNIG	Not reported	9.22E−01	Not reported
rs2285013	14	103429825	TUBB1	20	57594309	57601709	SNIG	Not reported	6.12E−01	Not reported
rs2896462	14	103434739	TUBB1	20	57594309	57601709	SNIG	Not reported	5.84E−01	Not reported
rs17589695	14	103444293	TUBB1	20	57594309	57601709	SNIG	Not reported	4.54E−01	Not reported
rs10140685	14	103455008	TUBB1	20	57594309	57601709	SNIG	Not reported	4.94E−01	Not reported
rs1955666	14	45334238	CABP7	22	30098878	30127818	SNIG	Not reported	6.29E−01	Not reported
rs7141533	14	45348235	CABP7	22	30098878	30127818	SNIG	Not reported	4.64E−01	Not reported
rs11845904	14	45404328	CABP7	22	30098878	30127818	SNIG	Not reported	6.38E−01	Not reported
rs17115703	14	45416918	CABP7	22	30098878	30127818	SNIG	Not reported	9.42E−01	Not reported
rs10152101	14	45420311	CABP7	22	30098878	30127818	SNIG	Not reported	9.12E−01	Not reported
rs11157423	14	45430189	CABP7	22	30098878	30127818	SNIG	Not reported	9.22E−01	Not reported
rs11157424	14	45430216	CABP7	22	30098878	30127818	SNIG	Not reported	9.07E−01	Not reported
rs3825629	14	45432870	CABP7	22	30098878	30127818	SNIG	Not reported	8.95E−01	Not reported
rs11846656	14	45447187	CABP7	22	30098878	30127818	SNIG	Not reported	9.60E−01	Not reported
rs8006685	14	45521227	CABP7	22	30098878	30127818	SNIG	Not reported	8.75E−01	Not reported
rs10131615	14	45521583	CABP7	22	30098878	30127818	SNIG	Not reported	8.78E−01	Not reported
rs1053667	14	45543038	CABP7	22	30098878	30127818	SNIG	Not reported	9.85E−01	Not reported
rs11847336	14	45578043	CABP7	22	30098878	30127818	SNIG	Not reported	6.79E−01	Not reported
rs3825625	14	45604404	CABP7	22	30098878	30127818	SNIG	Not reported	8.77E−01	Not reported
rs2033385	14	45605818	CABP7	22	30098878	30127818	SNIG	Not reported	8.73E−01	Not reported
rs17660734	15	53606803	NTNG1	1	107682639	108073484	PUTM	Not reported	3.95E−01	Not reported
rs17660734	15	53606803	KCNJ3	2	155482391	155714866	PUTM	Not reported	3.95E−01	Not reported
rs17660734	15	53606803	CCDC136	7	128421937	128462179	PUTM	Not reported	3.95E−01	Not reported
rs17660734	15	53606803	NCALD	8	102698778	103136538	PUTM	Not reported	3.95E−01	Not reported
rs17660734	15	53606803	GABBR2	9	101050393	101471526	PUTM	Not reported	3.95E−01	Not reported
rs12910816	15	98249133	ZNF630	X	47917587	47931059	PUTM	Not reported	5.41E−01	Not reported
rs1661054	15	98253818	ZNF630	X	47917587	47931059	PUTM	Not reported	6.63E−01	Not reported
rs965305	15	98271950	ZNF630	X	47917587	47931059	SNIG	Not reported	5.56E−01	Not reported
rs12907561	15	98296413	ZNF630	X	47917587	47931059	SNIG	Not reported	7.72E−01	Not reported
rs422965	16	79651293	SNRNP200	2	96940080	96971284	SNIG	Not reported	3.77E−01	Not reported
rs422965	16	79651293	KIF1A	2	241650608	241776532	SNIG	Not reported	3.77E−01	Not reported
rs2006660	16	57716824	SLMAP	3	57741868	57958786	SNIG	Not reported	6.57E−01	Not reported
rs422965	16	79651293	DDX56	7	44604979	44613837	SNIG	Not reported	3.77E−01	Not reported
rs422965	16	79651293	CACNA2D1	7	81407194	82107638	SNIG	Not reported	3.77E−01	Not reported
rs422965	16	79651293	ZFP41	8	144329006	144343230	SNIG	Not reported	3.77E−01	Not reported
rs422965	16	79651293	PTER	10	16437524	16556561	SNIG	Not reported	3.77E−01	Not reported
rs422965	16	79651293	INCENP	11	61891470	61921181	SNIG	Not reported	3.77E−01	Not reported
rs422965	16	79651293	CLPB	11	71962014	72145689	SNIG	Not reported	3.77E−01	Not reported
rs422965	16	79651293	TSC2	16	2097410	2139784	SNIG	Not reported	3.77E−01	Not reported
rs422965	16	79651293	MED15	22	20847344	20941902	SNIG	Not reported	3.77E−01	Not reported
rs422965	16	79651293	SLC25A1	22	19163095	19166857	SNIG	Not reported	3.77E−01	Not reported
rs8064780	17	77753296	ARX	X	25019649	25035794	PUTM	Not reported	2.89E−01	Not reported
rs8081370	17	1373612	RPP40	6	4964408	5004270	SNIG	Not reported	9.15E−01	Not reported
rs11660459	18	46140691	RNF115	1	145611010	145706917	SNIG	Not reported	3.42E−01	Not reported
rs11664663	18	46143106	RNF115	1	145611010	145706917	SNIG	Not reported	2.51E−01	Not reported
rs11660459	18	46140691	MTMR14	3	9691156	9744176	SNIG	Not reported	3.42E−01	Not reported
rs11664663	18	46143106	MTMR14	3	9691156	9744176	SNIG	Not reported	2.51E−01	Not reported
rs162003	18	24448329	LEO1	15	52230222	52263977	SNIG	Not reported	4.99E−03	Not reported
rs162003	18	24448329	RNF167	17	4843323	4849241	SNIG	Not reported	4.99E−03	Not reported
rs2419720	19	13622333	ZNF630	X	47917587	47931059	PUTM	Not reported	3.97E−01	Not reported
rs12624956	20	37517753	MUT	6	49398079	49431547	PUTM	Not reported	1.37E−01	Not reported
rs1569539	20	37576160	BTBD1	15	83685185	83748655	PUTM	Not reported	6.29E−01	Not reported
rs12624956	20	37517753	UQCRC2	16	21944013	21994953	PUTM	Not reported	1.37E−01	Not reported
rs4813433	20	21565003	LOC284578	1	205525508	205538404	SNIG	Not reported	6.33E−01	Not reported
rs6132453	20	21642394	LOC284578	1	205525508	205538404	SNIG	Not reported	7.69E−01	Not reported
rs7281890	21	36565435	ALDH1A3	15	101420038	101456824	SNIG	Not reported	8.53E−01	Not reported
rs2834829	21	36566894	ALDH1A3	15	101420038	101456824	SNIG	Not reported	6.81E−01	Not reported
rs2013581	22	45449273	C19orf50	19	18668595	18672360	SNIG	Not reported	2.67E−01	Not reported
rs12853674	X	106134938	NBPF15	1	146032553	147400375	PUTM	Not reported	NA	Not reported
rs17244240	X	144194774	NBPF15	1	146032553	147400375	PUTM	Not reported	NA	Not reported
rs1975588	X	4961413	SIX3	2	45161424	45173196	PUTM	Not reported	NA	Not reported
rs1975588	X	4961413	HTR4	5	147830605	148045296	PUTM	Not reported	NA	Not reported
rs1975588	X	4961413	LHX6	9	124964884	124991104	PUTM	Not reported	NA	Not reported
rs1975588	X	4961413	GABRG3	15	27216527	27778363	PUTM	Not reported	NA	Not reported
rs2185026	X	138835240	TUBB1	20	57594309	57601709	PUTM	Not reported	NA	Not reported
rs45465799	X	138880835	TUBB1	20	57594309	57601709	PUTM	Not reported	NA	Not reported
rs1975588	X	4961413	ARX	X	25019649	25035794	PUTM	Not reported	NA	Not reported
rs150360221	X	154939085	XIST	X	72732456	73097069	PUTM	Not reported	NA	Not reported
rs150360221	X	154939085	XIST	X	73040495	73044457	PUTM	Not reported	NA	Not reported
rs150360221	X	154939085	XIST	X	73046573	73047763	PUTM	Not reported	NA	Not reported
rs150360221	X	154939085	XIST	X	73048903	73049066	PUTM	Not reported	NA	Not reported
rs150360221	X	154939085	XIST	X	73061354	73064395	PUTM	Not reported	NA	Not reported
rs5953544	X	140038594	WDR63	1	85464840	85599626	SNIG	Not reported	NA	Not reported
rs3946125	X	33315969	ERI3	1	44686799	44844914	SNIG	Not reported	NA	Not reported
rs973212	X	30178658	NLRC4	2	32449172	32500533	SNIG	Not reported	NA	Not reported
rs3946125	X	33315969	CLEC3B	3	45067659	45113188	SNIG	Not reported	NA	Not reported
rs3946125	X	33315969	HMGXB3	5	149379838	149432690	SNIG	Not reported	NA	Not reported
rs3946125	X	33315969	HIST1H1E	6	26156559	26157343	SNIG	Not reported	NA	Not reported
rs3946125	X	33315969	RXRB	6	33161368	33168744	SNIG	Not reported	NA	Not reported
rs3946125	X	33315969	FSCN1	7	5632449	5666041	SNIG	Not reported	NA	Not reported
rs3946125	X	33315969	GIGYF1	7	100276189	100287061	SNIG	Not reported	NA	Not reported
rs3946125	X	33315969	CREB3	9	35728110	35738884	SNIG	Not reported	NA	Not reported
rs3946125	X	33315969	RAB1B	11	66036024	66044937	SNIG	Not reported	NA	Not reported
rs3946125	X	33315969	ARFGAP2	11	47185875	47198538	SNIG	Not reported	NA	Not reported
rs3946125	X	33315969	THTPA	14	23980989	24030089	SNIG	Not reported	NA	Not reported
rs3946125	X	33315969	MAP2K5	15	67835021	68104859	SNIG	Not reported	NA	Not reported
rs3946125	X	33315969	LOC342346	16	4606491	4654823	SNIG	Not reported	NA	Not reported
rs3946125	X	33315969	SPNS1	16	28985662	28995858	SNIG	Not reported	NA	Not reported
rs3946125	X	33315969	CD2BP2	16	30362096	30366662	SNIG	Not reported	NA	Not reported
rs3946125	X	33315969	RNF167	17	4843323	4849241	SNIG	Not reported	NA	Not reported
rs3946125	X	33315969	UBE2Z	17	46985809	47006871	SNIG	Not reported	NA	Not reported
rs3946125	X	33315969	SLC25A19	17	73269074	73285514	SNIG	Not reported	NA	Not reported
rs3946125	X	33315969	EVI5L	19	7895104	7929855	SNIG	Not reported	NA	Not reported
rs3946125	X	33315969	MAP2K7	19	7968563	7983972	SNIG	Not reported	NA	Not reported
rs3946125	X	33315969	UBL5	19	9932207	9940787	SNIG	Not reported	NA	Not reported
rs3946125	X	33315969	NACC1	19	13229091	13251956	SNIG	Not reported	NA	Not reported
rs3946125	X	33315969	PIK3R2	19	18263646	18281333	SNIG	Not reported	NA	Not reported
rs3946125	X	33315969	TBC1D17	19	50380643	50391984	SNIG	Not reported	NA	Not reported
rs5924103	X	86998758	ZNF578	19	53005080	53017275	SNIG	Not reported	NA	Not reported
rs3946125	X	33315969	ELOF1	19	11661972	11670131	SNIG	Not reported	NA	Not reported
rs3946125	X	33315969	PPP1R14A	19	38735481	38747172	SNIG	Not reported	NA	Not reported
rs3946125	X	33315969	SIRT2	19	39369211	39390361	SNIG	Not reported	NA	Not reported
rs3946125	X	33315969	DMWD	19	46287501	46296075	SNIG	Not reported	NA	Not reported
rs3946125	X	33315969	RPL18	19	49118629	49122773	SNIG	Not reported	NA	Not reported
rs3946125	X	33315969	ASPDH	19	51014874	51017937	SNIG	Not reported	NA	Not reported
rs2185026	X	138835240	TUBB1	20	57594309	57601709	SNIG	Not reported	NA	Not reported
rs45465799	X	138880835	TUBB1	20	57594309	57601709	SNIG	Not reported	NA	Not reported
rs6603475	X	118213990	EDN3	20	57865163	57941017	SNIG	Not reported	NA	Not reported
rs3747383	X	118214081	EDN3	20	57865163	57941017	SNIG	Not reported	NA	Not reported
rs12393459	X	118215732	EDN3	20	57865163	57941017	SNIG	Not reported	NA	Not reported
rs3946125	X	33315969	TMEM184B	22	38615298	38669199	SNIG	Not reported	NA	Not reported
rs3946125	X	33315969	CBX6	22	39254538	39268284	SNIG	Not reported	NA	Not reported
rs12010103	X	39254743	ZNF630	X	47917587	47931059	SNIG	Not reported	NA	Not reported
rs150360221	X	154939085	XIST	X	72732456	73097069	SNIG	Not reported	NA	Not reported
rs150360221	X	154939085	XIST	X	73040495	73044457	SNIG	Not reported	NA	Not reported
rs150360221	X	154939085	XIST	X	73046573	73047763	SNIG	Not reported	NA	Not reported
rs150360221	X	154939085	XIST	X	73048903	73049066	SNIG	Not reported	NA	Not reported
rs150360221	X	154939085	XIST	X	73061354	73064395	SNIG	Not reported	NA	Not reported

The *trans*-eQTLs (519 variants) were merged through SNPs with the following datasets/databases:^1^ GWAS_Cat_AD: The GWAS Catalog with search parameters ‘Alzheimer’s Disease’;^2^ *p* value_Nalls: Parkinson’s Disease Meta-Analysis of GWAS by Nalls et al. (2019);^3^ GWAS_Cat_PD: The GWAS Catalog with search parameters ‘Parkinsons’s Disease’. SNPs/eQTLs combined with GWAS databases/datasets [i.e.^1,3^] are indicated either ‘Reported/Not reported’^2^. Nalls et al. *P-*values are available. Cells indicated by *NA* were not in the databases/datasets.
