# Supplementary material for: Genome-wide human brain eQTLs: In-depth analysis and insights using the UKBEC dataset
Source: Sci Rep. 2019 Dec 16;9:19201. doi: 10.1038/s41598-019-55590-0 (PMC6915738; doi:10.1038/s41598-019-55590-0)
Supplement: Supplementary file 1 — Supplementary Material [file 41598_2019_55590_MOESM1_ESM.pdf]

Genome-wide human brain eQTLs: In-depth analysis and insights using the UKBEC dataset

Supplementary Material

Letitia M. F. Sng[1], Peter C. Thomson[1] and Daniah Trabzuni[2,3]

[1]The University of Sydney, School of Life and Environmental Sciences, New South Wales 2006, Australia

[2] Department of Neurodegenerative Diseases, UCL Queen Square Institute of Neurology, Queen Square, London WC1N 3BG, UK

[3] Department of Genetics, King Faisal Specialist Hospital and Research Centre, 11211 Riyadh, Saudi Arabia

S1 Selection of SNPs for eQTL Mapping

A total of 6,455,152 genotyped and imputed SNPs were recorded but only genotyped SNPs were used in the analysis (n = 788,474). A filter of the minor allele frequency (MAF) > 5% was then applied, reducing the number of SNPs to 787,220 (i.e. 99.8% of SNPs had MAF >5%). Next, any SNP that was missing in any of the 134 samples was omitted, reducing the number of available SNPs to 720,851 (i.e. 91.6% of SNPs had a complete set of genotypes). One advantage of these filtering steps is that each SNP has equal power for detection of eQTLs *a priori* (i.e. no bias is introduced by some SNPs having fewer replicates, with consequent loss of power). Another advantage is that while the imputed SNP data could have been used, the high SNP density implies a close distance between SNPs, and many will be in extremely high linkage disequilibrium (LD). Given that SNP imputation involves using information from SNPs that are in tight LD, effectively no additional information can be provided from these imputed SNPs, in terms of power to detect associations that cannot

already be inferred from the observed SNPs. There is also considerable computational benefit in working with a smaller data set, and hence not making as many tests of associations.

S2 Simulating Linkage Disequilibrium between SNPs

When simulating genotype data, 20 chromosomes of equal length (L = 1) were simulated with 1,000 SNPs per chromosome ($n_{\text{SNP.pc}}$ = 1,000). A vector **p** of length $n_{\text{SNP.pc}}$ of allele frequencies was drawn from a beta distribution, Beta($a - \delta$, $b - \delta$). Parameters a and b were chosen in the same fashion as the linkage equilibrium scenario while the correction $\delta = 0.05$ was applied to ensure similar overall allele frequency distribution to that in the LE scenario, resulting from the non-independence of SNPs in the LE scenario. A set of SNP positions, $\mathbf{d}_{\text{SNP}}$, was generated from uniform distributions, U(0, L) and sorted in ascending order. For $n_{\text{SNP.pc}}$ positions, a $n_{\text{SNP.pc}} \times n_{\text{SNP.pc}}$ correlation matrix was calculated based on an exponential correlation model of distance between SNPs:

$$\mathbf{R} = \exp(-k\mathbf{D})$$

where $k = 5L$, the exponential correlation decay parameter and **D** is a matrix of absolute distances between all SNPs on the chromosome, $\mathbf{D} = \{D_{ij}$ where $D_{ij} = |d_{\text{SNP},i} - d_{\text{SNP},j}|$, $i = 1, \ldots, n_{\text{SNP.pc}}$; , $j = 1, \ldots, n_{\text{SNP.pc}}$.

The variance-covariance matrix between SNP positions was then calculated, on the underlying scale:

$$\mathbf{V} = \sigma_\varepsilon^2 \mathbf{I}_{n_{\text{SNP.pc}}} + \sigma_u^2 \mathbf{R}$$

where σ_ε^2 (set as 1) is the residual variance on the underlying scale, and σ_u^2 (set as 2) is the variance for the spatial correlation process.

A total of n_{sample} random vectors **z** was then generated from a multivariate normal distribution N(0, **V**), and then rescaled by multiplying $\pi/\sqrt{3}$ so as to correspond to observations from a logistic distribution.

n_{sample} vectors logit($\mathbf{p}$) were calculated where logit(p_i) = $\log_e[p_i/(1-p_i)]$, $i = 1, \ldots, n_{\text{SNP.pc}}$. The sequence of correlated values along the length of the chromosome was calculated for the n_{sample} vectors with logit($\mathbf{p}_{\text{c}}$) ← logit($\mathbf{p}$) + $\mathbf{z}$ with separate series for each of the n_{sample} observations. A sequence of cumulative probabilities, $\mathbf{p}_{\text{c}} = 1 / [1 + \exp(-\text{logit}(\mathbf{p}_{\text{c}})]$ was back-transformed, again with one sequence for each of the n_{sample} observations.

Lastly, the following thresholds were applied to form the genotypes (0, 1 or 2 copies of the SNP allele);

$$x_i = \begin{cases} 0 & p_{\text{c},i} \le 0.25 \\ 1 & 0.25 < p_{\text{c},i} \le 0.75 \\ 2 & p_{\text{c},i} > 0.75 \end{cases}, \; i = 1, \ldots, n_{\text{SNP.pc}}$$

The genotypes were then compiled into a matrix $\mathbf{X}$ with dimensions $n_{\text{SNP.pc}} \times n_{\text{sample}}$.

An overall similar distribution of allele frequencies to that produced in the LE simulations but with correlated SNPs was also produced. These allele frequencies along with matrix $\mathbf{X}$ was used to generate expression phenotypes as with the LE simulations and so forth.

S3 Genotyping Error Simulation Parameters

As mentioned in the main text, genotyping error parameters were calculated from the cross-classification of SNPs from microarrays and sequencing reported by Rogers, et al. [1] where the sequencing data were assumed to be more accurate and thus considered the “true genotype”. Supplementary Table 1 shows these parameters with each cell reporting the probability of returning an “observed genotype” column given the “true genotype” row. Consequently, each row sums to unity.

Supplementary Table 1. Genotyping error parameters with each cell reporting the probability of returning an "observed genotype" column given the "true genotype" row. Consequently, each row sums to unity.

True Genotype	Observed Genotype		
	**AA**	**AB**	**BB**
**AA**	0.99949226	0.000496583	0.000011553
**AB**	0.00153717	0.998016932	0.000445896
**BB**	0.00016294	0.008091098	0.991745965

The appropriate row of the table, given the "true" SNP genotypes simulated, was used as event probabilities. For example, given that the true genotype is AA, there is a probability of 0.000496583 that the error genotype will be returned as AB. The resulting probability vector was used to generate SNPs with genotype error from multinomial distributions and used as the genotyping data for MatrixEQTL [2], as opposed to the "true" genotype matrix.

S4 Designation of *cis*- vs *trans*-eQTLs

The following plots (S4.1, S4.2, S4.3) show distribution of distances between the transcript and the SNP, for eQTLs where the transcript and the SNP are located on the same chromosome (shown on a logarithm base 10 scale). It shows a clear separation in frequency at distances between 10^6 and 10^7 base pairs. Importantly, this same separation is seen across all brain regions and all chromosomes, suggesting that using a common cut-point is appropriate. Thus, a cut-point of $10^{6.5}$ = 3.16 Mb was used to classify eQTLs *cis* versus *trans*.

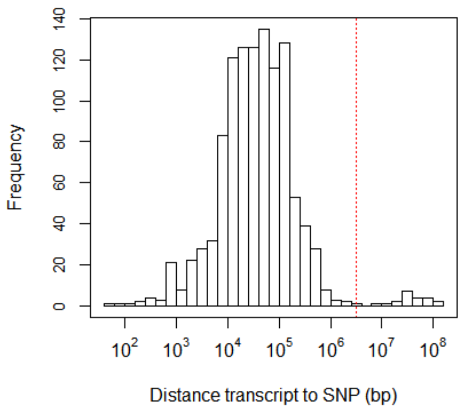

Figure S4.1 A histogram of the distance of transcript to SNP (when on the same chromosome). A bell-shaped distribution can be observed when transcripts are closer to the SNP but there is another smaller bell-shaped curve pass the cut-point of $10^{6.5}$ (i.e. the dotted red-line).

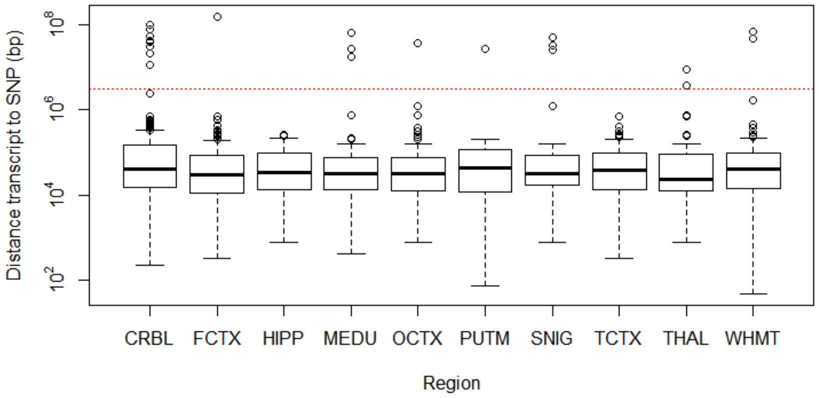

Figure S4.2 Box plots of the distance of transcripts to SNP (in base pairs) for each of the ten regions. For all regions, the cut-point of $10^{6.5}$ bp dissects the frequency of eQTLs clearly with none of the regions having a different distribution that requires a different cut-point.

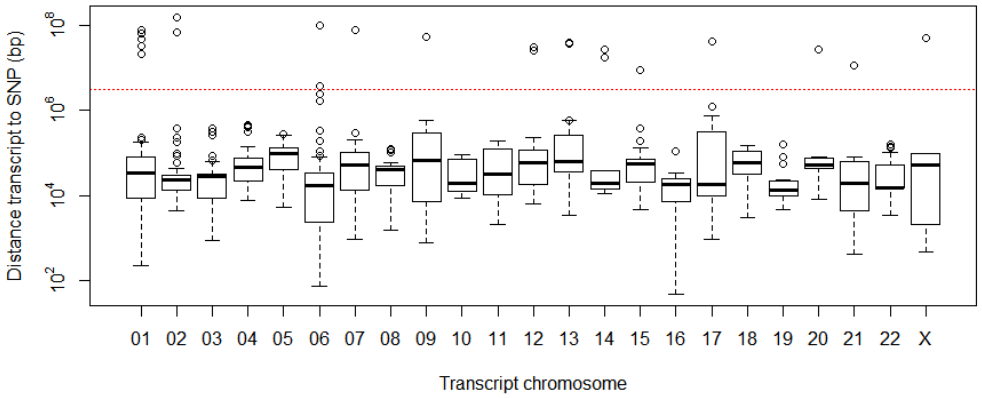

Figure S4.3 Box plots of the distance of transcript to SNP in base-pairs for each transcript chromosome. Similar to Figure S4.2, the cut-point of $10^{6.5}$ bp separates the distributions of eQTLs cleanly for all chromosomes.

S5 Comparison with previous eQTL analysis

The data analysed and presented in this paper are the UKBEC dataset. Previous analyses of the same data set were reported by Ramasamy, et al. [3]. As somewhat different approaches have been used (e.g. SNP selection, definition of *cis*- vs *trans*-acting eQTLs), the number of transcript-level eQTLs identified in common, or by the other data set only, have been tabulated, separately for each brain region (Supplementary Table 2). What is considered to be 'the same' eQTL has been evaluated in terms of SNP position within 0.25 Mb, 0.5 Mb, and 1 Mb. In addition, some different aspects and eQTL mapping procedures were applied here. As shown in Supplementary Table 2, very similar numbers of eQTLs were mapped in the original study [3] and the present study, even when considered by region. There are relatively few changes when the definition of the 'same eQTLs' is changed from within 0.25 Mb to within 2 Mb, but as expected, more are flagged as the "same eQTLs" as the interval is increased. Of the eQTLs mapped in the present study, the majority (over 90% in some regions) were usually found in the original study [3] study. However, it is apparent that a much

smaller proportion of eQTLs mapped by the original study [3] were detected in the current study. Possibly, this difference relates to the initial filtering of SNPs undertaken prior to eQTL mapping, and the selection of the SNP representing an eQTL (i.e. the most significant SNP association within an LD block).

Supplementary Table 2. Comparison of transcript-level eQTLs mapped by Ramasamy *et al.* (2014) (R) and the present study (S).

	# eQTLs mapped		% eQTLs in R mapped in S			% eQTLs in S mapped in R		
	R	S	< 0.25 Mb	< 0.5Mb	< 1 Mb	< 0.25 Mb	< 0.5Mb	< 1 Mb
CRBL	2142	1956	43.5	44.5	45.1	90.2	90.4	90.4
FCTX	906	887	44.0	45.8	45.9	97.5	97.5	97.5
HIPP	760	824	21.4	41.4	41.6	46.2	94.1	94.9
MEDU	628	597	19.6	39.6	40.0	29.0	95.6	95.6
OCTX	712	947	29.6	47.9	47.9	55.1	97.1	97.1
PUTM	382	404	24.9	40.3	40.3	41.3	87.1	87.1
SNIG	430	453	41.6	41.6	42.8	92.5	92.7	93.8
TCTX	1137	956	40.3	41.2	41.2	94.7	94.9	94.9
THAL	614	759	39.7	40.4	40.4	92.9	93.1	93.1
WHMT	1173	1071	45.9	46.5	47.2	93.7	93.7	94.0
Overall	8884	8854	37.6	43.6	43.9	77.7	93.6	93.8

The table compares the number of eQTLs mapped by the study by Ramasamy *et al.* (2014) (R) and the present study (S). Note that the eQTLs used for comparison in this table were the total number of eQTLs found at the transcript-level. It is also showing the percentage mapped by S of the eQTLs mapped by R, and the percentage mapped by R of the eQTLs mapped by S. For the two inclusion percentages, these are shown within varying distances between SNP locations in the two studies. For example, of the 2,142 eQTLs mapped in the cerebellum (CRBL) by R, 43.5% were mapped in S with SNP locations within < 0.25 Mb. As the distance between the SNP locations (i.e. SNP location in R versus SNP location in S) increases, the percentage of eQTLs mapped also increases (whether it is R in S or vice versa).

S6 Simulation to evaluate FDR for *cis*-acting vs *trans*-acting eQTLs

A single LE simulation was run as described in the materials and methods with MatrixEQTL[2] output from all possible associations kept (i.e. all 400,000,000 possible SNP-transcript combinations). A *cis*-acting eQTL was defined as a SNP at the same location as a transcript

while a *trans*-acting eQTL was everything else (i.e. 20,000 potential *cis*-acting eQTLs, 399,980,000 potential *trans*-acting eQTLs). A new FDR was calculated based on the *P*-values of *cis*-acting and *trans*-acting eQTLs separately.

Figure S6.1 shows that by using a pooled FDR for both *cis*-acting and *trans*-acting eQTLs, we are not inflating the number of significant *trans*-acting eQTLs (FDR ≤ 0.01). This is further supported by Figure S6.2. Furthermore, we plotted two separate smoothed scatter plots of estimated effect sizes against true effect sizes for *cis*-acting and *trans*-acting eQTLs (Figure S6.3). As shown, when eQTLs are present, the estimated effect sizes are close to the true effect sizes. Although, this is clearer for *cis*-acting eQTLs, the estimated effect sizes of *trans*-acting eQTLs are still within acceptable limits.

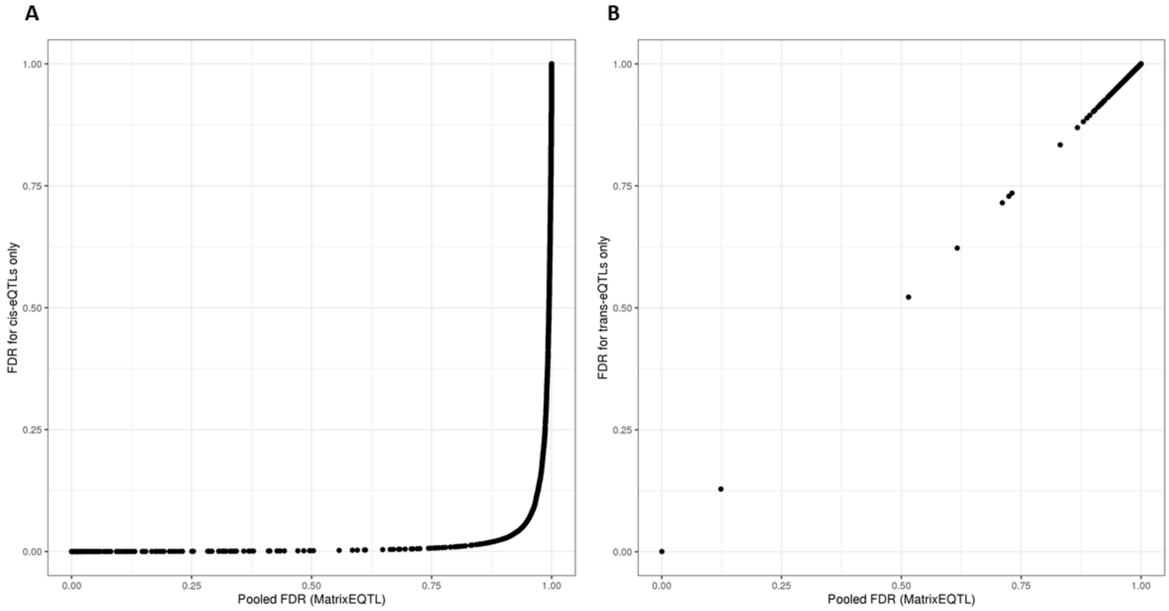

Figure S6.1 Scatterplots of pooled FDR (based on distributions of *P*-values of both *cis*-acting and *trans*-acting eQTLs) against separate FDRs calculated for (A) *cis*-acting and (B) *trans*-acting eQTLs. Due to the large number of potential *trans*-acting eQTLs, a random sample of 20,000 was used to plot the FDR specific to *trans*-acting eQTLs.

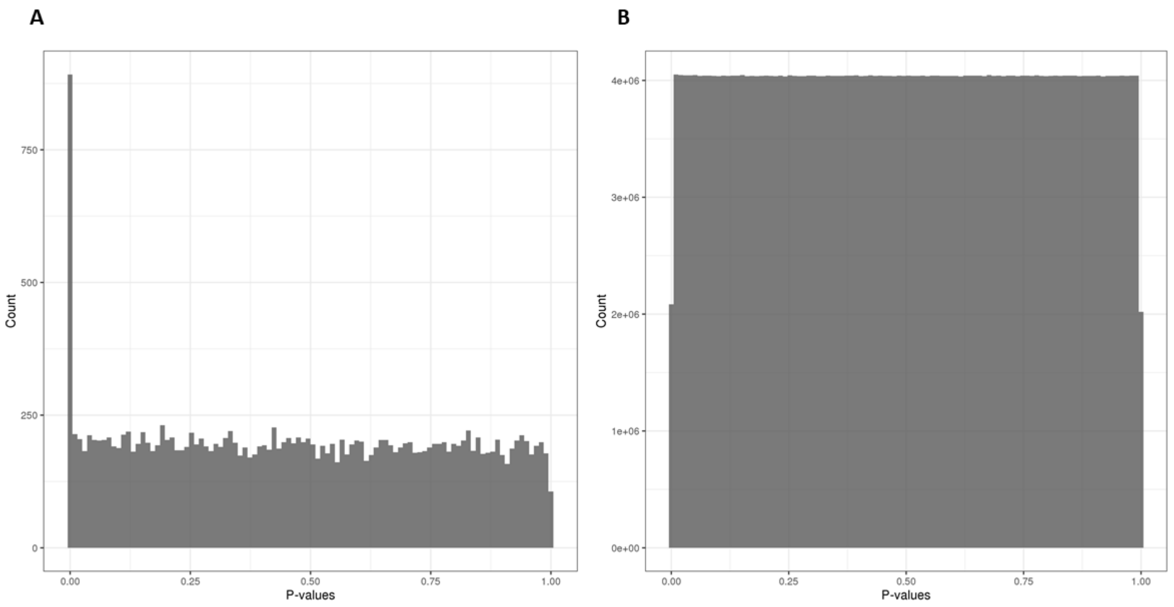

Figure S6.2 Histograms of *P*-values for (A) *cis*-acting and (B) *trans*-acting eQTLs. Together, these represent *P*-values for all possible 400,000,000 SNP-transcript combinations simulated.

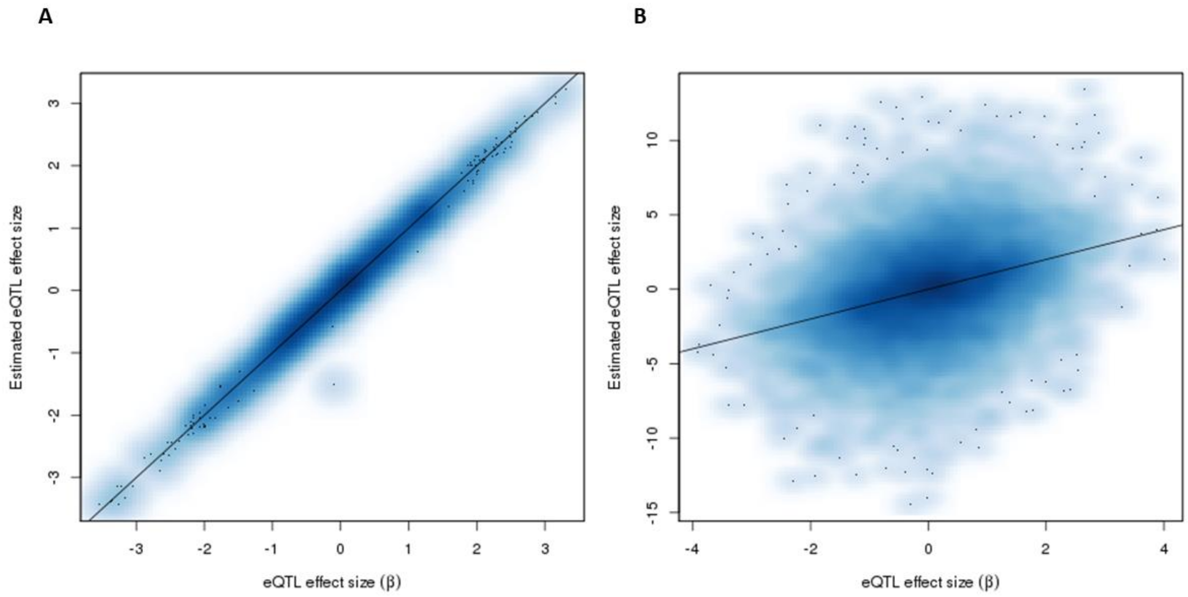

Figure S6.3 Separate smoothed scatterplots of the estimated effect sizes from MatrixEQTL against the true simulated effect sizes for (A) *cis*-acting and (B) *trans*-acting eQTLs. It is clear that when an eQTL is present, the estimated effect size (*y*-axis) is close to the true effect size (*x*-axis). This is especially true for *cis*-acting eQTLs.

References

1 Rogers, A., Beck, A. & Tintle, N. L. Evaluating the concordance between sequencing, imputation and microarray genotype calls in the GAW18 data. *BMC Proc* **8**, S22, doi:10.1186/1753-6561-8-S1-S22 (2014).

2 Shabalin, A. A. Matrix eQTL: ultra fast eQTL analysis via large matrix operations. *Bioinformatics* **28**, 1353-1358, doi:10.1093/bioinformatics/bts163 (2012).

3 Ramasamy, A. *et al.* Genetic variability in the regulation of gene expression in ten regions of the human brain. *Nat Neurosci* **17**, 1418-1428, doi:10.1038/nn.3801 (2014).

Supplementary Figures

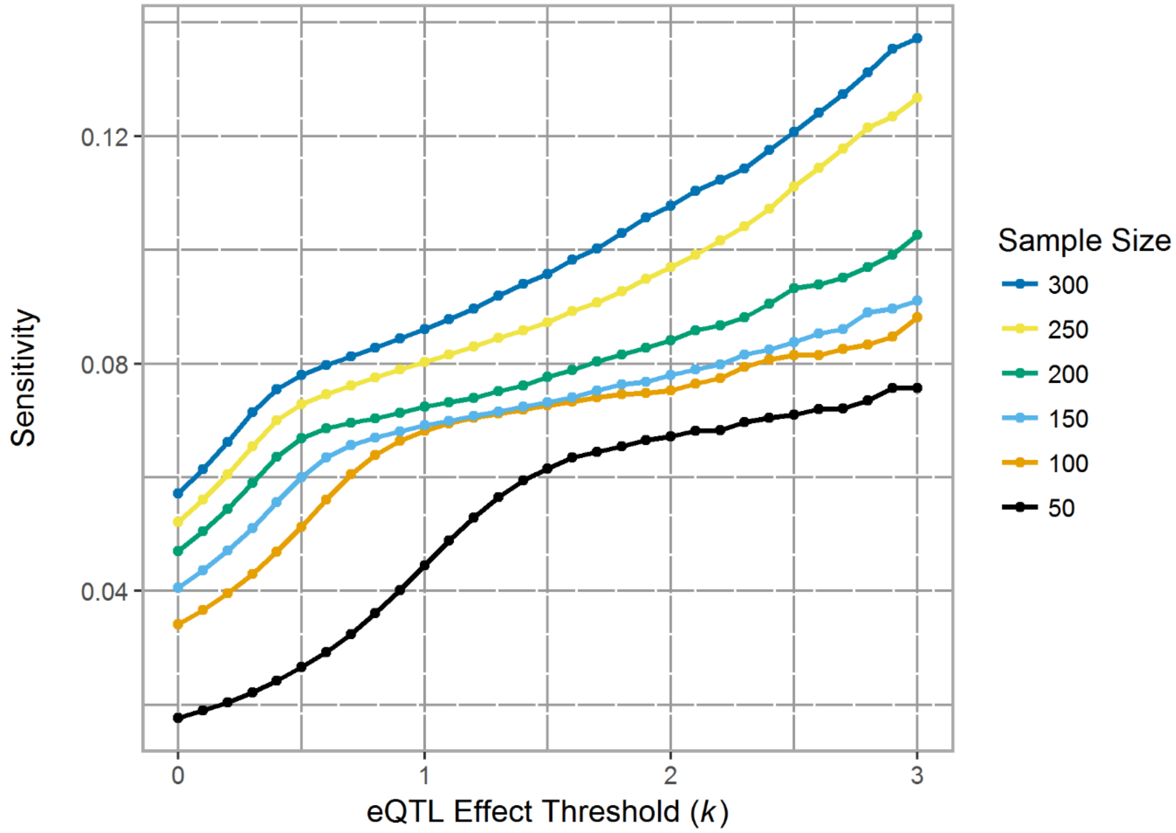

Figure S1. The sensitivity to detect eQTLs at a range of effect sizes thresholds for Scenario 2: Linkage Disequilibrium (LD). A plot that shows the average sensitivity from 100 simulations in each sample size (n = 50, 100, 150, 200, 250, 300) to detect a range of eQTL effect size thresholds, k (0 – 3 in 0.1 increments) when SNPs are in LD. As both sample size and effect size threshold increase, the sensitivity to detect eQTLs increases. However, sensitivity is very low across all sample sizes and effect size thresholds, with the greatest level of sensitivity (i.e. when $n = 300$, $k = 3$) being less than 0.14. At lower effect size thresholds $k \leq 1$, sensitivity to detect eQTLs across sample sizes (except $n = 50$) is comparable.

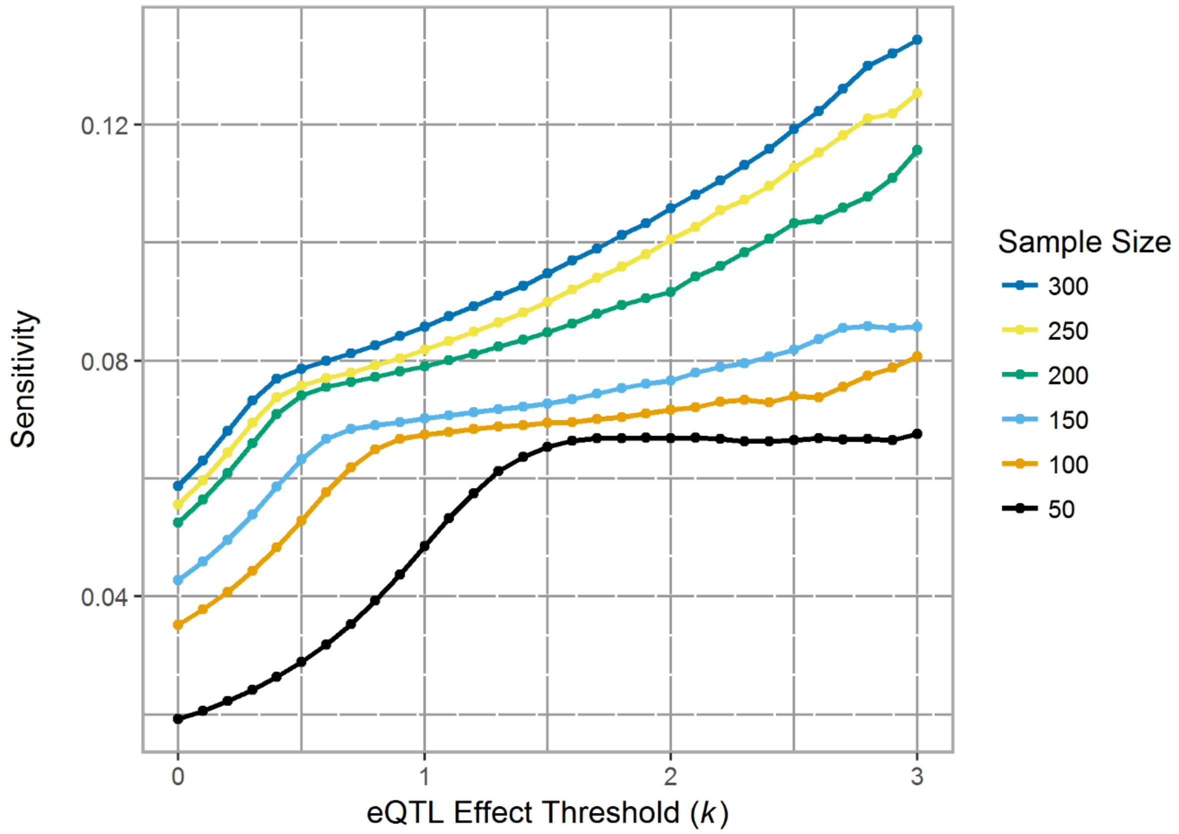

Figure S2. The sensitivity to detect eQTLs at a range of effect sizes thresholds for Scenario 3: Genotyping Error (GE). A plot that shows the average sensitivity from 100 simulations in each sample size (*n* = 50, 100, 150, 200, 250, 300) to detect a range of eQTL effect size thresholds, *k* (0 – 3 in 0.1 increments) when genotyping error bias is introduced. As both sample size and effect size threshold increase, the sensitivity to detect eQTLs increases. However, sensitivity is very low across all sample sizes and effect size thresholds, with the greatest level of sensitivity (i.e. when $n = 300$, $k = 3$) being less than 0.14. At lower effect size thresholds $k \leq 1$, sensitivity to detect eQTLs across sample sizes (except $n = 50$) is comparable, especially true when $n \geq 200$.

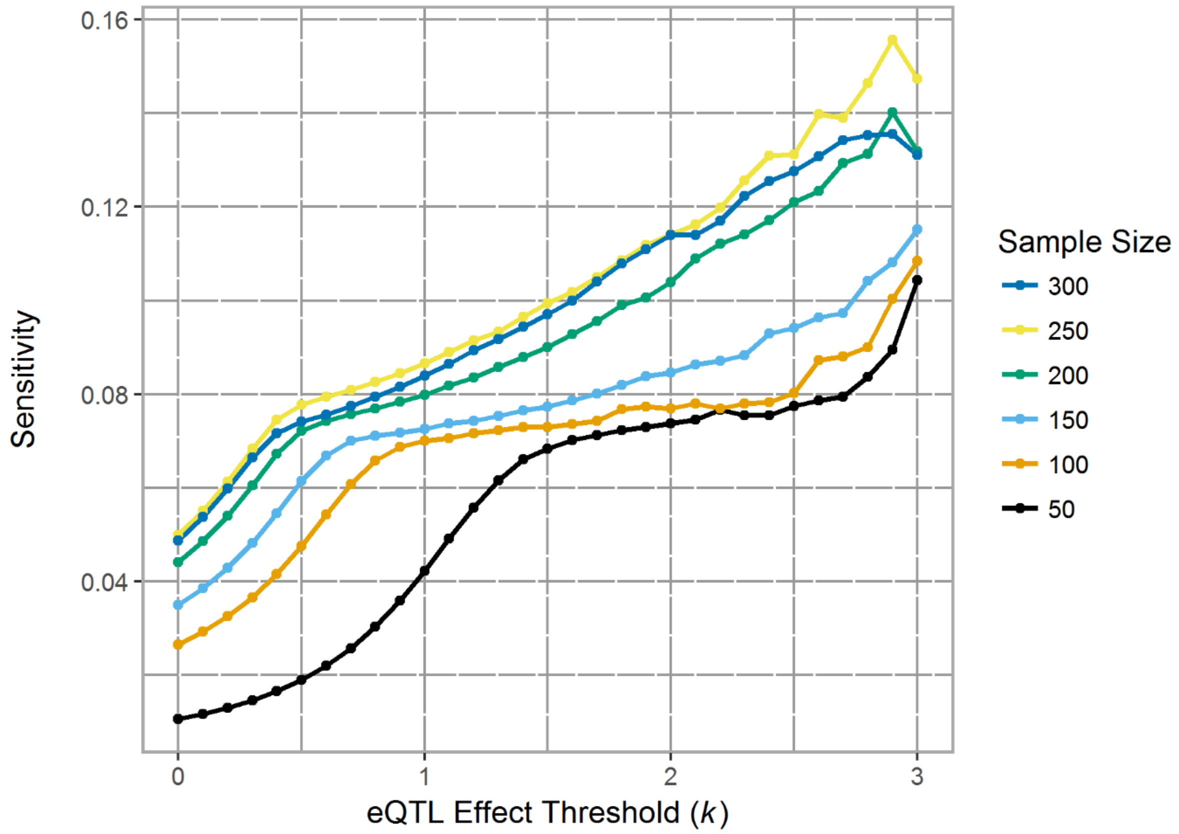

Figure S3. **The sensitivity to detect eQTLs at a range of effect size thresholds for Scenario 4: Lower expression level variance compared with residual variance (LV).** A plot that shows the average sensitivity from 100 simulations in each sample size (n = 50, 100, 150, 200, 250, 300) to detect a range of eQTL effect size thresholds, k (0 – 3 in 0.1 increments) when eQTL expression levels were simulated with lower variance (σ^2_β) than model residual error variance (σ^2_ε). As both sample size and effect size threshold increase, the sensitivity to detect eQTLs increases. However, sensitivity is very low across all sample sizes and effect size thresholds, with the greatest level of sensitivity (i.e. when n = 300, k = 2.9) being less than 0.16, though overall, sensitivity in this scenario is higher when compared to previous scenarios. At lower effect size thresholds $k \leq 1$, sensitivity to detect eQTLs across sample sizes (except n = 50) is comparable. At higher sample sizes ($n \geq 200$), there seems to be an unexpected decrease in sensitivity at higher effect thresholds ($k \geq 2.9$).

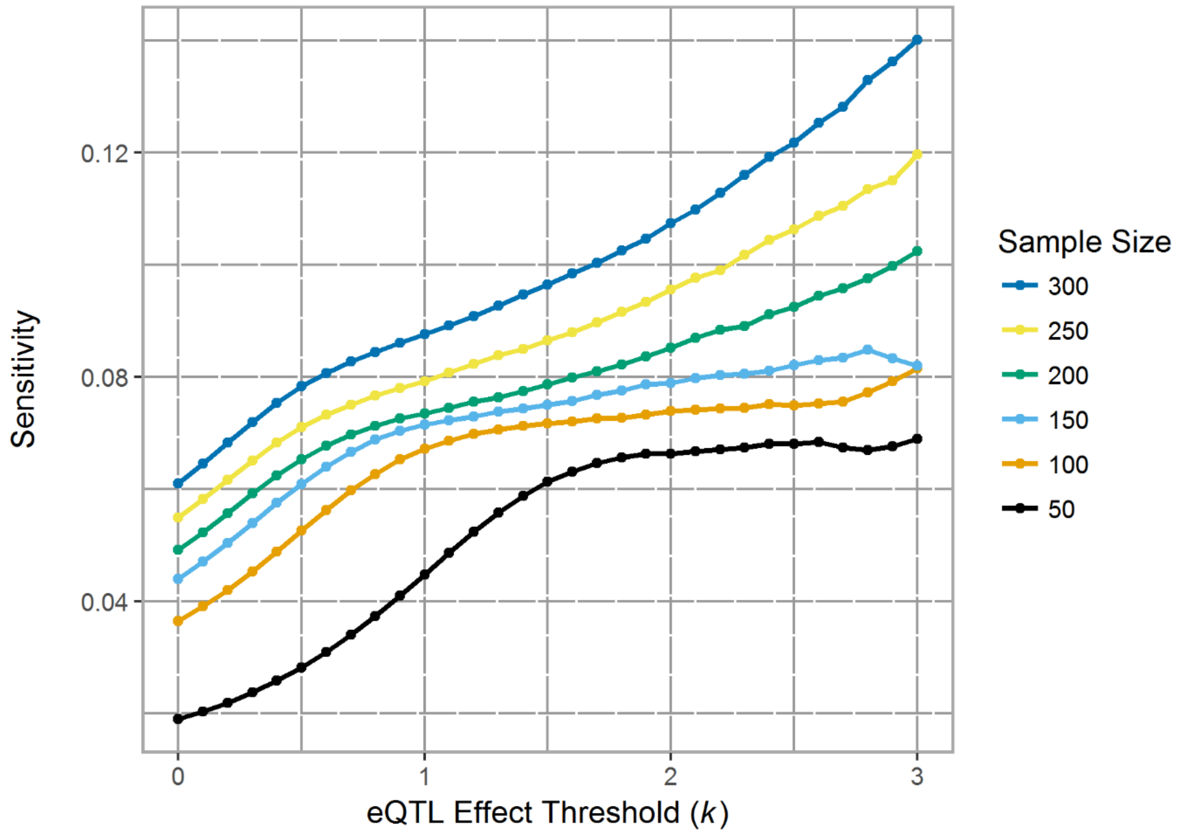

Figure S4. **The sensitivity to detect eQTLs at a range of effect size thresholds for Scenario 5: Dominance (Dom).** A plot that shows the average sensitivity from 100 simulations in each sample size (n = 50, 100, 150, 200, 250, 300) to detect a range of eQTL effect size thresholds, k (0 – 3 in 0.1 increments) when dominance effect bias is introduced. As both sample size and effect size threshold increase, the sensitivity to detect eQTLs increases. However, sensitivity is very low across all sample sizes and effect size thresholds, with the greatest level of sensitivity (i.e. when n = 300, k = 3) equal to 0.14. At lower effect size thresholds $k \leq 1$, sensitivity to detect eQTLs across sample sizes (except n = 50) is comparable. Sensitivity seem to decrease when $k \geq 2.8$ for n = 150.

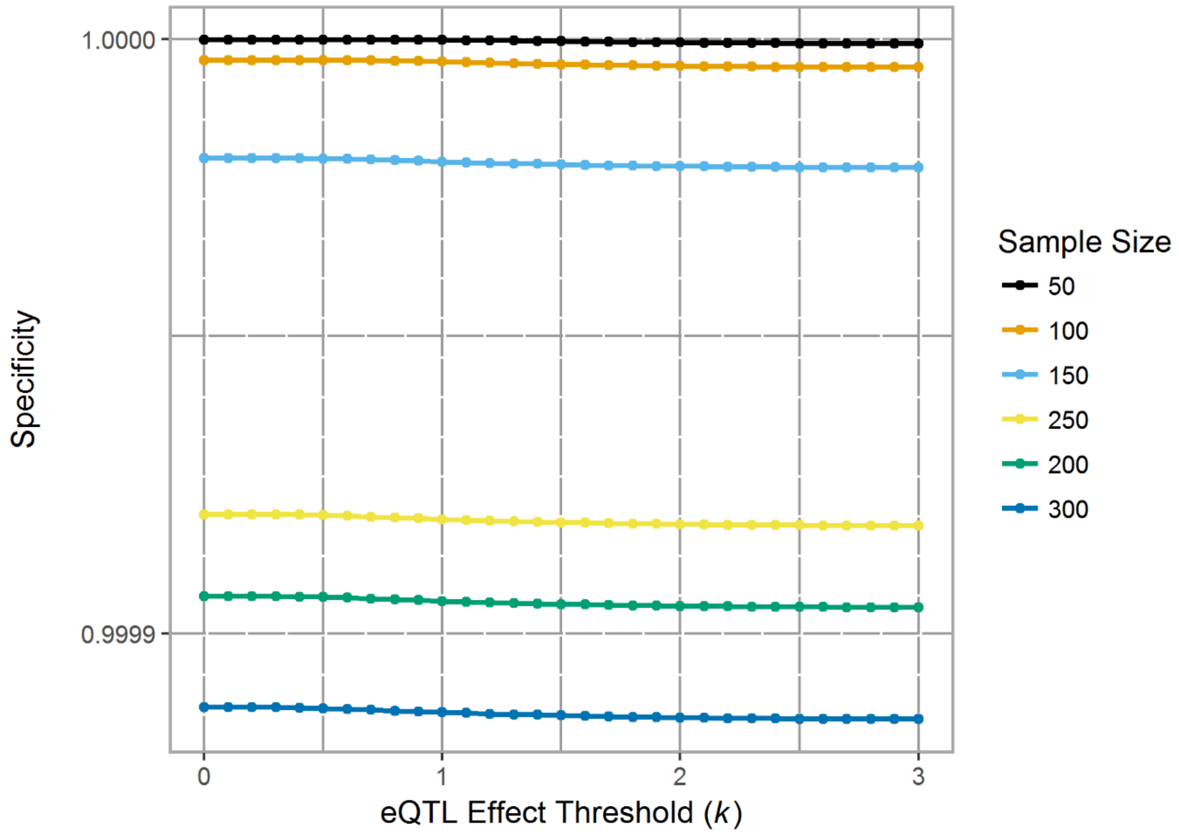

Figure S5. The specificity to detect eQTLs at a range of effect size thresholds for Scenario 2: Linkage Disequilibrium (LD). A plot that shows the average specificity from 100 simulations in each sample size (*n* = 50, 100, 150, 200, 250, 300) to detect a range of eQTL effect size thresholds, *k* (0 – 3 in 0.1 increments) when SNPs were in LD. As sample size increases, the specificity to detect eQTLs decreases though *n* = 250 is showing higher specificity compared to *n* = 200 and *n* = 300. However, specificity is very high across all sample sizes and effect size thresholds with the lowest level of specificity (i.e. when *n* = 300, *k* = 3) being greater than 0.99985. For each sample size, there is no discernible decrease in specificity as the effect size threshold increase, unlike the increase in sensitivity.

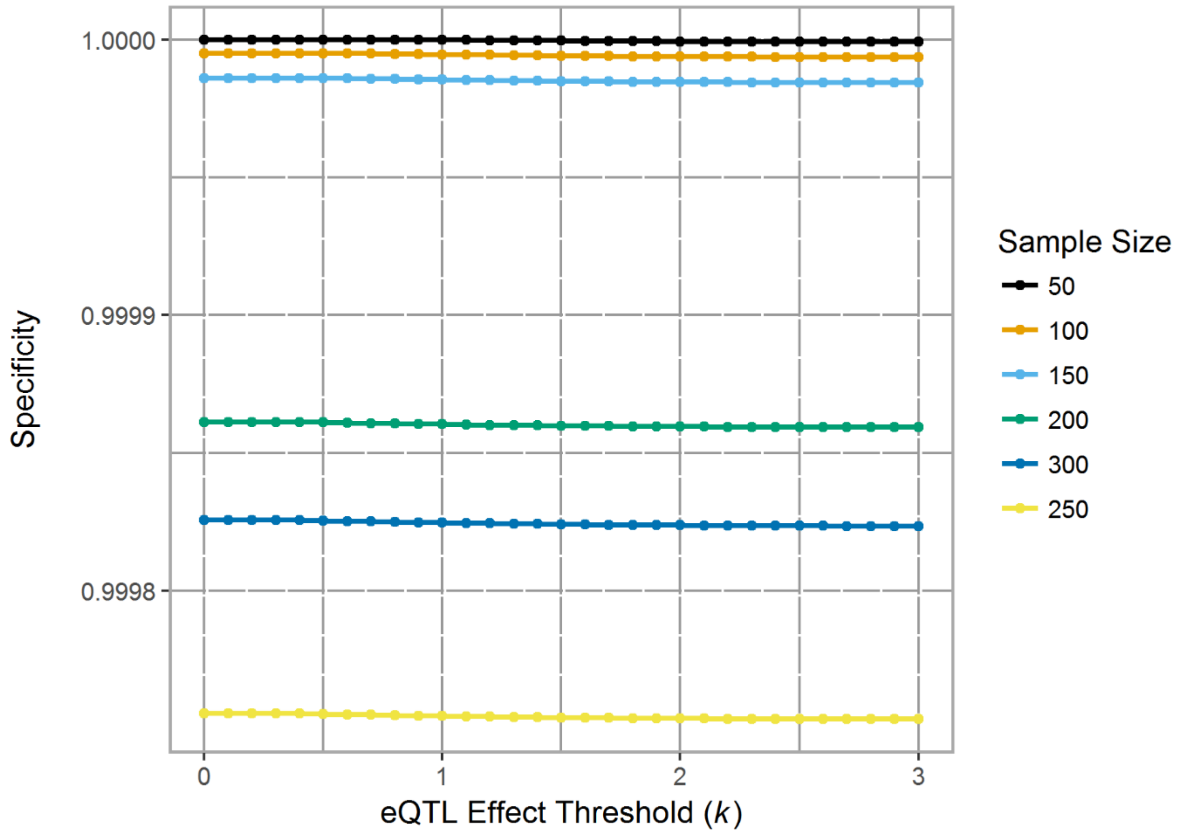

Figure S6. The specificity to detect eQTLs at a range of effect size thresholds for Scenario 3: Genotyping Error (GE). A plot that shows the average specificity from 100 simulations in each sample size (*n* = 50, 100, 150, 200, 250, 300) to detect a range of eQTL effect size thresholds, *k* (0 – 3 in 0.1 increments) when genotyping error bias is introduced. As sample size increases, the specificity to detect eQTLs decreases but note that *n* = 250 has lower specificity than *n* = 300 (i.e. this decrease is not monotonic). However, specificity is very high across all sample sizes and effect size thresholds with the lowest level of specificity (i.e. when *n* = 250, *k* = 3) being greater than 0.9997. For each sample size, there is no discernible decrease in specificity as the effect size threshold increase, unlike the increase in sensitivity.

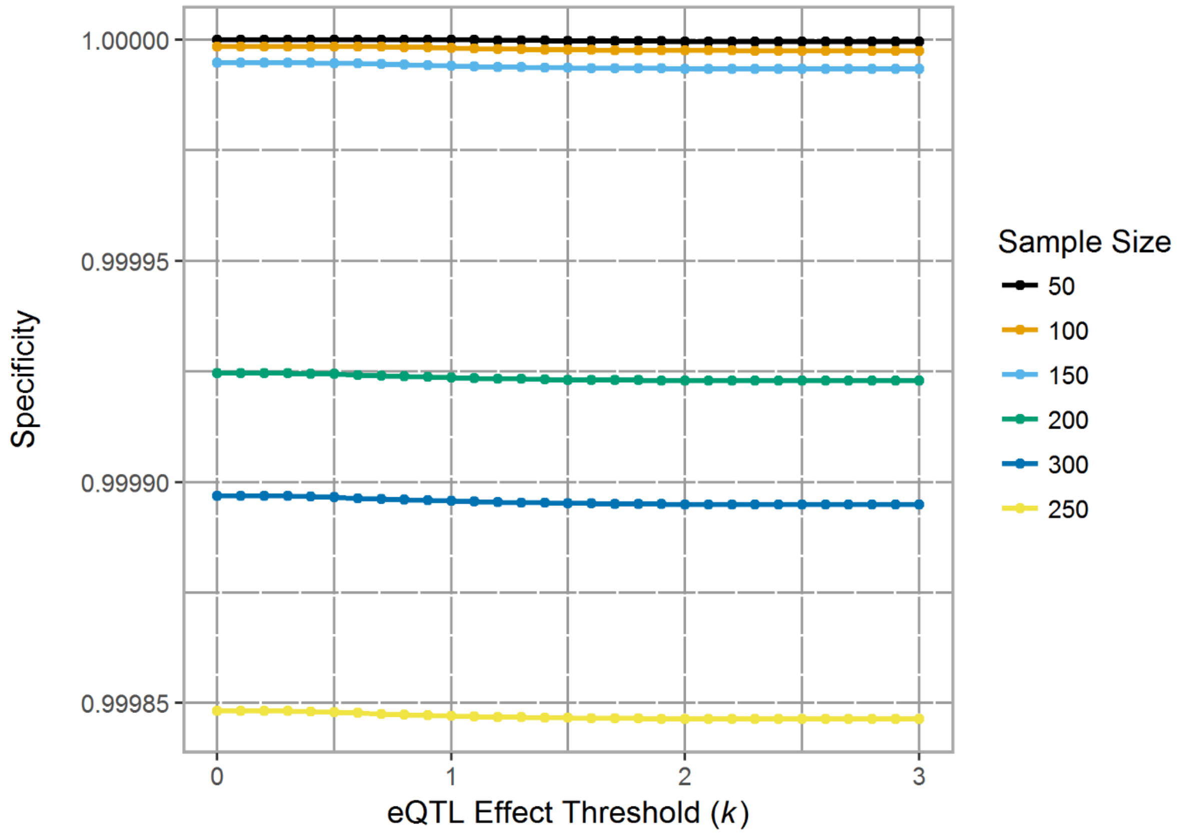

Figure S7. **The specificity to detect eQTLs at a range of effect size thresholds for Scenario 4: Lower expression level variance compared with residual variance (LV)**. A plot that shows the average specificity from 100 simulations in each sample size (n = 50, 100, 150, 200, 250, 300) to detect a range of eQTL effect size thresholds, k (0 – 3 in 0.1 increments) when eQTL expression levels were simulated with lower variance (σ^2_β) than model residual error variance (σ^2_ε). As sample size increases, the specificity to detect eQTLs decreases but note that n = 250 has lower specificity than n = 300 (i.e. this decrease is not monotonic). However, specificity is very high across all sample sizes and effect size thresholds with the lowest level of specificity (i.e. when n = 250, k = 3) being greater than 0.9997. For each sample size, there is no discernible decrease in specificity as the effect size threshold increase, unlike the increase in sensitivity.

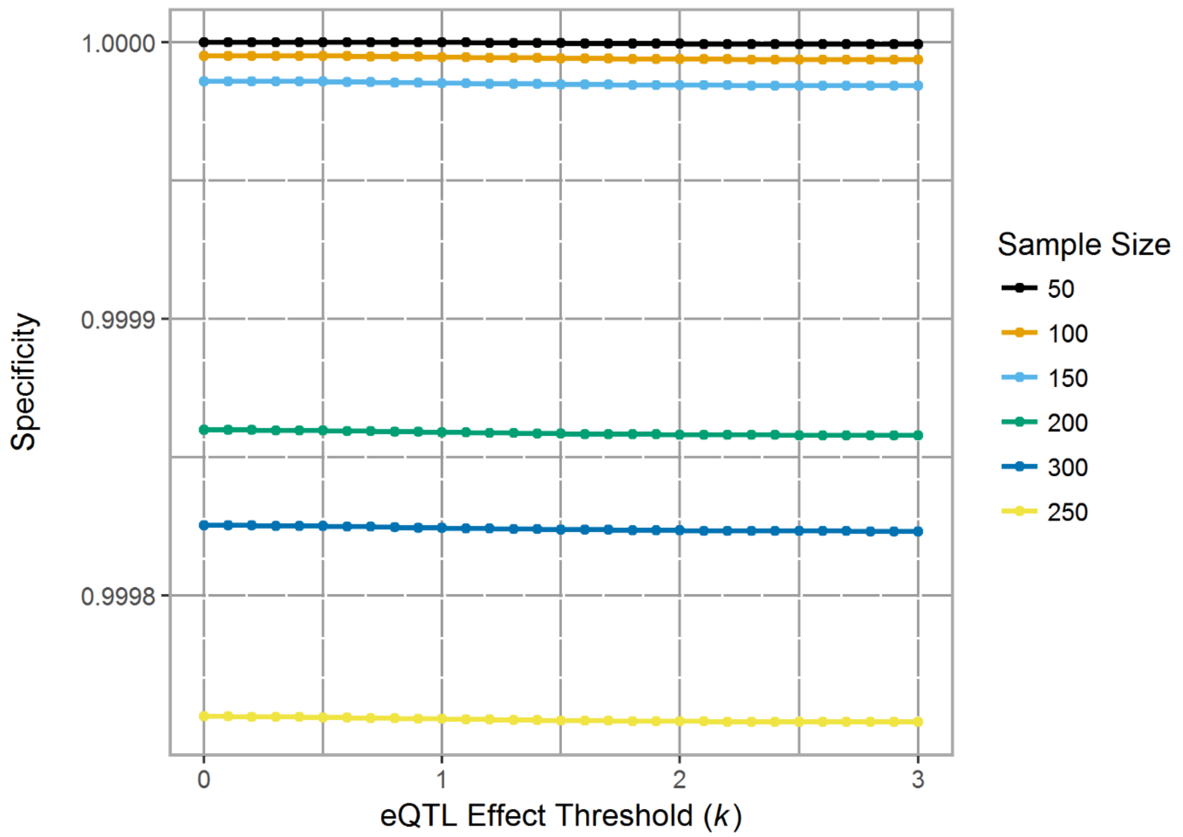

Figure S8. **The specificity to detect eQTLs at a range of effect size thresholds for Scenario 5: Dominance (Dom)**. A plot that shows the average specificity from 100 simulations in each sample size (*n* = 50, 100, 150, 200, 250, 300) to detect a range of eQTL effect size thresholds, *k* (0 – 3 in 0.1 increments) when dominance effect bias is introduced. As sample size increases, the specificity to detect eQTLs decreases but note that *n* = 250 has lower specificity than *n* = 300 (i.e. this decrease is not monotonic). However, specificity is very high across all sample sizes and effect size thresholds with the lowest level of specificity (i.e. when *n* = 250, *k* = 3) being greater than 0.9997. For each sample size, there is no discernible decrease in specificity as the effect size threshold increase, unlike the increase in sensitivity.

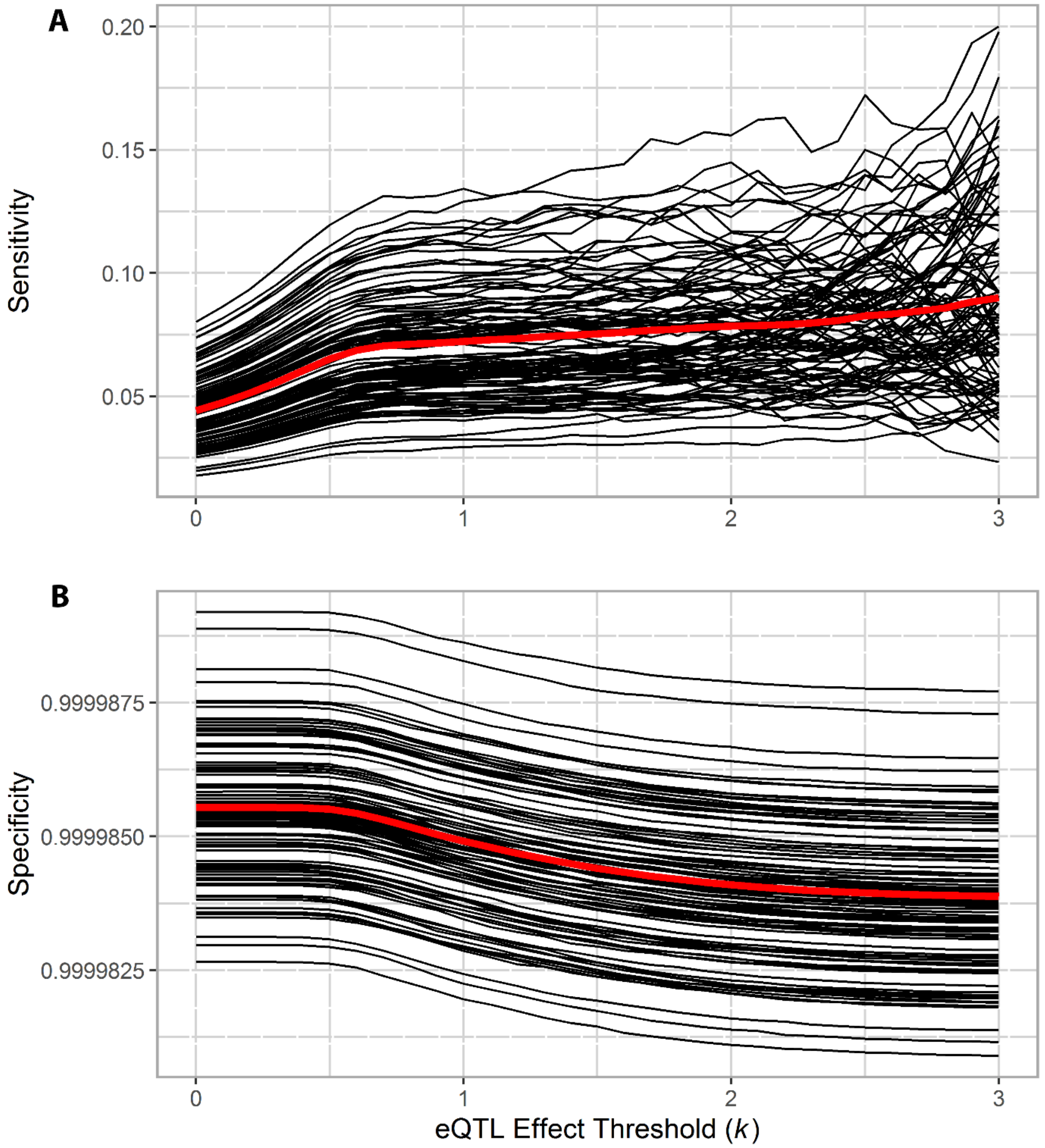

Figure S9. The sensitivity (A) and specificity (B) to detect eQTLs at a range of effect size thresholds for the scenario with linkage equilibrium (LE). Both plots show the sensitivity/specificity to detect a range of eQTL effect sizes when $n = 150$. Each thin black line represents the sensitivity/specificity from a single simulation with the calculated average in bold red. In terms of sensitivity, there is much variability between each simulation and at higher effect size thresholds ($k \geq 2$) within each simulation. For specificity, there too is variability between each simulation, however the curves are relatively smooth within each simulation. The averages can be seen to stabilise the variability for a smoother and centred curve.

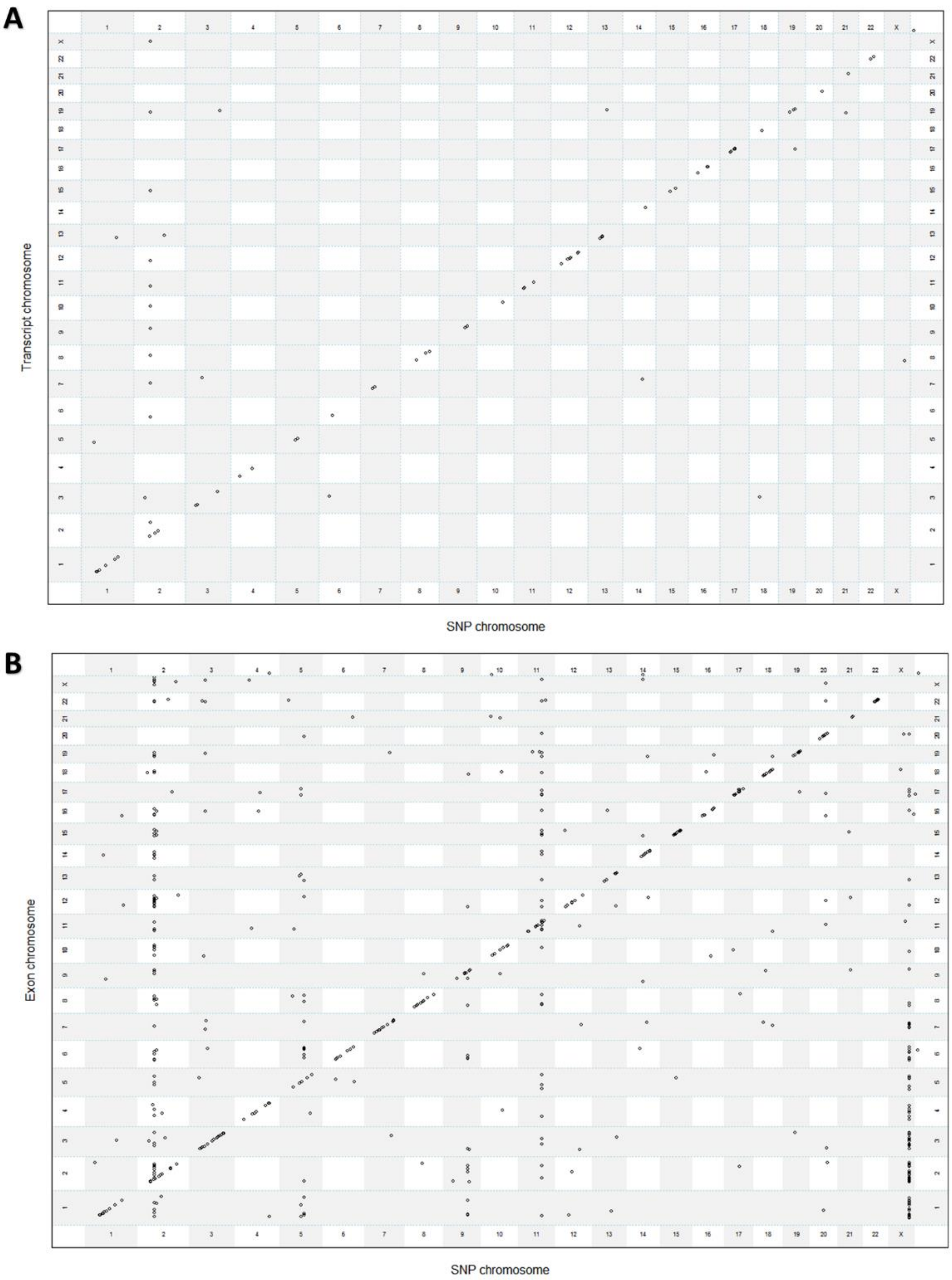

Figure S10. Genome-wide distribution of eQTL found in the frontal cortex (FCTX) at the (A) transcript-level and (B) exon-level. Each point in both plots represents an eQTL with the SNP chromosomes on the *x*-axis and the transcript chromosome on the *y*-axis. Note that these are the eQTLs after redundant SNPs were removed (i.e. these eQTLs are associated to haplotypes representing a block of SNPs in linkage disequilibrium). The clear diagonal line represents *cis*-acting eQTLs while the off-diagonal points represent *trans*-acting eQTLs. There

are vertical lines of eQTL points indicating that there are haplotypes or adjacent haplotypes that are associated with multiple exons. Less evident are horizontal lines of eQTL points, though more apparent on the exon-level (B) than on the transcript-level (A). The eQTL points along these horizontal lines suggest that there are genes associated with many haplotypes.

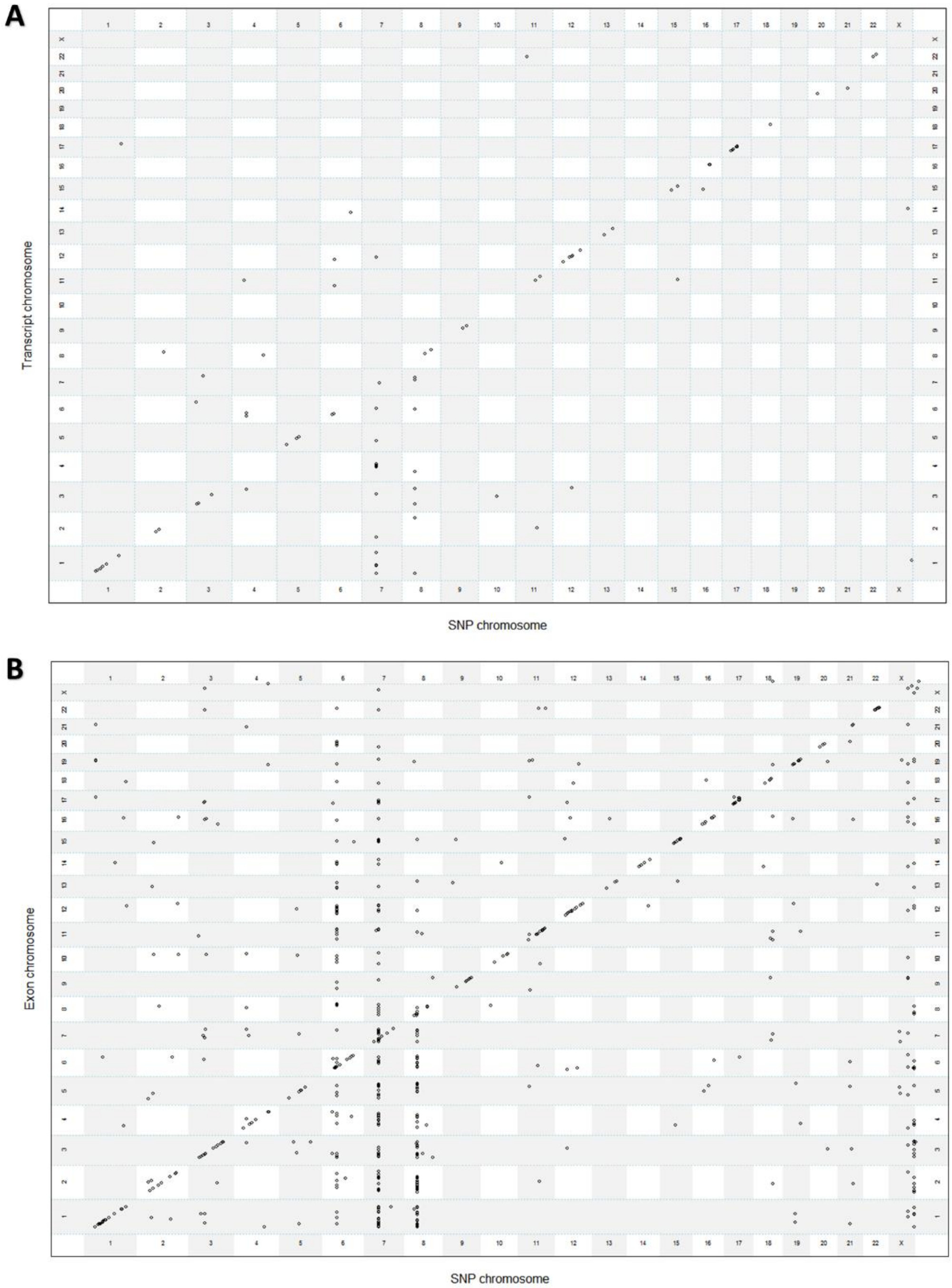

Figure S11. Genome-wide distribution of eQTLs found in the hippocampus (HIPP) at the (A) transcript-level and (B) exon-level. Each point in both plots represents an eQTL with the SNP chromosomes on the *x*-axis and the transcript chromosome on the *y*-axis. Note

that these are the eQTLs after redundant SNPs were removed (i.e. these eQTLs are associated to haplotypes representing a block of SNPs in linkage disequilibrium). The clear diagonal line represents *cis*-acting eQTLs while the off-diagonal points represent *trans*-acting eQTLs. There are vertical lines of eQTL points indicating that there are haplotypes or adjacent haplotypes that are associated with multiple exons.Less evident are horizontal lines of eQTL points, though more apparent on the exon-level (B) than on the transcript-level (A). The eQTL points along these horizontal lines suggest that there are genes associated with many haplotypes.

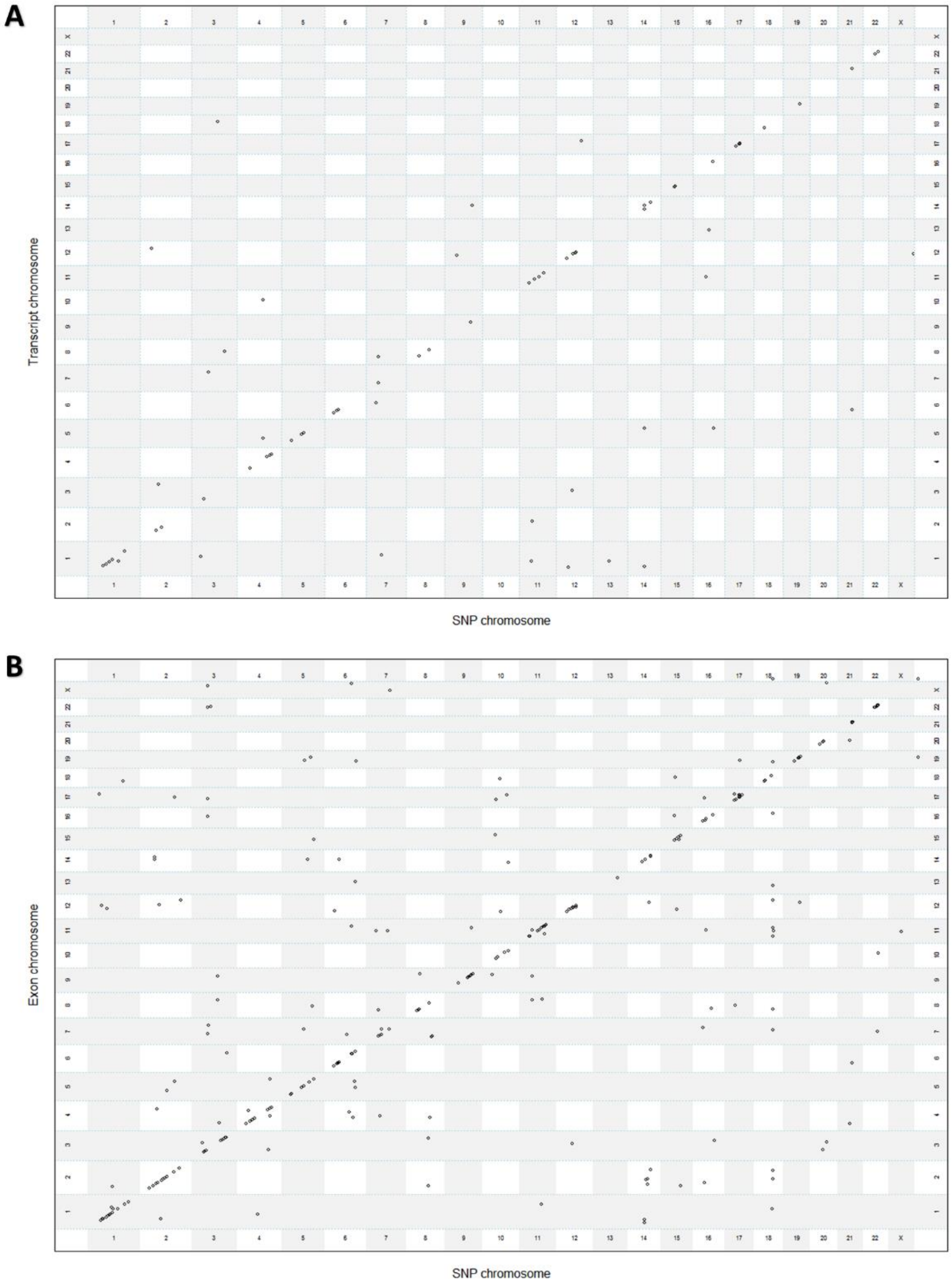

Figure S12. Genome-wide distribution of eQTLs found in the medulla (MEDU) at the (A) transcript-level and (B) exon-level. Each point in both plots represents an eQTL with the SNP chromosomes on the *x*-axis and the transcript chromosome on the *y*-axis. Note that these are the eQTLs after redundant SNPs were removed (i.e. these eQTLs are associated to haplotypes representing a block of SNPs in linkage disequilibrium). The clear diagonal line represents *cis*-acting eQTLs while the off-diagonal points represent *trans*-acting eQTLs. There are vertical lines of eQTL points indicating that there are haplotypes or adjacent haplotypes that are

associated with multiple exons.Less evident are horizontal lines of eQTL points, though more apparent on the exon-level (B) than on the transcript-level (A). The eQTL points along these horizontal lines suggest that there are genes associated with many haplotypes.

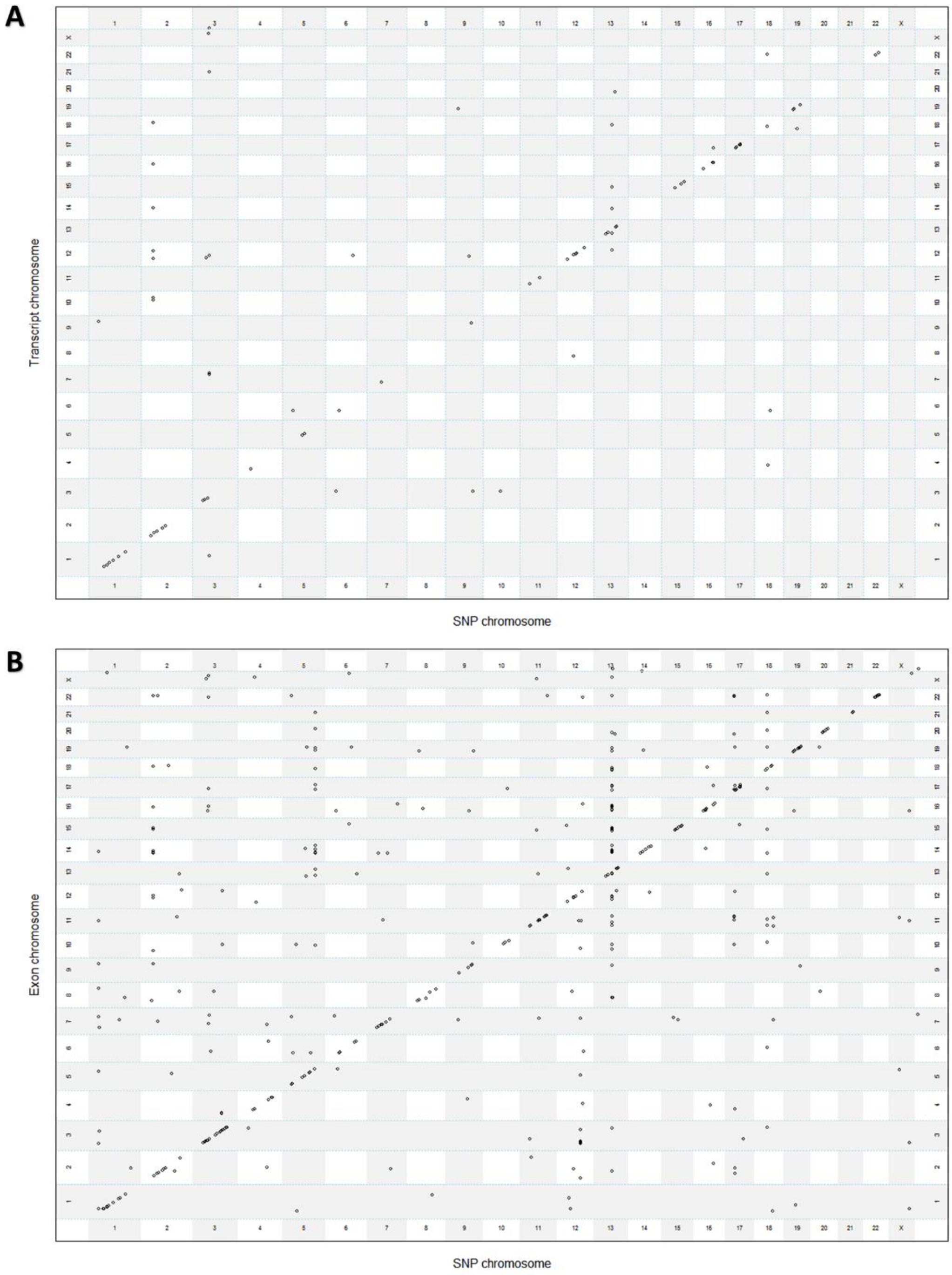

Figure S13. Genome-wide distribution of eQTLs found in the occipital cortex (OCTX) at the (A) transcript-level and (B) exon-level. Each point in both plots represents an eQTL with the SNP chromosomes on the *x*-axis and the transcript chromosome on the *y*-axis. Note that

these are the eQTLs after redundant SNPs were removed (i.e. these eQTLs are associated to haplotypes representing a block of SNPs in linkage disequilibrium). The clear diagonal line represents *cis*-acting eQTLs while the off-diagonal points represent *trans*-acting eQTLs. There are vertical lines of eQTL points indicating that there are haplotypes or adjacent haplotypes that are associated with multiple exons.. Less evident are horizontal lines of eQTL points, though more apparent on the exon-level (B) than on the transcript-level (A). The eQTL points along these horizontal lines suggest that there are genes associated with many haplotypes.

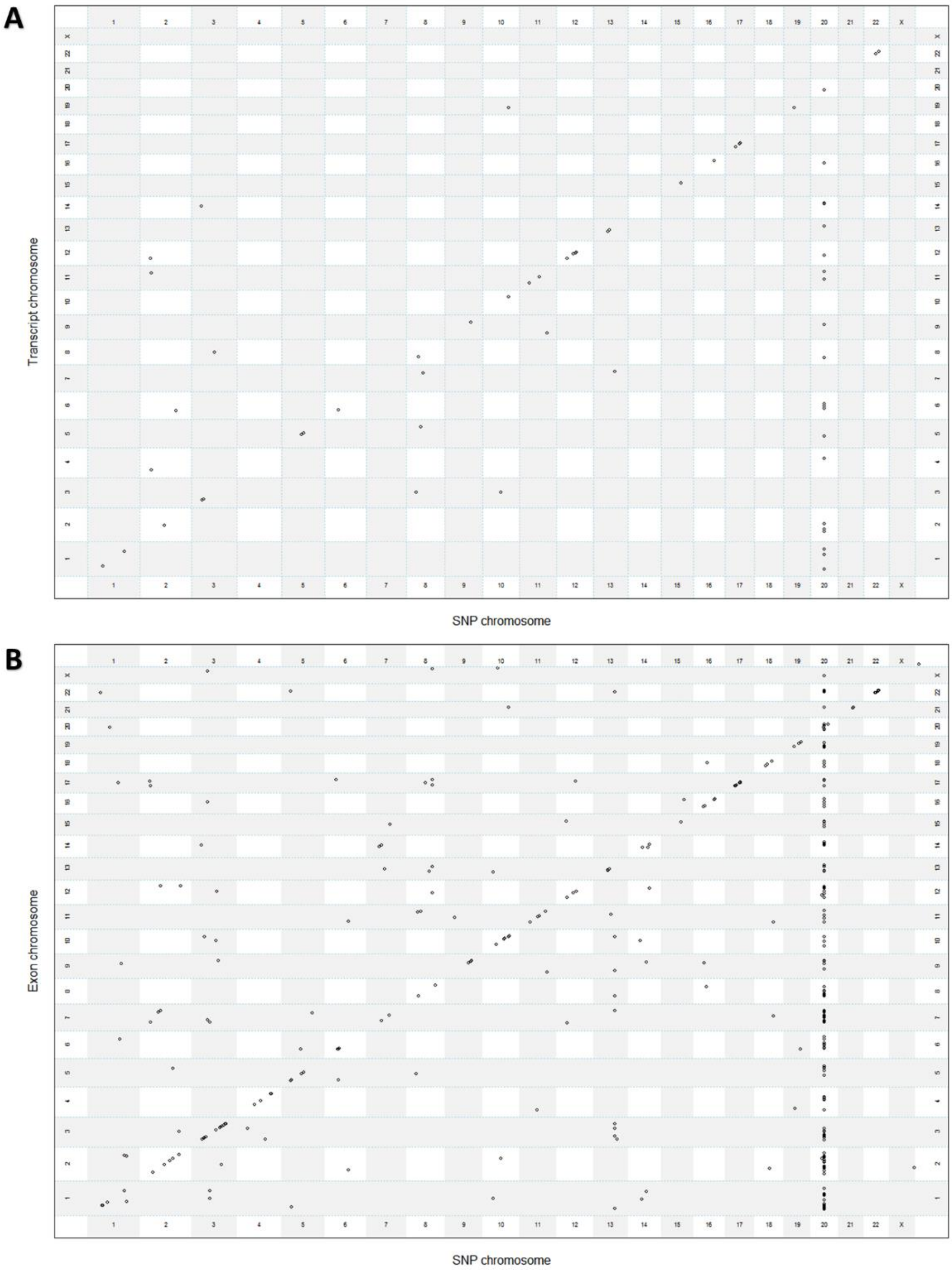

Figure S14. Genome-wide distribution of eQTLs found in the putamen (PUTM) at the (A) transcript-level and (B) exon-level. Each point in both plots represents an eQTL with the SNP chromosomes on the *x*-axis and the transcript chromosome on the *y*-axis. Note that these are the eQTLs after redundant SNPs were removed (i.e. these eQTLs are associated to haplotypes representing a block of SNPs in linkage disequilibrium). The clear diagonal line represents *cis*-acting eQTLs while the off-diagonal points represent *trans*-acting eQTLs. There are vertical lines of eQTL points indicating that there are haplotypes or adjacent haplotypes

that are associated with multiple exons.. Less evident are horizontal lines of eQTL points, though more apparent on the exon-level (B) than on the transcript-level (A). The eQTL points along these horizontal lines suggest that there are genes associated with many haplotypes.

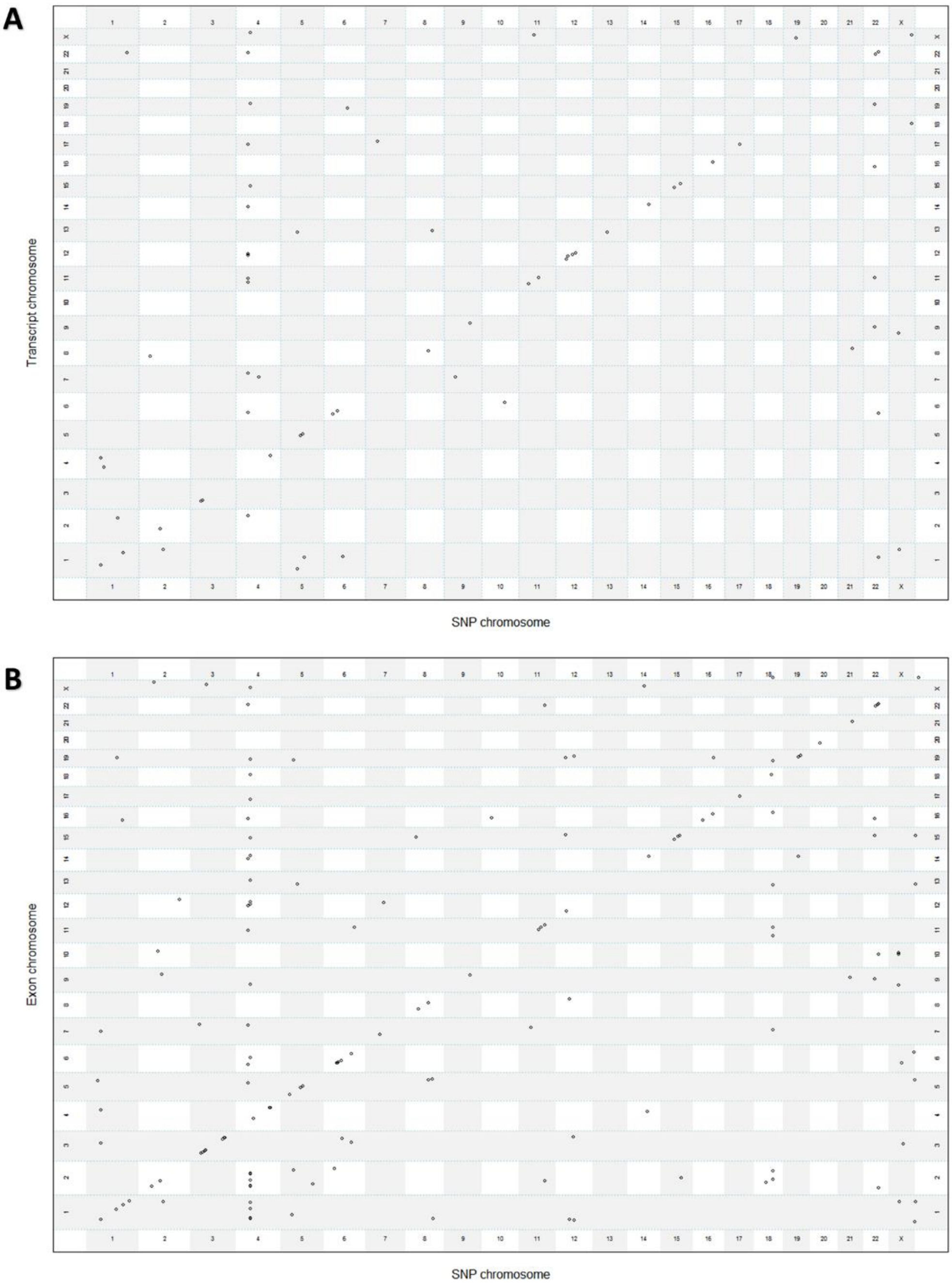

Figure S15. Genome-wide distribution of eQTLs found in the substantia nigra (SNIG) at the (A) transcript-level and (B) exon-level. Each point in both plots represents an eQTL with the SNP chromosomes on the *x*-axis and the transcript chromosome on the *y*-axis. Note that

these are the eQTLs after redundant SNPs were removed (i.e. these eQTLs are associated to haplotypes representing a block of SNPs in linkage disequilibrium). The clear diagonal line represents *cis*-acting eQTLs while the off-diagonal points represent *trans*-acting eQTLs. There are vertical lines of eQTL points indicating that there are haplotypes or adjacent haplotypes that are associated with multiple exons. Less evident are horizontal lines of eQTL points, though more apparent on the exon-level (B) than on the transcript-level (A). The eQTL points along these horizontal lines suggest that there are genes associated with many haplotypes.

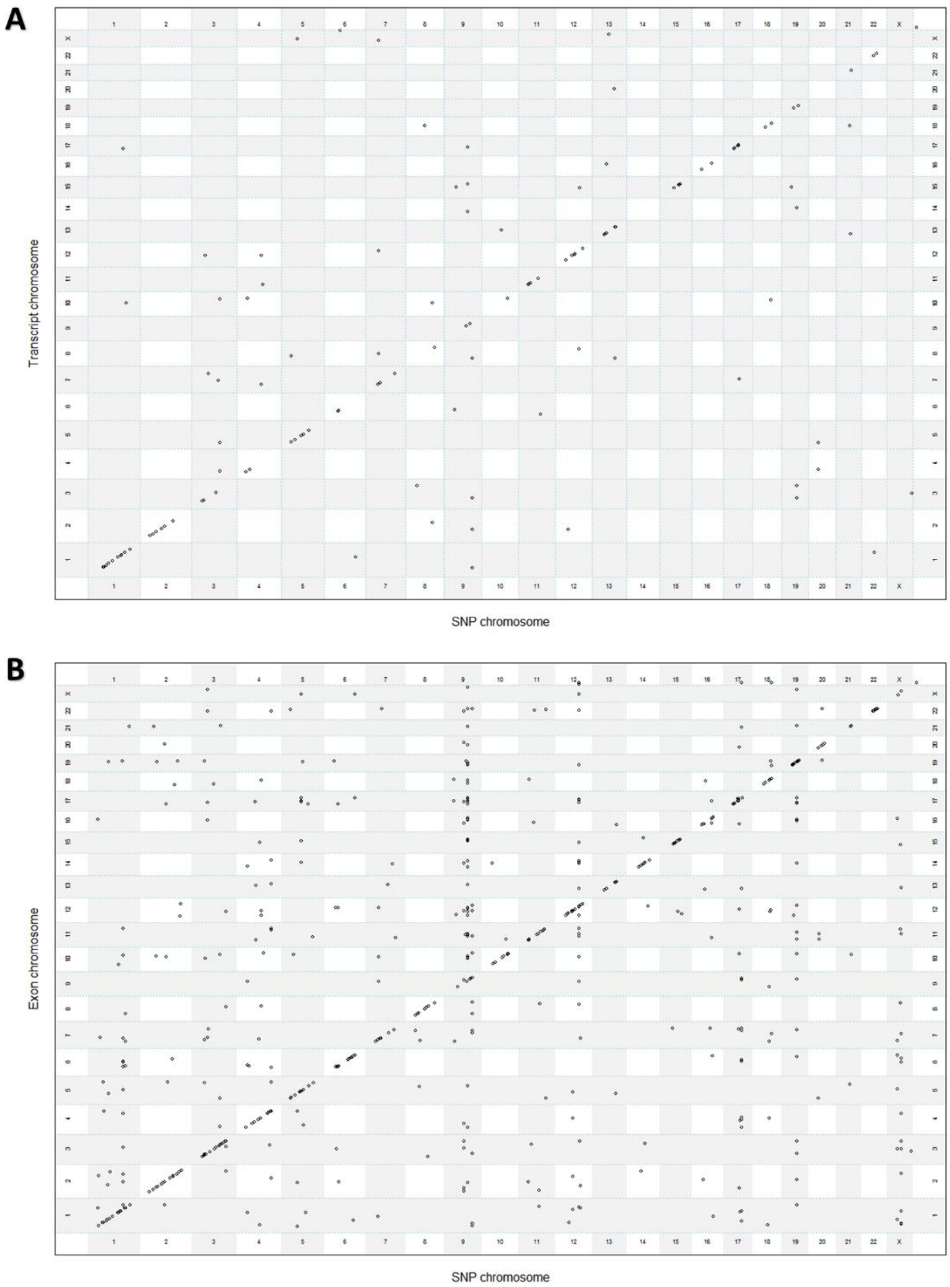

Figure S16. Genome-wide distribution of eQTLs found in the temporal cortex (TCTX) at the (A) transcript-level and (B) exon-level. Each point in both plots represents an eQTL with the SNP chromosomes on the *x*-axis and the transcript chromosome on the *y*-axis. Note that these are the eQTLs after redundant SNPs were removed (i.e. these eQTLs are associated to haplotypes representing a block of SNPs in linkage disequilibrium). The clear diagonal line represents *cis*-acting eQTLs while the off-diagonal points represent *trans*-acting eQTLs. There are vertical lines of eQTL points indicating that there are haplotypes or adjacent haplotypes

that are associated with multiple exons. Less evident are horizontal lines of eQTL points, though more apparent on the exon-level (B) than on the transcript-level (A). The eQTL points along these horizontal lines suggest that there are genes associated with many haplotypes.

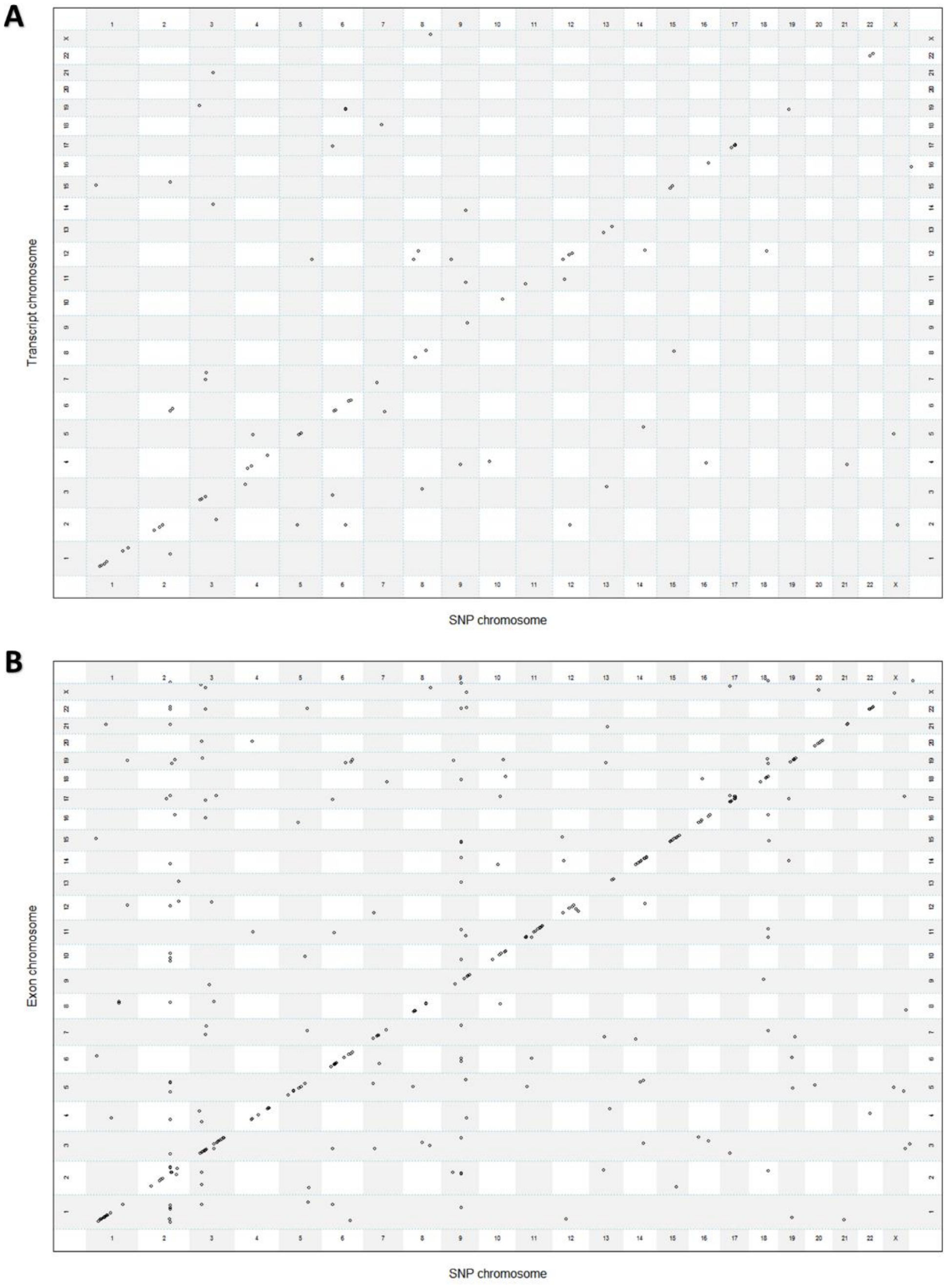

Figure S17. Genome-wide distribution of eQTLs found in the thalamus (THAL) at the (A) transcript-level and (B) exon-level. Each point in both plots represents an eQTL with the SNP chromosomes on the *x*-axis and the transcript chromosome on the *y*-axis. Note that these are the eQTLs after redundant SNPs were removed (i.e. these eQTLs are associated to

haplotypes representing a block of SNPs in linkage disequilibrium). The clear diagonal line represents *cis*-acting eQTLs while the off-diagonal points represent *trans*-acting eQTLs. There are vertical lines of eQTL points indicating that there are haplotypes or adjacent haplotypes that are associated with multiple exons. Less evident are horizontal lines of eQTL points, though more apparent on the exon-level (B) than on the transcript-level (A). The eQTL points along these horizontal lines suggest that there are genes associated with many haplotypes.

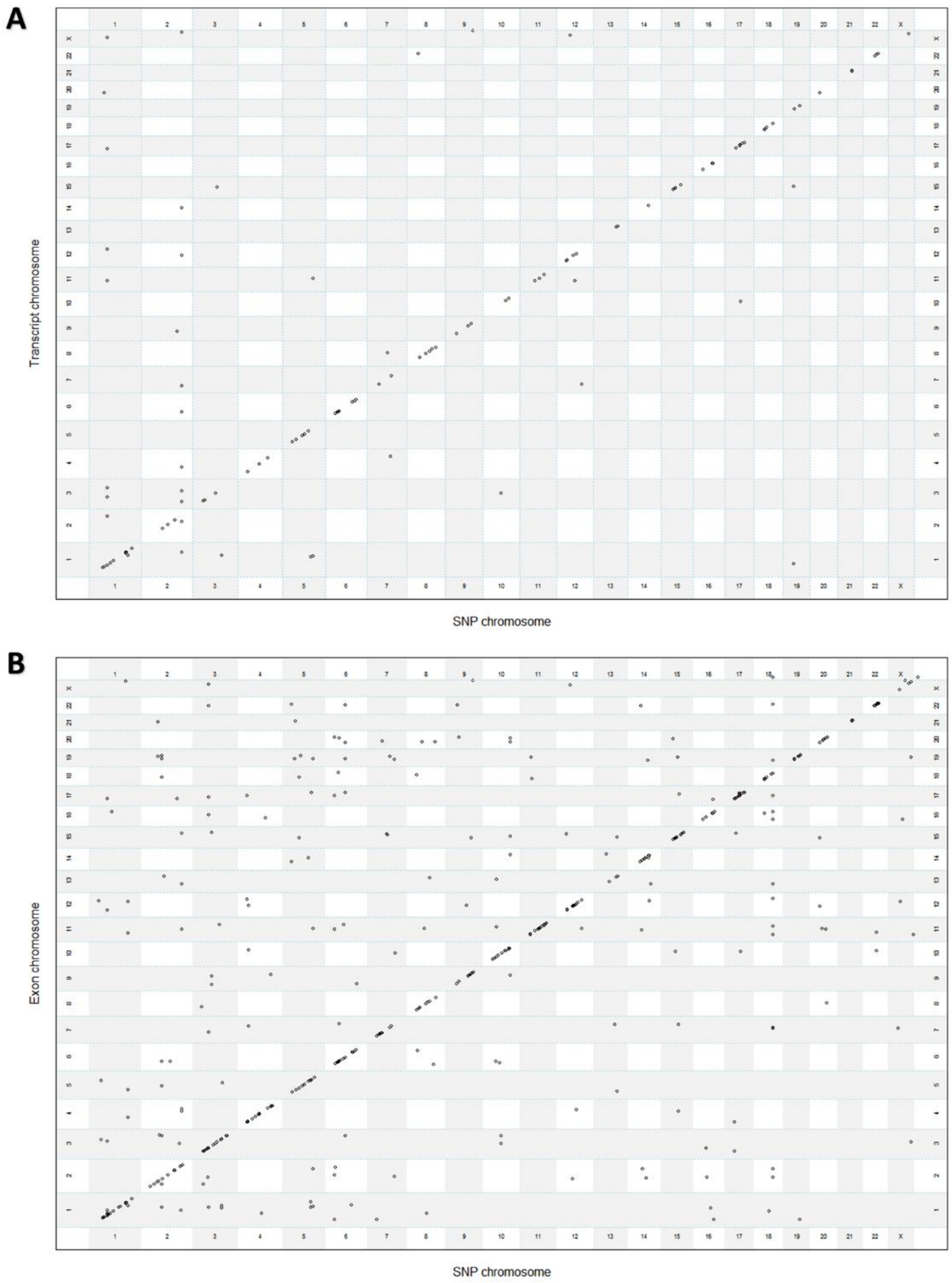

Figure S18. Genome-wide distribution of eQTLs found in the white matter (WHMT) at the (A) transcript-level and (B) exon-level. Each point in both plots represents an eQTL with the SNP chromosomes on the *x*-axis and the transcript chromosome on the *y*-axis. Note that these are the eQTLs after redundant SNPs were removed (i.e. these eQTLs are associated to haplotypes representing a block of SNPs in linkage disequilibrium). The clear diagonal line represents *cis*-acting eQTLs while the off-diagonal points represent *trans*-acting eQTLs. There are vertical lines of eQTL points indicating that there are haplotypes or adjacent haplotypes

that are associated with multiple exons. Less evident are horizontal lines of eQTL points, though more apparent on the exon-level (B) than on the transcript-level (A). The eQTL points along these horizontal lines suggest that there are genes associated with many haplotypes.

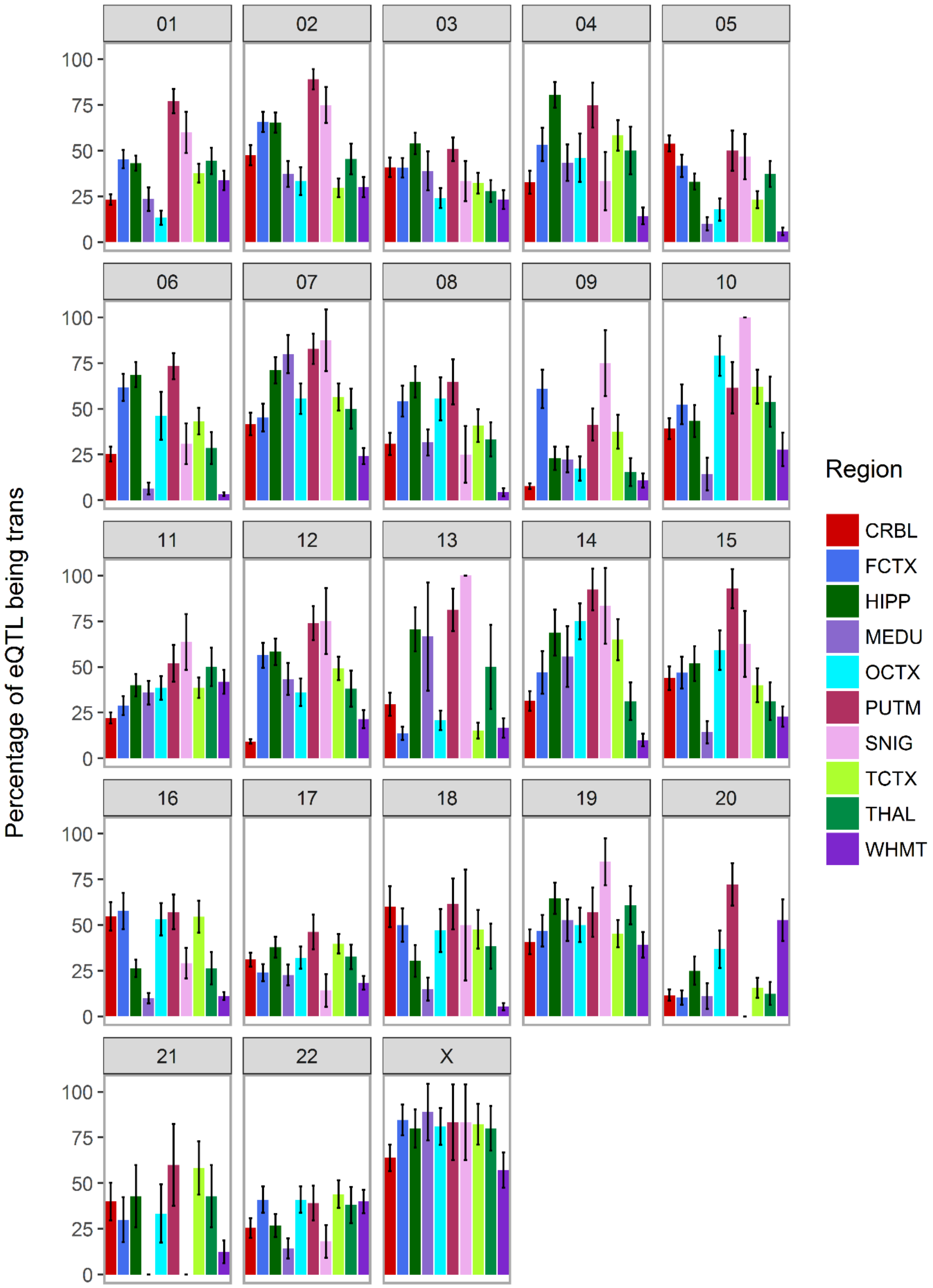

Figure S19. Percentage of exon-level eQTLs that are *trans*-acting for all ten brain regions on all 23 transcript chromosomes. These boxplots demonstrate the significant interaction between region and chromosome on the percentage of exon-level *trans*-acting eQTLs. For

example, across almost all the transcript chromosomes (except Chr 20), white matter (WHMT) one of the regions that shows a constantly low percentage of *trans*-acting eQTLscompared to other brain regions for that transcript. On the other hand, there are brain regions with more variability of *trans*-acting eQTL percentage across transcript chromosome (e.g. substantia nigra (SNIG)).

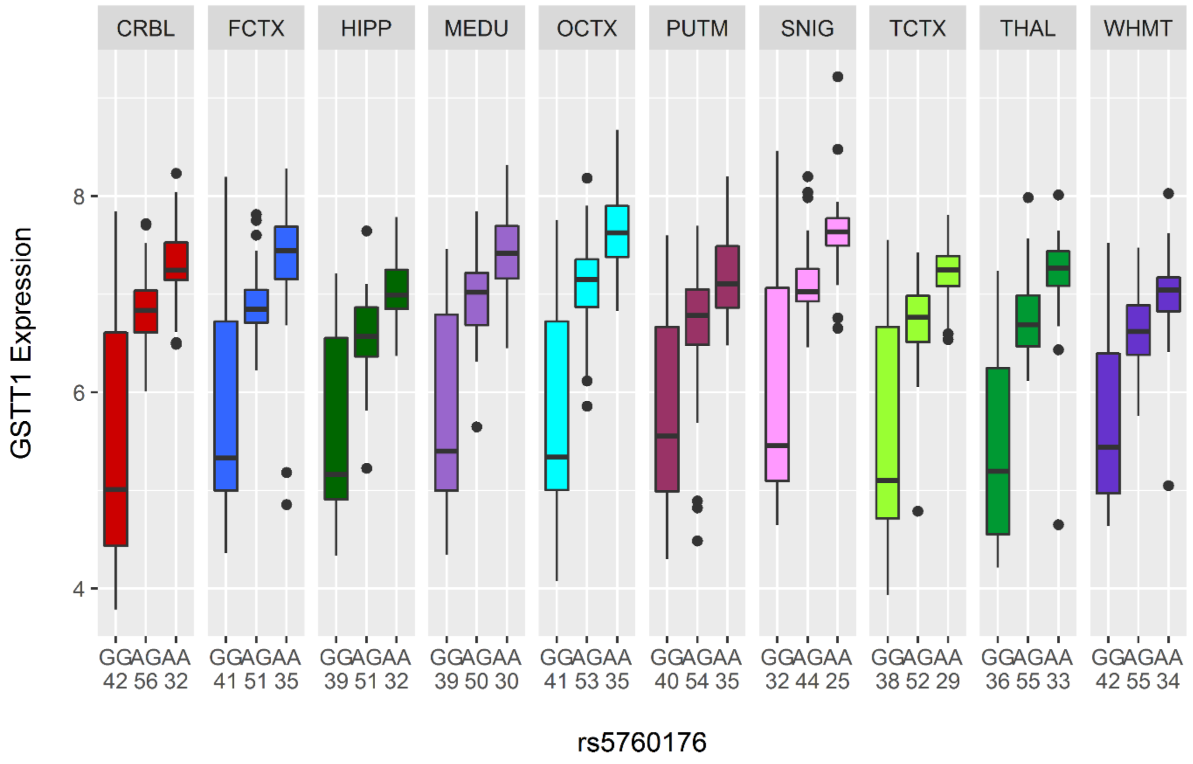

Figure S20. Boxplots of the effect of rs5760176 on *GSTT1* transcript expression levels. SNP rs5760176 effect on *GSTT1* expression for all ten brain regions. Increased expression was associated with the homozygous minor allele (AA) in all ten regions.

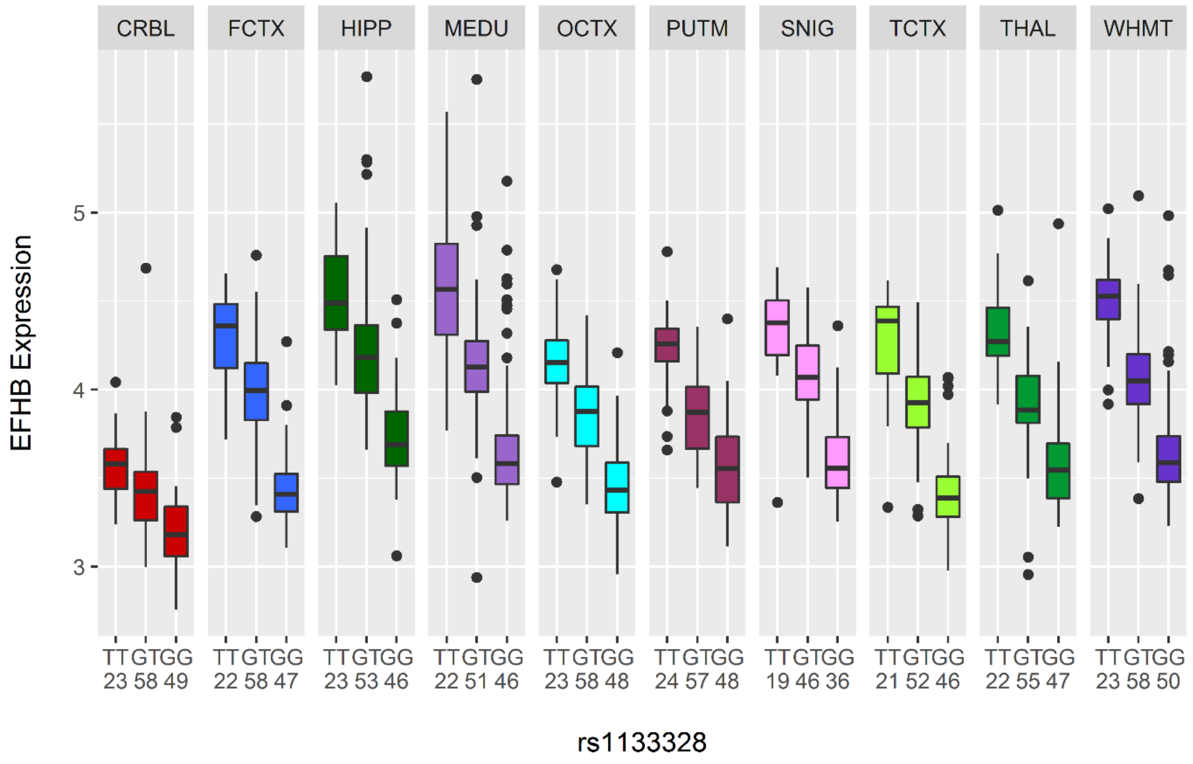

Figure S21. Boxplots of the effect of rs1133328 on the *EFHB* transcript expression levels. SNP rs1133328 effect on *EFHB* expression for all ten brain regions. Decreased expression was associated with the homozygous genotype (GG) in all ten regions. CRBL shows a different pattern of lower expression.

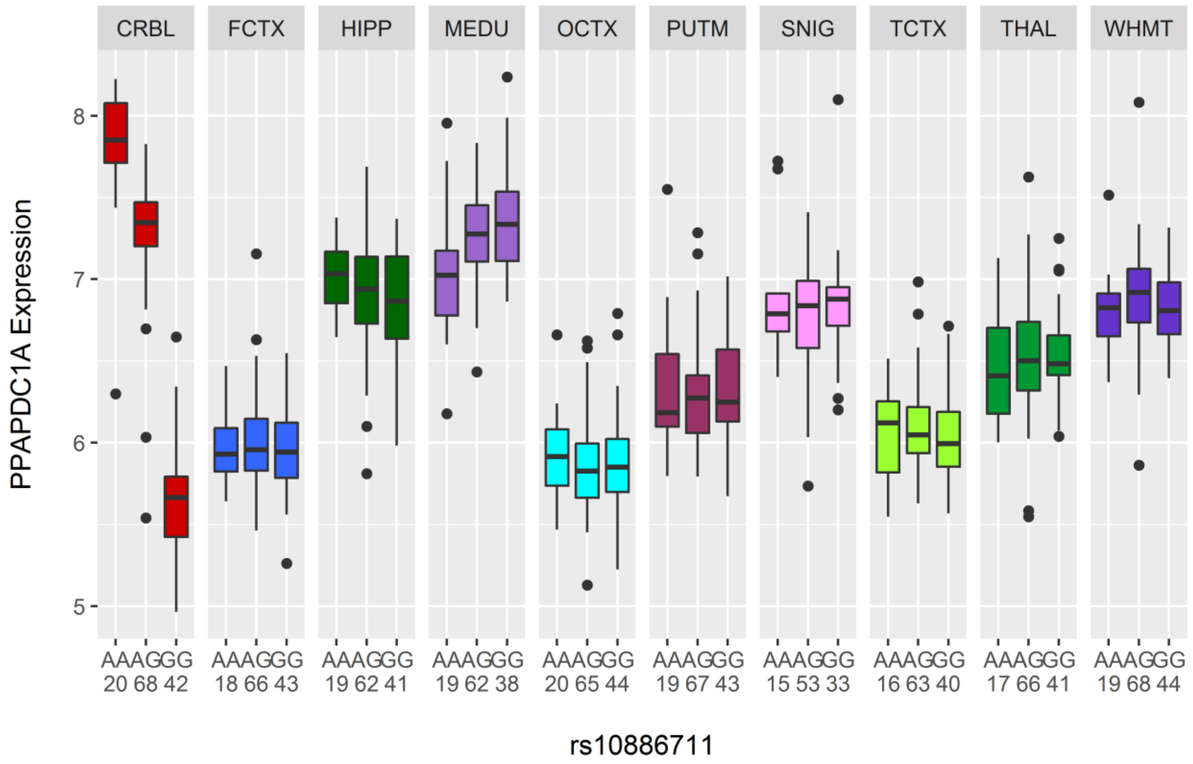

Figure S22. Boxplots of the effect of SNP rs10886711 on *PLPP4* (*PPAPDC1A*) expression levels. SNP rs10886711 effect on *PLPP4* expression for all ten brain regions. The GG homozygous genotype is associated with a decrease in expression level. However, this decrease was only significant in CRBL.

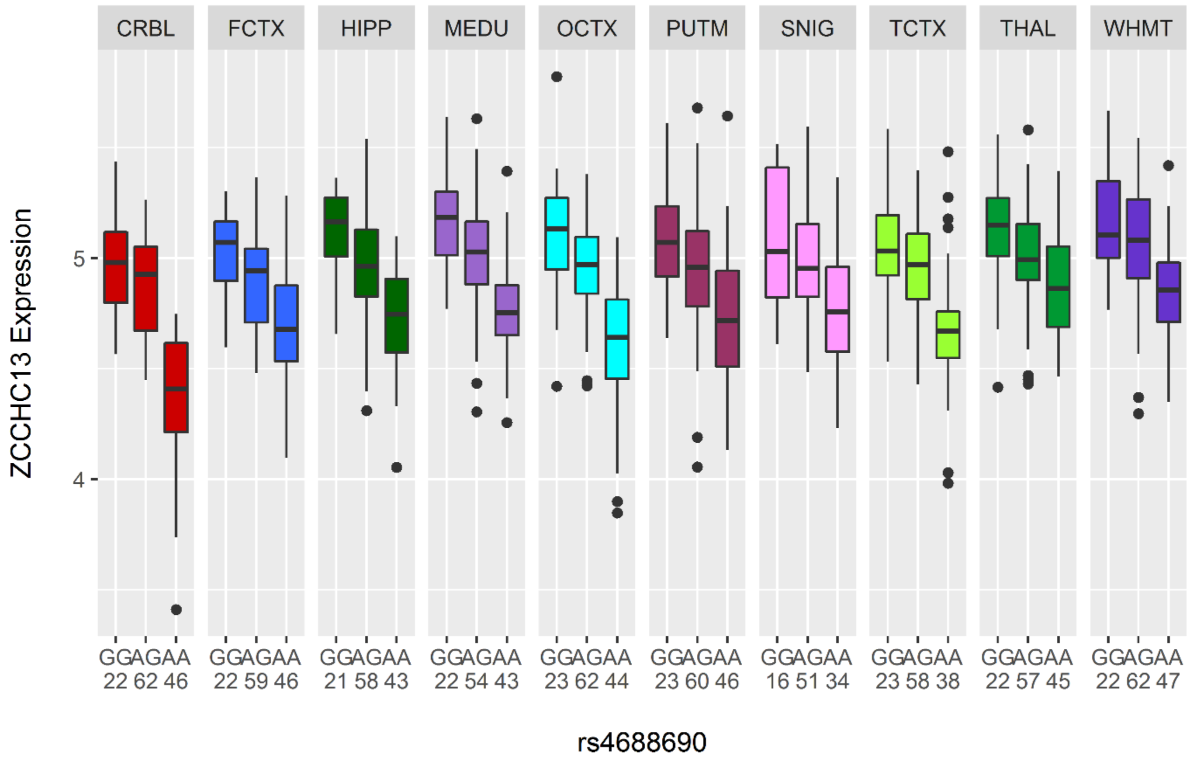

Figure S23. Boxplots of the effect of SNP rs4688690 on ZCCHC13 expression levels. SNP rs4688690 effect on ZCCHC13 expression for all ten brain regions. The AA homozygous genotype is associated with a decrease in expression level of ZCCHC13. This decrease is more obvious than the rs10886711-PPAPDC1A (Figure S22) across all regions, however, it is also only significant in CRBL.
